# Scoping review of food safety at transport stations in Africa

**DOI:** 10.1136/bmjopen-2021-053856

**Published:** 2021-11-25

**Authors:** Busisiwe Purity Ncama, Desmond Kuupiel, Sinegugu E Duma, Gugu Mchunu, Phindile Guga, Rob Slotow

**Affiliations:** 1 School of Nursing and Public Health, College of Health Sciences, University of KwaZulu-Natal, Durban, South Africa; 2 Research for Sustainable Development Consult, Sunyani, Ghana; 3 School of Life Sciences, College of Agriculture, Engineering and Science, University of Kwazulu-Natal, Pietermaritzburg, South Africa

**Keywords:** public health, public health, health & safety

## Abstract

**Objective:**

The WHO has declared food safety as a public health concern. Transport hubs such as taxi ranks, bus stations and other transport exchange sites are major food trading/purchasing sites, particularly in Africa. Research evidence is needed to improve food safety policies and ensure consumption of safe food, owing to the increasing burden of foodborne diseases, particularly in the WHO Africa Region. We systematically mapped and described research evidence on food safety at transport stations in Africa.

**Design:**

A scoping review guided by the Arksey and O’Malley framework.

**Data sources:**

We searched for original research articles in PubMed, Web of Science, and EBSCOhost (Academic search complete, CINAHL with Full-text and Health Source), SCOPUS, and Google Scholar from their inception to 25 October 2020.

**Eligibility criteria for selecting studies:**

We included studies that focused on food safety, involved transport stations, involved African countries and were published in English.

**Data extraction and synthesis:**

Data extraction was performed by two reviewers using a piloted-tested form. Thematic analysis was used to organise the data into themes and subthemes, and a narrative summary of the findings is presented.

**Results:**

Of the total 23 852 articles obtained from the database searches, 16 studies published in 6 countries met the inclusion criteria. These 16 studies were published between 1997 and 2019, with the most (5) in 2014. Of the 16 studies, 43.8% (7) were conducted in South Africa, 3 studies in Ghana, 2 in Ethiopia and 1 study each in Nigeria, Kenya, Lesotho and Zambia. Most (44.4%) of the included studies focused on microbial safety of food; few studies (22.2%) focused on hygienic practices, and one study investigated the perspective of consumers or buyers. Microbes detected in the foods samples were *Salmonella* spp, *Escherichia coli*, *Shigella* spp, *Bacillus* sp, *Staphylococcus aureus*, which resulted mainly from poor hygiene practices.

**Conclusions:**

There is limited research that focused on food safety at transport stations in Africa, especially on aspects such as hygiene practices, food storage and occupational health and food safety. Therefore, we recommend more research in these areas, using various primary study designs, to inform and improve food safety policies and practices for transport stations in African countries alongside improving access to clean water/handwashing facilities, and undertaking structural changes to facilitate behaviours and monitoring for unintended consequences such as livelihoods of vulnerable populations.

**Strengths and limitations of this study**

- To the best of our knowledge, this is the first scoping review to systematically explore literature and describe research evidence on food safety at transport stations as well as identify gaps for future research in Africa.
- This scoping review’s evidence sources were searched using a systematic approach, and duplicate screening.
- This review is limited to Africa as well as English language publications.

## Background

The WHO estimates that more than 600 million people fall sick (almost 1 in 10 people) with foodborne diseases annually, of which nearly 420 000 people die, and about 33 million years of healthy lives are lost every year worldwide.^1 2^ The burden of foodborne diseases is estimated to be highest in the WHO African and South-East Asia Regions, mainly occurring among vulnerable populations such as infants, young children, pregnant women, older people, poor people and individuals with underlying illnesses.^3^ Food contamination mostly results throughout the food supply chain (from the procedures used in processing the foods, inadequate storage temperatures, unhygienic practices by food handlers, poor sanitation at cooking places/vending areas, poor waste management and inadequate treatment of leftovers).^4^

Unsafe food has negative implications on health systems, and affects the development and national economies of countries, as well as trade.^3^ Therefore, eating unsafe foods poses a significant public health threat. To avert the consequences of unsafe food on health systems, and to sustain national economies, development, trade and tourism,^5 6^ the WHO in 2006 declared food safety as a global public health concern.^7 8^ ‘Food safety refers to routines in the preparation, handling and storage of food meant to prevent foodborne illness and injury’.^5^ To reduce the incidence of food-related diseases, particularly in high burden regions, the observations of food safety measures/precautions at all levels of the food processing chain, including the places where food is prepared and sold, are critical.^9 10^

Like other WHO Regions, especially in low-income and middle-income countries, food trading in the Africa Region takes place at several formal and informal places, such as in the markets, restaurants, streets, open spaces in academic institutions, and transport stations (taxi ranks, bus stations, lorry parks), and other transport exchange sites. Food vending at public spaces serves as a source of livelihood,^6 10 11^ and more than two billion people eat food sold at various vending locations. including transportations stations on daily basis globally.^12 13^ To this end, evidence is essential to inform in-country policies/guidelines, and further research, to ensure that food prepared and sold at transport stations promotes livelihoods, nutrition, food safety, and environmentally sustainable practices. This scoping review systematically mapped literature focused on food safety at transport stations in Africa, to summarise evidence and identify gaps.

## Methods

### Scope of the review

The Arksey & O’Malley framework (research question identification; identifying relevant studies; selection of study; data charting, collating, and summarising and reporting the findings^14 15^ was employed to scope and synthesise literature to answer the question—what evidence exists on food safety at transport stations in Africa? This review’s study protocol was developed a priori.^16^ This study included published peer-reviewed articles that reported findings from any African country/countries, focused on food safety, and involved transport stations. However, this study was limited to English publications (due to lack of expertise in other international languages), and primary study designs A detailed description of this scoping review study eligibility criteria is captured in the published protocol.^16^ We followed the Preferred Reporting Items for Systematic Reviews and Meta-analyses (PRISMA) extension for Scoping Reviews checklist to report this study.^17^

### Identify relevant studies

We searched for primary research articles relating to food safety at transport stations in PubMed, Web of Science and EBSCOhost (Academic search complete, Cumulated Index to Nursing and Allied Health Literature (CINAHL) with Full-text, and Health Source), SCOPUS, and Google Scholar from their inception to 25 October 2020. To enable the capturing of all relevant articles, a comprehensive search strategy (developed in consultation with an expert librarian) consisting of keywords, Boolean terms (AND/OR), and Medical Subject Heading terms, was used for the electronic database search (online supplemental file 1). Syntax was modified appropriately where needed. Filters such as date and study design were not applied during the literature search in the databases. DK and PG independently conducted the database search and title screening, and imported all potentially eligible articles onto an EndNote Library. The reference lists of all included articles were also screened for potentially relevant articles using the same approach.

10.1136/bmjopen-2021-053856.supp1 Supplementary data

### Selection of articles

Prior to the abstract screening, the ‘find duplicates’ function in EndNote was used to find all duplicate articles, and they were removed from the library. A screening form was developed in Google forms, using this study’s eligibility criteria, for the abstract and full text screening phases. Two reviewers (coauthors) independently screened the abstracts as well as the full text articles. Discrepancies that arose during the abstract stage were resolved by discussion among the review team until a consensus was reached. At the full text screening phase, discrepancies were resolved by a third reviewer. All the additional articles identified from the reference list of the included articles equally underwent full text assessment. The PRISMA flow diagram was employed to account for all the articles involved.^18^

### Charting the data

A data extraction form was designed consisting of the following: author(s) and publication details, country of study, study design, study setting, study population, sample size, sex, study findings and recommendations. To ensure consistency and reliability, two reviewers piloted the data extraction sheet using a random sample of three included studies. The pilot testing of the form also enabled the review team to discuss discrepancies, and to revise the data extraction form prior to its final usage. Subsequently, 2 reviewers conducted the data extraction for the remaining 15 included studies using both inductive and deductive approaches. The review team resolved all discrepancies at this stage through discussion.

### Collating, summarising and reporting the results

This study subsequently employed thematic analysis, and collated all the emerging themes and subthemes relating to food safety. A summary of the findings from the included studies is presented narratively.

### Patient and public involvement

No patient involved.

## Results

**Figure 1:**
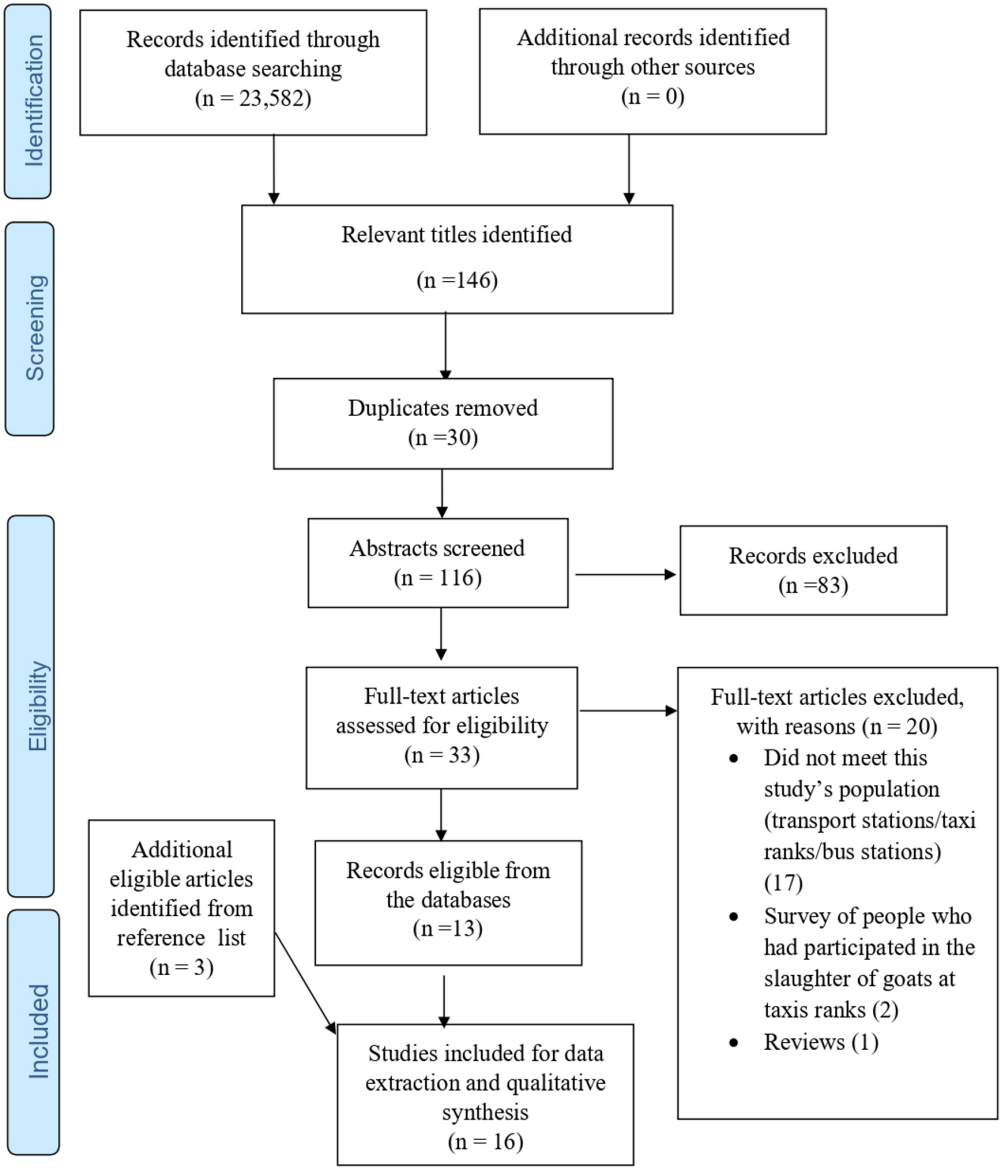
Preferred Reporting Items for Systematic Reviews and Meta-Analyses 2009 flow diagram.

Of the 23 852 articles obtained from the database searches (see figure 1 flow diagram), 146 articles met the eligibility criteria at the title screening stage. Using EndNote “Find Duplicates” function, 30 duplicates were found and removed before abstract screening was conducted. Subsequently, 83 articles were removed at the abstract screening, and 20 at full text (17 of these did not include transport stations/taxi ranks/bus stations, but did involve sale from market centres, public places, chop bars, mini restaurants, major streets and sidewalks, and were excluded). Finally, 13 studies were included, and, from a manual search of their reference lists, a further 3 articles were added, giving a total of 16 articles for further analysis.

### Characteristics of the included studies

**Figure 2:**
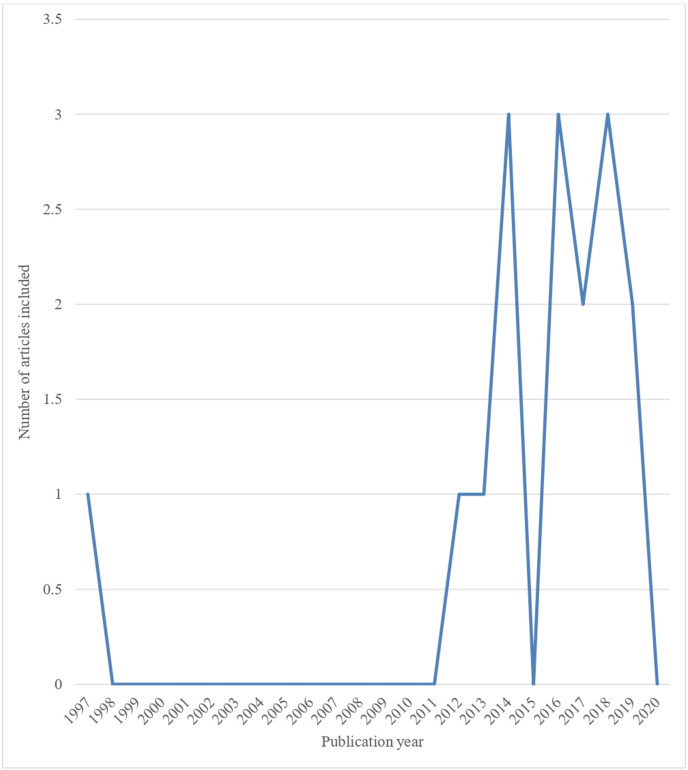
Trend of published studies relating to food safety at transport station in Africa.

Table 1A, B presents a summary of the characteristics of the included studies. Of the 16 included studies, about 44 (43.8%) were conducted in South Africa,^19–25^ 3 (18.8%) in Ghana,^4 26 27^ 2 (12.5%) in Ethiopia^28 29^ and 1 (6.2%) each in Nigeria,^30^ Kenya,^31^ Lesotho^32^ and Zambia.^33^ Most of the studies were published in the last 6 years; however, no published study was found in 2015 and 2020 (figure 2). Fifteen (93.8%) of the included studies were cross-sectional studies, and one (6.2%) was a mixed-method study. Of the 16 included studies, 50.0% reported on microbial safety of food^4 19 23 27–29 33^ and 25.0% reported hygiene practices of food handlers/vendors.^6 21 30 31^ One included study each reported on the following: occupational health and food safety risk^24^; knowledge of hygiene practice^26^; hygiene practices of food handlers/vendors and microbial safety^25^; and knowledge of food safety measures and hygiene practice by food handlers/vendors.^32^

**Table 1:** Characteristic of the included sources of evidence

Author, year	Country	City/town	Study design	Study setting	Study population	Sample size	Sex of vendors	Outcome reported
(A)								
Oguttu *et al*, 2014^19^	South Africa	Tshwane Metropole, Gauteng Province	Mixed-methods study	Taxi rank	Vendors selling Ready-to-eat chicken	100 samples of Ready-to-eat chicken	Females	Microbial safety of food
Mafune *et al*, 2016^20^	South Africa	Thohoyandou, Limpopo Province	Cross-sectional study	Taxi rank, bus station, shopping mall, and street stalls	Food samples from street vendors	28 samples	Not specified	Microbial safety of food
Kibret and Tadesse, 2013^28^	Ethiopia	Bahir Dar Town	Cross-sectional study	Main roads sites, bus station, groceries, taxi ranks	Ready-to-eat white lupin sample from vendors	40 samples (200 grams of white lupin)	Not specified	Microbial safety of food
Abakari *et al*, 2018^27^	Ghana	Tamale, Northern Region	Cross-sectional study	Taxi rank, bus stops, transport yard, and timber Market	Ready-to-eat salad samples from food vendors	30 salad samples	Not specified	Microbial safety of food
Aluko *et al*, 2014^30^	Nigeria	Ile Ife, southwestern Nigeria	Cross-sectional study	Car parks	Food vendors	160 (117 stationery and 43 mobile vendors)	Males and females	Hygiene practices of food handlers/vendors
Odundo *et al*, 2018^31^	Kenya	Not specified	Cross-sectional study	Major bus stops, markets, shopping areas, construction sites, and commercial areas	Food vendors	130	Males and females	Hygiene practices of food handlers/vendors
Kok and Balkaran, 2014^21^	South Africa	Durban, KwaZulu-Natal Province	Cross-sectional study	Transport exchange site	Food vendors	29	Not specified	Hygiene practices of food handlers/vendors
Letuka *et al*, 2019^32^	Lesotho	Maseru	Cross-sectional study	Taxi ranks	Food vendors	141 (48 food handlers and 93 consumers)	Male and female	Knowledge of food safety measures and hygiene practice by food handlers/vendors
Eromo *et al*, 2016^29^	Ethiopia	Hawassa City	Cross-sectional study	Bus station	Food samples from street food vendors	72 samples from six food items	Not specified	Microbial safety of food
(B)								
McArthur-Floyd *et al*, 2016^26^	Ghana	Madina (Accra), Greater Accra Region	Cross-sectional study	Taxi rank, and transport exchange sites	Food vendors	200	Males and females	Knowledge of hygiene practice
Hill *et al*, 2019^6^	South Africa	Cape Town	Cross-sectional study	Train, bus stations, and taxi ranks, community centres, market	Food vendors	831	Males and females	Hygiene practices of food handlers/vendors
Mazizi *et al*, 2017^23^	South Africa	Alice (Nkonkobe) and King William’s Town (Buffalo City), Eastern Cape province	Cross-sectional study	Taxi rank and bus stations	Street food vendors	136 food samples- cooked and raw.	Not specified	Microbial safety of food
Qekwana *et al*, 2017^24^	South Africa	Tshwane Metropolitan Municipality, Gauteng Province	Cross-sectional study	Taxi ranks and Informal markets	Traditional goat slaughter	105 people	Males and females	Occupational health and food safety risk
Flego and Sakyi, 2012^4^	Ghana	Kumasi, Ashanti Region	Cross-sectional study	Bus terminals	Food samples from vendors	60 food samples	Not specified	Microbial safety of food
Tshipamba *et al*, 2018^25^	South Africa	Johannesburg	Cross-sectional study	Taxi ranks and streets	Meat samples from vendors	115 meat samples	Not specified	Hygiene practices of food handlers/vendors, and microbial safety of food
Jermini *et al*, 1997^33^	Zambia	Not specified	Cross-sectional study	Bus park/station and large market	Samples of raw, processed, and cooked Foods from street food vendors	Not specified	Not specified	Microbial safety of food

### Findings from the included studies

#### Microbial safety of food

Of the nine included studies that reported findings on microbial safety of food, 44.4% were conducted in South Africa,^19 20 23 25^ 22.2% each in Ghana^4 27^ and Ethiopia^28 29^ and the last 11.1% in Zambia.^33^ Seven of the eight studies reported unacceptable levels of microbes in the food.^4 19 20 23 27–29 33^ Table 2A–C presents a summary of the key findings as well as the sample type, analytic approach and the microbes reported.

**Table 2:** Microbial safety of food at transport stations

Study	Type of sample	Analytical approach	Microbes reported	Key results	Key conclusion
(A)					
Oguttu *et al*^19^	Ready-to-eat (RTE) chicken	3M Petrifilm plates	*Staphylococcus aureus*	High prevalence of *S. aureus* in the sample was (44%; 90% CI 36.1% to 52.2%), with mean *S. aureus* counts of 103.6 (90% CI 103.3 to 103.9). The likelihood of food poisoning with *S. aureus* from RTE chicken was estimated to be 1.3% (90% CI 0% to 2.7%)	To reduce the levels of concentration of *S. aureus* on the RTE chicken and promote the sale of safer and affordable RTE chicken for the large urban poor population in South Africa, training of RTE chicken vendors on hygiene is still needed.
Mafune *et al*^20^	Unfermented porridge, boiled cabbage and carrots, boiled peanuts, salad, potato chips, traditional mageu, and stewed beef and grilled chicken	Standard microbiological method	*S. aureus*	*S. aureus was <2.4771 log10 cfu/g in all samples and places*. *Except for fried potato chips, microbial contamination was observed in the remaining food samples using the total plate count method*.	Most of the vended foods investigated met the microbiological standard of RTE foods
Mazizi *et al*^23^	Cooked and raw beef, pork, and mutton samples, surface contact plates, and water samples	Biochemical tests according to international standards methods	*S. aureus, Escherichia coli, and Salmonella spp*.	Mean score of raw beef, mutton, and pork were aerobic plate counts (4.8, 3.7 and 2.8 Log (cfu/g)), *S. aureus* (3.3, 3.7 and 2.8 Log cfu/g), and *E. coli* (1.0, 0.6 and 0.3 Log cfu/g) respectively.	The levels of contamination in cooked meat were lower when compared with the standards set by Commission Regulation for determining the microbiological quality of RTE foods.
Tshipamba *et al*^25^	RTE meat	Standard biochemical and molecular methods	*Bacillus thuringienis, Bacillus spp., Bacillus subtilis, Bacillus cereus, Citrobacter spp., Enterococcus faecium, Enterococcus faecalis, Kurthia spp., Lysinibacillus spp., Macrococcus caseolyticus, Planomicrobium glaciei, Planococcus antarcticus, S. aureus, S. equorum, and S. vitulinus*	Overall mean total bacteria in the samples ranged from 4.3 to 6.03 cfu/mL×102 and coliform counts ranged from 1.60 to 1.95×102 cfu/mL Of the 15 microbes identified, S. aureus occurred in all the meat types and the percentage of occurrence was chicken meat (14%), beef head (43%), beef intestine (50%), and wors (sausage) (20%)	Consumers RTE meat are at risk of food borne diseases due to poor hygiene practices of the vendors.
(B)					
Kibret and Tadesse^28^	White lupin	Standard bacteriological techniques, and Kirby-Bauer disk diffusion method for antimicrobial susceptibility test	*E. coli, Salmonella spp, and Shigella spp*.	Prevalence of bacteria total coliform counts were 954.2±385 at the surface and 756.2±447.3 at the core of white lupin. Pathogens isolated were as follows *E. coli* 29 (72.5%), *Salmonella spp.* 23 (57.5%) and *Shigella spp.* 8 (20%). Overall multiple antimicrobial resistances rate was 75%	Contamination of white lupin and a potential health risk to consumers revealed, and the bacteria isolated showed high rates of multiple drug resistance.
Eromo *et al*^29^	Local bread (‘ambasha’ and‘kita’), raw fish, chilli (‘awaze’), avocado, and cooked potato	Standard microbiological techniques	*E. coli, Salmonella spp., and S. aureus*	The microbiological quality in nearly 31% of RTE food samples was beyond the acceptable limits. Total colony counts detected ranged from 1.7×105 to 6.7×106 cfu/g. *E. coli* (29.6%), *Salmonella spp.* (12.7%, and *S. aureus* (9.9%) were the most frequent isolates. All isolates were 100% sensitive to ciprofloxacin, but 89% of *Salmonella spp.* was resistant to chloramphenicol, 14.3% of *S. aureus* was resistant to vancomycin	Considerable rate of contamination in the foods confirmed. The identified foodborne bacteria and antibiotic resistance isolates could pose a public health problem in the study location.
Abakari *et al*^27^	Precut vegetable salads	Standard microbiological methods	*E. coli, Bacillus cereus, Salmonella spp, and Shigella spp*.	*E. coli levels ranged from 0 to 7.56 log10 cfu/g; Bacillus cereus levels ranged from 0 to 7.44 log10 cfu/g; Salmonella spp. ranged from 0 to 4.54 log10 cfu/g, and Shigella spp. ranged from 5.54 log10 cfu/g were detected in 96.7%, 93.3%, 73.3%, and 76.7% of the salads samples, respectively*.	Salads were revealed to be unwholesome for human consumption and could be deleterious to the health of consumers.
(C)					
Flego and Sakyi^4^	RTE foods (ice-kenkey,^15^ cocoa drink,^15^ fufu,^5^ RTE red pepper for kenkey),^5^ salad,^10^ and macaroni^10^)	Standard microbiological methods	*Staphylococci, Bacillus spp., Klebsiella pneumoniae, Aeromonas pneumophila, E. cloacae, S. aureus, E. coli, and P. aeruginosa*	RTE foods were found to be contaminated with enteric bacteria and other potential food poisoning organisms with bacterial counts higher than the acceptable levels (<5.0 log10 CFU/mL). Coagulate negative staphylococci (23.7%), Bacillus species (21.5%), *K. pneumoniae* (18.0%), *Aeromonas pneumophila* (17.7%), *E. cloacae* (6.7%), *S. aureus* (3.7%), *E. coli* (2.2%) and *P. aeruginosa* (2.2%) were the main isolates detected.	Most RTE foods were contaminated with enteric bacteria and other potential food poisoning organisms with bacterial counts higher than the acceptable levels.
Jermini *et al*^33^	Raw foods (ground meat, chicken, and chicken intestine); and processed foods (dried ‘minnows’ and ‘kapenta’)		*Salmonellae Spp., S. aureus, Clostridium peifringens*	Raw foods such as ground meat, chicken, chicken intestine; and processed foods such as dried minnows and kapenta were contaminated by salmonellae or contained high populations of S. aureus in pasteurised milk. High populations (>105) of *S. aureus* were detected from a sample of leftover chicken, more than 107 were detected in leftover rice, and 10 million *C. peifringens* per gram were detected in leftover beef stew sample	Time–temperature exposures during reheating had variable effects in terms of killing the microorganisms that germinated from surviving spores or that reached the foods after cooking.

### Hygiene practices of food handlers/vendors

#### Food preparation

Of the 16 included studies, 8 reported research findings relating to food preparation. Fifty per cent of these eight studies were from South Africa^6 21 23 25^ and the remainder were from Ghana,^26^ Nigeria,^30^ Kenya^31^ and Lesotho.^32^ The studies in South Africa focused on the following: hygiene practices and implications for consumers^21^; food and nutrition knowledge as well as practices related to food preparation,^7^ the effect of hygiene practices and attitudes of meat vendors,^25^ and sources of food contamination.^23^ The study from Ghana investigated how fast food operators washed their hands,^26^ while the studies from Nigeria, Kenya and Lesotho evaluated food safety and sanitary practices^30^; food vendors and hygiene practices; and food safety knowledge, attitudes and practices^31^ of food vendors and consumers’ perceptions.^32^ A summary of the key findings from these studies is presented below (table 3).

**Table 3:** Key reported findings on food preparation

Study	Key findings reported
Kok and Balkaran^21^	Water being used for washing utensils was left unchanged, Piles of dirty pots and dishes was left near the serving areas and RTE foods, and garbage left uncovered with many flies at the site, RTE food was left uncovered, Most of the food handlers were not wearing gloves, hairnets, or aprons
Hill *et al*^22^	85.5% of the vending stalls lacked soap or surface sanitizer, 71% lacked basin for washing, 75% did not have drying cloth, 76.6% of vendors handled food and money concurrently, About 57% left the food uncovered. 39% of the vendors were using their hands to pick up food items, with only 6% wearing gloves, and 29% of vendors had a wet clean sponge/cloth obtainable at the site
Mazizi *et al*^23^	Major sources of food contamination identified were poor hygiene practices of the food vendor, holding area, and the utensils
Tshipamba *et al*^25^	Approximately 90% of RTE meat vendors at the taxi rank exposed their meats to dust and flies, 94% of them handled money while serving food, and Stagnant water found in about 22% of the vending locations at the taxi-rank
McArthur-Floyd *et al*^26^	64% of food vendors washed their hands from elbow to finger and the remainder (36%) washed from their wrist to finger (the WHO recommends handwashing from elbow to fingers), and 62% of the vendors test their meal in the palm while 38% of them test it with a spoon (the best way to test a meal)
Aluko *et al*^30^	Approximately 17% of food vendors washed their hands always after using the toilet, 63% of them rarely kept their fingernails short, and Nearly 4% of them always kept their leftover cooked food in a refrigerator, despites having unstable power supply
Odundo *et al*^31^	Food vendors had poor hygiene practices however, men were observed to have better hygienic practices than women (p<0.05), Hygiene practice of the vendors was found to be significantly associated with training (those trained observe hygiene), and Wearing of jewellery, long and unclean nails, and lack of protective clothing were observed.
Letuka *et al*^32^	Observed that the food handlers operated under unhygienic environment

RTE, ready-to-eat.

#### Knowledge of hygiene practices/food safety precautions

In Ghana, McArthur-Floyd *et al* study^26^ revealed that the majority (94%) of fast food operators knew food safety precautions.^26^ Letuka *et al* study^32^ in Lesotho indicated that 95% of food vendors did not know washing utensils with detergents helps reduce contamination.^32^ The mean knowledge (49%±11) of the food vendors included in the study was considered poor.^32^ About 6% of the consumers that participated in the study chose not to buy food sold at taxi ranks due to food safety issues and hygiene.^32^

#### Occupational health and food safety risk

In South Africa, Qekwana *et al*^24^ evaluated the occupational health and food safety risks associated with the traditional slaughter of goats, and the consumption of such meat.^24^ Approximately 63% of the practitioners were not wearing protective clothing during slaughter, and about 78% of practitioners did not know their health status.^24^ Almost 83% of the practitioners hung up their carcass to facilitate bleeding, flaying and evisceration.^24^ The study further observed that none of the practitioners practiced meat inspection. In Nigeria, Aluko *et al*^30^ study revealed that approximately 62% of the vendors had no formal training, and their medical status was also unknown.^30^

## Discussion

This scoping review mapped evidence on food safety at transport stations in Africa, and revealed a very low number of papers that are published in this area, given many African employees in both formal and informal sectors commute through these transport hubs.^12 13^ An average of one paper per year relating to food safety at transport hubs in Africa as revealed by this review is simply not enough. Nonetheless, the few papers depict an imbalance of research, with most focused on microbial safety,^4 19 20 23 27–29 33^ and few on socioeconomic aspects such as hygiene practices,^6 21 30 31^ and occupational health and food safety risk.^24^ Moreover, this review revealed no study evaluated the storage of food or how the food is transported to the vending site.

As evidence by this review, most of the food sold at transport hubs does not meet the minimum standards and is not safe for consumption due to the presence of several microbes.^4 19 23 25 27 29 33 34^ There are several reasons for this such as poor practices relating to hygiene, storage, preparation, cooking, cleaning and serving.^4 19 20 23 27–29 33^ However, these findings are similar to previous review findings involving markets,^35^ homes and restaurants.^36^ A recent publication by Gizaw^35^ indicated that several studies reported microbial contamination of foods sold in the market, with bacteria and fungi similar to those identified in our review.^35^ Also, a review by the WHO reported that the main factors contributing to foodborne disease outbreaks in homes or restaurants were poor temperature control in preparing, cooking, and storing food.^36^ Although very few papers were found by this review, the evidence is compelling that there should be policy interventions to address issues relating poor hygiene practices, including food storage, preparation, cooking, cleaning and serving by food handlers at transport hubs, not only in South Africa but across Africa.

Similar to a previous scoping review^10^ most of the included papers were published within the last 6 years but, no published study was found in 2015 and 2020. While the reason for the lack of published papers in 2015 might be difficult to determine, the COVID-19 pandemic which resulted in ‘covidisation’ of research might be the reason for the lack of publication in this field of research in 2020. Although we cannot conclude that no primary research has been conducted in these countries focusing on the safety of food sold at transport stations, it suggests a research/publication gap. Food safety research is, perhaps, more relevant now than ever in Africa, since the burden of foodborne diseases is rising annually, resulting in the declaration of food safety as a public health concern by the WHO.^7 8^ Aside from this, most commuters tend to buy ready-to-eat (RTE) food from street food vendors, including those at transport hubs^37 38^; hence, the sale of food at transport stations is rising,^38 39^ particularly in Africa^6^ partly due to an increase in demand for RTE, and the employment opportunities it offers to many individuals who otherwise would not have had any source of income.^10 40^ Even more worrying is the fact that most of the articles included that focused on microbial safety, reported high levels of food contamination with several microorganisms, especially *Salmonella* spp and *E. coli.*^4 19 23 25 27 33 34^ Therefore, more research is needed across African countries to prevent potential negative consequences.

Our study findings have implications for practice and research. For instance, the likelihood of food poisoning with microbes such as *Salmonella* spp, *E. coli., Shigella* spp, *Bacillus* spp, *S. aureus* and several others, revealed by most of the included studies that focused on microbial contamination of food, is alarming. This, if not checked, could further worsen the already high burden of foodborne diseases in a continent that has several of its countries already experiencing many health systems and economic challenges. Aside from this, the majority of individuals who commute through transport hubs, possible will purchase a meal from a transport hub/exchanges site, which may be the only meal^12 13^ of the day and yet the food safety standards are poor.^4 19 20 23 27–29 33^ Thus, if not checked, the excess cases of foodborne diseases from any outbreak will further impact negatively on the already challenged public health systems in Africa. Also, poor people who are exposed to these unsafe foods get an infection, may have to pay more for healthcare, which can further exacerbate their poverty situation. Moreover, people who are already living in extreme poverty who get exposed to foodborne disease may not even make it to the hospital for care and can end up dying at home.^41^

Good hygiene and sanitation practices, such as adequate hand washing, adequate washing and storage of pots and dishes, good waste management, observation of food preparation standards and serving etiquette, among others, have the potential to reduce the risk of food contamination from both biological and non-biological hazards, yet this study reveals fewer studies that focused on hygienic practices. We, therefore, recommend more research to further inform contextualised policy decisions aimed at improving hygiene and sanitation practices by food vendors at transport stations. Also, very relevant to ensuring food safety is the occupational health practices of the vendors. Regular food handling tests and food inspections, conducted by the appropriate local authorities, should be mandatory in all African countries. Food handler tests should seek to ensure that food vendors are fit healthwise to prepare and serve food meant for public consumption. However, our review found limited studies that evaluated occupational health and food safety. Considering that evidence from South Africa and Nigeria suggests about 78% and 62% of food vendors do not know their health status^30 42^ and the increasing number of informal food sellers at various transport exchange sites, future studies are recommended to focus on occupational health and food safety in Africa. The means and manner of storing food, especially leftover RTE food, can either increase or reduce the risk of food contamination, but, again, this scoping review found no study that focused on food storage practices of the vendors at transport stations. Also essential, and yet we did not find any study focusing on it, is the quality of food (nutritious aspects) of the meals sold at transport stations. Eating a well nourishing diet or balanced meals is critical to ensure good health^43–45^; hence, we encourage future primary studies to include the nutritious aspects. Such studies may help streamline guidelines or inform policies to improve the quality of the food sold at transport exchange sites or taxi ranks. Moreover, this review found that the majority (17 out of 18) of the respondents in the included studies were the vendors (mostly women) or food samples taken from the vendors. The perspectives of consumers (buyers) or commuters regarding food safety at transport stations are also very relevant, and we recommend future research to involve them. A comparative study to investigate food safety practices among males and females food vendors at transport stations might be relevant since many males are now getting involved in the business.^6 46 47^

To the best of our knowledge, this study is the first scoping review that systematically mapped literature relating to food safety at transport stations in Africa. A major strength of our study method is that it permits the inclusion of multiple study designs. Also, the choice of this study method permitted us to highlight literature gaps, and made recommendations for future research. Aside from this, we conducted a thorough search in six databases using a comprehensive search strategy which enabled us to capture the most relevant articles to answer the review question. Moreover, two independent reviewers were used to select the studies and perform data extraction processes which helped to prevent selection bias and ensured the reliability and trustworthiness of this study results. Despite this, our scoping review has many limitations. This study included only original study peer reviewed papers, which resulted in the exclusion of one review paper^10^ and one Masters’ dissertation.^48^ We did not also consult the websites of WHO and the Food and Agriculture Organisation websites for possible relevant studies. Furthermore, this study cannot be generalised since the search was limited to African countries only. Although date limitation was removed, we limited the publication language to English only, which perhaps eliminated relevant articles published in other languages. Despite these limitations, this study has provided essential evidence relating to food safety at transport stations and has shown literature gaps to guide future research.

## Conclusion

Based on this scoping review’s eligibility criteria, our study results suggest there is limited research focusing on food safety at transport stations in Africa. Most of the existing published studies are focused on microbial safety of food, and very few/none on other aspects such as hygiene practices, food storage, occupational health and food safety, and nutrition. Hence, we recommend more primary research involving community members and policy-makers in these areas going forward alongside improving access to clean water/handwashing facilities, and undertaking structural changes to facilitate behaviours and monitoring for unintended consequences such as livelihoods of vulnerable populations.

## Supplementary Material

Reviewer comments

Author's manuscript

## Data Availability

Data are available upon reasonable request. Data sharing not applicable as no datasets generated and/or analysed for this study. All data relevant to the study are included in the article or uploaded as supplementary information.

## References

[1] The Lancet Gastroenterology Hepatology. Food safety really is everyone's business. Lancet Gastroenterol Hepatol 2019;4:571. 10.1016/S2468-1253(19)30202-X 31292067

[2] World Health Organization. Estimating the burden of foodborne diseases Geneva: World Health organization, 2015. Available: https://www.who.int/activities/estimating-the-burden-of-foodborne-diseases [Accessed 25 Oct 2019]. 10.2471/BLT.14.148056 PMC4581658 26478611

[3] World Health Organization. Food safety Geneva: World Health organization, 2016. Available: https://www.afro.who.int/health-topics/food-safety [Accessed 17 Dec 2020].

[4] Feglo P, Sakyi K. Bacterial contamination of street vending food in Kumasi, Ghana. J Medical Biomed Sci 2012;1:1–8.

[5] Organization WH. Food safety: what you should know Geneva: World Health organization, 2015. Available: http://www.searo.who.int/entity/world_health_day/2015/whd-what-you-should-know/en/ [Accessed 25 Oct 2019].

[6] Hill J, Mchiza Z, Puoane T, et al. The development of an evidence-based street food vending model within a socioecological framework: a guide for African countries. PLoS One 2019;14:e0223535. 10.1371/journal.pone.0223535 31639126 PMC6804966

[7] World Health Organization. Five keys to safer food manual Geneva: World Health Organization, 2006. Available: https://www.who.int/foodsafety/publications/consumer/manual_keys.pdf [Accessed 25 Oct 2019].

[8] Lamin-Boima P. Knowledge, attitude and practice of street food vendors in selected schools within bo City southern Sierra Leone. Int J Sci Res 2017;6:254–9.

[9] Lues JFR, Rasephei MR, Venter P, et al. Assessing food safety and associated food handling practices in street food vending. Int J Environ Health Res 2006;16:319–28. 10.1080/09603120600869141 16990173

[10] Abrahale K, Sousa S, Albuquerque G, et al. Street food research worldwide: a scoping review. J Hum Nutr Diet 2019;32:152–74. 10.1111/jhn.12604 30311276

[11] Liu Z, Zhang G, Zhang X. Urban street foods in Shijiazhuang city, China: current status, safety practices and risk mitigating strategies. Food Control 2014;41:212–8. 10.1016/j.foodcont.2014.01.027

[12] Food and Agriculture Organization. Selling street and snack foods Rome: food and agriculture organization, 2011. Available: http://www.fao.org/3/a-i2474e.pdf [Accessed 25 Oct 2019].

[13] Gelormini M, Damasceno A, Lopes SA, et al. Street food environment in Maputo (STOOD MAP): a cross-sectional study in Mozambique. JMIR Res Protoc 2015;4:e98. 10.2196/resprot.4096 26245231 PMC4705368

[14] Arksey H, O'Malley L. Scoping studies: towards a methodological framework. Int J Soc Res Methodol 2005;8:19–32. 10.1080/1364557032000119616

[15] Levac D, Colquhoun H, O'Brien KK. Scoping studies: advancing the methodology. Implement Sci 2010;5:69. 10.1186/1748-5908-5-69 20854677 PMC2954944

[16] Ncama BP, Kuupiel D, Duma SE, et al. Mapping evidence of food safety at transport stations in Africa: a scoping review protocol. BMJ Open 2020;10:e035879-e. 10.1136/bmjopen-2019-035879 PMC7418661 32784254

[17] Tricco AC, Lillie E, Zarin W, et al. PRISMA extension for scoping reviews (PRISMA-ScR): checklist and explanation. Ann Intern Med 2018;169:467–73. 10.7326/M18-0850 30178033

[18] Liberati A, Altman DG, Tetzlaff J, et al. The PRISMA statement for reporting systematic reviews and meta-analyses of studies that evaluate health care interventions: explanation and elaboration. J Clin Epidemiol 2009;62:1000097:e1–34. 10.1016/j.jclinepi.2009.06.006 19631507

[19] Oguttu JW, McCrindle CME, Makita K, et al. Investigation of the food value chain of ready-to-eat chicken and the associated risk for staphylococcal food poisoning in Tshwane Metropole, South Africa. Food Control 2014;45:87–94. 10.1016/j.foodcont.2014.04.026

[20] Mafune TS, Takalani TK, Anyasi TA, et al. Microbial safety of street Vended foods sold in Thohoyandou, South Africa. J Hum Ecol 2016;53:205–12. 10.1080/09709274.2016.11906973

[21] Kok R, Balkaran R. Street food vending and hygiene practices and implications for consumers. JEBS 2014;6:188–93. 10.22610/jebs.v6i3.482

[22] Hill J, Mchiza Z, Puoane T, et al. Food sold by street-food vendors in Cape town and surrounding areas: a focus on food and nutrition knowledge as well as practices related to food preparation of street-food vendors. J Hunger Environ Nutr 2019;14:401–15. 10.1080/19320248.2018.1434104

[23] Mazizi BE, Muchenje V, Makepe M, et al. Assessment of Aerobic Plate Counts, Staphylococcus aureus, Escherichia coli and Salmonella in Meat Sold by Street Vendors in the Eastern Cape Province, South Africa. J Food Nutr Res 2017;5:436–42. 10.12691/jfnr-5-6-11

[24] Qekwana DN, McCrindle CME, Oguttu JW, et al. Assessment of the occupational health and food safety risks associated with the traditional slaughter and consumption of goats in Gauteng, South Africa. Int J Environ Res Public Health 2017;14:420. 10.3390/ijerph14040420 PMC5409621 28420084

[25] Tshipamba M, Lubanza N, Adetunji M. Evaluation of the effect of hygiene practices and attitudes on the microbial quality of street Vended meats sold in Johannesburg, South-Africa. J Food Prot 2018;3:2476–059.

[26] McArthur-Floyd M, Commey V, Boakye NAB. Evaluation of food safety among fast food operators in Madina, Accra. Evaluation 2016;54.

[27] Abakari G, Cobbina SJ, Yeleliere E. Microbial quality of ready-to-eat vegetable salads vended in the central business district of Tamale, Ghana. Int J Food Contam 2018;5. 10.1186/s40550-018-0065-2

[28] Kibret M, Tadesse M. The bacteriological safety and antimicrobial susceptibility of bacteria isolated from street-vended white lupin (Lupinus albus) in Bahir Dar, Ethiopia. Ethiop J Health Sci 2013;23:19–26. 23559834 PMC3613811

[29] Eromo T, Tassew H, Daka D, et al. Bacteriological quality of street foods and antimicrobial resistance of isolates in Hawassa, Ethiopia. Ethiop J Health Sci 2016;26:533–42. 10.4314/ejhs.v26i6.5 28450768 PMC5389072

[30] Aluko OO, Ojeremi TT, Olaleke DA, et al. Evaluation of food safety and sanitary practices among food vendors at CAR Parks in Ile Ife, southwestern Nigeria. Food Control 2014;40:165–71. 10.1016/j.foodcont.2013.11.049

[31] Odundo A, Okemo P, Chege P. An assessment of food safety practices among street vendors in Mombasa, Kenya. Int J Environ Res Public Health 2018;8:235–43.

[32] Letuka P, Nkhebenyane J, Thekisoe O. Assessment of Food Safety Knowledge, Attitudes and Practices among Street Food Vendors and Consumers’ Perceptions of Street Food Vending in Maseru Lesotho, 2019.

[33] Jermini M, Bryan FL, Schmitt R, et al. Hazards and critical control points of food Vending operations in a City in Zambia. J Food Prot 1997;60:288–99. 10.4315/0362-028X-60.3.288 31195492

[34] Kibret M, Tadesse M, MJEjohs T. The bacteriological safety and antimicrobial susceptibility of bacteria isolated from street-vended white lupin (Lupinus albus) in Bahir Dar, Ethiopia. Ethiop J Health Sci 2013;23:19–26. 23559834 PMC3613811

[35] Gizaw Z. Public health risks related to food safety issues in the food market: a systematic literature review. Environ Health Prev Med 2019;24:68. 10.1186/s12199-019-0825-5 31785611 PMC6885314

[36] Todd EC. Epidemiology of foodborne diseases: a worldwide review. World Health Stat Q 1997;50:30–50. 9282385

[37] Bellia C, Pilato M, Seraphin H. Street food and food safety: a driver for tourism? Calitatea 2016;17:20.

[38] Mosupye FM, von Holy A. Microbiological hazard identification and exposure assessment of street food vending in Johannesburg, South Africa. Int J Food Microbiol 2000;61:137–45. 10.1016/S0168-1605(00)00264-6 11078164

[39] Mosupye FM, von Holy A. Microbiological quality and safety of ready-to-eat street-vended foods in Johannesburg, South Africa. J Food Prot 1999;62:1278–84. 10.4315/0362-028X-62.11.1278 10571317

[40] Muzaffar AT, Huq I, Mallik BA. Entrepreneurs of the streets: an analytical work on the street food vendors of Dhaka City. IJBM 2009;4:10.5539/ijbm.v4n2p80:80–8. 10.5539/ijbm.v4n2p80

[41] World Health Organisation. Universal health coverage Geneva, 2020. Available: https://www.who.int/health-topics/universal-health-coverage#tab=tab_1 [Accessed 09 Apr 2021].

[42] Qekwana D, McCrindle C, Oguttu J, et al. Assessment of the occupational health and food safety risks associated with the traditional slaughter and consumption of goats in Gauteng, South Africa. Int J Environ Res Public Health 2017;14:420. 10.3390/ijerph14040420 PMC5409621 28420084

[43] Melanson KJ. Nutrition review: lifestyle approaches to promoting healthy eating for children. Am J Lifestyle Med 2008;2:10.1177/1559827607309217:26–9. 10.1177/1559827607309217

[44] Carrier N, Villalon L, Lengyel C, et al. Diet quality is associated with malnutrition and low calf circumference in Canadian long-term care residents. BMC Nutr 2019;5:57. 10.1186/s40795-019-0314-7 32153970 PMC7050926

[45] Freeland-Graves JH, Nitzke S, Academy of Nutrition and Dietetics. Position of the Academy of nutrition and dietetics: total diet approach to healthy eating. J Acad Nutr Diet 2013;113:10.1016/j.jand.2012.12.013:307–17. 10.1016/j.jand.2012.12.013 23351634

[46] Okojie PW, Isah EC. Sanitary conditions of food vending sites and food handling practices of street food vendors in Benin City, Nigeria: implication for food hygiene and safety. J Environ Public Health 2014;2014:1–6. 10.1155/2014/701316 PMC4166661 25258630

[47] Alves da Silva S, Cardoso RdeCV, Góes José Ângelo Wenceslau, et al. Street food on the coast of Salvador, Bahia, Brazil: a study from the socioeconomic and food safety perspectives. Food Control 2014;40:78–84. 10.1016/j.foodcont.2013.11.022

[48] Acheampong BE. Assessment of food hygiene practices by street food vendors and microbial quality of selected foods sold. A study at Dunkwa-On-Offin, upper Denkyira East Municipality of the central region, 2015.

