# Supplementary material for: Scoping review of food safety at transport stations in Africa
Source: BMJ Open. 2021 Nov 25;11(11):e053856. doi: 10.1136/bmjopen-2021-053856 (PMC8627411; doi:10.1136/bmjopen-2021-053856)
Supplement: Supplementary data [file bmjopen-2021-053856supp001.pdf]

Supplementary file 1: Electronic databases search

**Search date**	**Database**	**Keywords**	**Search results**	**Number eligible**
05/10/2020	EBSCOhost (Academic search complete, CINAHL with Full-text, and Health Source	SU food safety AND SU (food preparation or meal preparation or cooking) OR SU food handling OR SU food storage OR hygiene practices AND (food trading or food selling or food vending or street food) AND (transport station or taxi rank or bus station or transport exchange sites or car park or lorry park) AND africa	2,549	14
07/10/2020	PubMed	"food safety"[MeSH Terms] OR ("food"[All Fields] AND "safety"[All Fields]) OR "food safety"[All Fields]) OR ("food supply"[MeSH Terms] OR ("food"[All Fields] AND "supply"[All Fields]) OR "food supply"[All Fields] OR ("food"[All Fields] AND "security"[All Fields]) OR "food security"[All Fields]) AND (("food"[MeSH Terms] OR "food"[All Fields]) AND vending[All Fields]) OR (("food"[MeSH Terms] OR "food"[All Fields]) AND trading[All Fields]) AND streets[All Fields] OR (("motor vehicles"[MeSH Terms] OR ("motor"[All Fields] AND "vehicles"[All Fields]) OR "motor vehicles"[All Fields] OR "lorry"[All Fields]) AND parks[All Fields]) OR (("motor vehicles"[MeSH Terms] OR ("motor"[All Fields] AND "vehicles"[All Fields]) OR "motor vehicles"[All Fields] OR "lorry"[All Fields]) AND station[All Fields]) OR (taxi[All Fields] AND ranks[All Fields]) AND ("algeria"[MeSH Terms] OR "algeria"[All Fields]) OR ("angola"[MeSH Terms] OR "angola"[All Fields]) OR ("benin"[MeSH Terms] OR "benin"[All Fields]) OR ("botswana"[MeSH Terms] OR "botswana"[All Fields]) OR ("burkina faso"[MeSH Terms] OR ("burkina"[All Fields] AND "faso"[All Fields]) OR "burkina faso"[All Fields]) OR ("burundi"[MeSH Terms] OR "burundi"[All Fields]) OR ("cameroon"[MeSH Terms] OR "cameroon"[All Fields]) OR ("cabo verde"[MeSH Terms] OR ("cabo"[All Fields] AND "verde"[All Fields]) OR "cabo verde"[All Fields] OR ("cape"[All Fields] AND "verde"[All Fields]) OR "cape verde"[All Fields]) OR ("central african republic"[MeSH Terms]	2,834	33

		OR ("central"[All Fields] AND "african"[All Fields] AND "republic"[All Fields]) OR "central african republic"[All Fields]) OR ("chad"[MeSH Terms] OR "chad"[All Fields]) OR ("democratic republic of the congo"[MeSH Terms] OR ("democratic"[All Fields] AND "republic"[All Fields] AND "congo"[All Fields]) OR "democratic republic of the congo"[All Fields]) OR ("congo"[MeSH Terms] OR "congo"[All Fields] OR ("republic"[All Fields] AND "congo"[All Fields]) OR "republic of the congo"[All Fields]) OR ("djibouti"[MeSH Terms] OR "djibouti"[All Fields]) OR ("egypt"[MeSH Terms] OR "egypt"[All Fields]) OR ("equatorial guinea"[MeSH Terms] OR ("equatorial"[All Fields] AND "guinea"[All Fields]) OR "equatorial guinea"[All Fields]) OR ("eritrea"[MeSH Terms] OR "eritrea"[All Fields]) OR ("ethiopia"[MeSH Terms] OR "ethiopia"[All Fields]) OR ("gabon"[MeSH Terms] OR "gabon"[All Fields]) OR ("gambia"[MeSH Terms] OR "gambia"[All Fields]) OR ("ghana"[MeSH Terms] OR "ghana"[All Fields]) OR ("guinea"[MeSH Terms] OR "guinea"[All Fields]) OR ("guinea-bissau"[MeSH Terms] OR "guinea-bissau"[All Fields] OR ("guinea"[All Fields] AND "bissau"[All Fields]) OR "guinea bissau"[All Fields]) OR ("cote d'ivoire"[MeSH Terms] OR ("cote"[All Fields] AND "d'ivoire"[All Fields]) OR "cote d'ivoire"[All Fields] OR ("ivory"[All Fields] AND "coast"[All Fields]) OR "ivory coast"[All Fields]) OR ("kenya"[MeSH Terms] OR "kenya"[All Fields]) OR ("lesotho"[MeSH Terms] OR "lesotho"[All Fields]) OR ("liberia"[MeSH Terms] OR "liberia"[All Fields]) OR ("libya"[MeSH Terms] OR "libya"[All Fields]) OR ("madagascar"[MeSH Terms] OR "madagascar"[All Fields]) OR ("malawi"[MeSH Terms] OR "malawi"[All Fields]) OR ("mali"[MeSH Terms] OR "mali"[All Fields]) OR ("mauritania"[MeSH Terms] OR "mauritania"[All Fields]) OR ("mauritius"[MeSH Terms] OR "mauritius"[All Fields]) OR ("morocco"[MeSH Terms] OR "morocco"[All Fields]) OR ("mozambique"[MeSH Terms] OR "mozambique"[All Fields]) OR ("namibia"[MeSH Terms] OR "namibia"[All Fields]) OR ("niger"[MeSH Terms] OR "niger"[All Fields]) OR ("nigeria"[MeSH Terms] OR "nigeria"[All Fields]) OR ("rwanda"[MeSH Terms] OR		

		"rwanda"[All Fields]) OR ("sao tome and principe"[MeSH Terms] OR ("sao"[All Fields] AND "tome"[All Fields] AND "principe"[All Fields]) OR "sao tome and principe"[All Fields]) OR ("senegal"[MeSH Terms] OR "senegal"[All Fields]) OR ("seychelles"[MeSH Terms] OR "seychelles"[All Fields]) OR ("sierra leone"[MeSH Terms] OR ("sierra"[All Fields] AND "leone"[All Fields]) OR "sierra leone"[All Fields]) OR ("somalia"[MeSH Terms] OR "somalia"[All Fields]) OR (("south africa"[MeSH Terms] OR ("south"[All Fields] AND "africa"[All Fields]) OR "south africa"[All Fields]) AND South[All Fields]) OR ("sudan"[MeSH Terms] OR "sudan"[All Fields]) OR ("sudan"[MeSH Terms] OR "sudan"[All Fields]) OR ("eswatini"[MeSH Terms] OR "eswatini"[All Fields] OR "swaziland"[All Fields]) OR ("tanzania"[MeSH Terms] OR "tanzania"[All Fields]) OR ("togo"[MeSH Terms] OR "togo"[All Fields]) OR ("tunisia"[MeSH Terms] OR "tunisia"[All Fields]) OR ("uganda"[MeSH Terms] OR "uganda"[All Fields]) OR ("zambia"[MeSH Terms] OR "zambia"[All Fields]) OR ("zimbabwe"[MeSH Terms] OR "zimbabwe"[All Fields]) Filters: Free full text, Comparative Study, Observational Study, Randomized Controlled Trial, Humans, English, MEDLINE		
09/10/2020	Web of Science	(food safety AND food preparation OR meal preparation OR cooking OR food handling OR food storage OR hygiene practices AND food trading OR food selling OR food vending or street food AND transport station OR taxi rank OR bus station OR transport exchange sites OR car park or lorry park AND africa)Refined by: Open Access: (OPEN ACCESS) AND DOCUMENT TYPES: (ARTICLE) AND DOCUMENT TYPES: (ARTICLE) AND WEB OF SCIENCE CATEGORIES: (FOOD SCIENCE TECHNOLOGY OR PUBLIC ENVIRONMENTAL OCCUPATIONAL HEALTH) AND WEB OF SCIENCE CATEGORIES: (FOOD SCIENCE TECHNOLOGY OR PUBLIC ENVIRONMENTAL OCCUPATIONAL HEALTH OR NUTRITION DIETETICS)Timespan: All years. Indexes: SCI-EXPANDED, SSCI, A&HCI, CPCI-S, CPCI-SSH, BKCI-S, BKCI-SSH, ESCI.	8,263	36
18/10/2020	SCOPUS	food safety AND food preparation OR meal preparation OR cooking OR food handling OR food storage OR hygiene practices AND food trading OR	116	7

		food selling OR food vending or street food AND transport station OR taxi rank OR bus station OR transport exchange sites OR car park or lorry park AND africa		
25/10/2020	Google Scholar	food safety AND food preparation OR meal preparation OR cooking OR food handling OR food storage OR hygiene practices AND food trading OR food selling OR food vending or street food AND transport station OR taxi rank OR bus station OR transport exchange sites OR car park or lorry park AND africa	9,820	56
			23,582	146
**Duplicates**				**30**
