# Supplementary material for: Scoping review of food safety at transport stations in Africa
Source: BMJ Open. 2021 Nov 25;11(11):e053856. doi: 10.1136/bmjopen-2021-053856 (PMC8627411; doi:10.1136/bmjopen-2021-053856)
Supplement: Author's manuscript [file bmjopen-2021-053856.draft_revisions.pdf]

BMJ Open is committed to open peer review. As part of this commitment we make the peer review history of every article we publish publicly available.

When an article is published we post the peer reviewers' comments and the authors' responses online. We also post the versions of the paper that were used during peer review. These are the versions that the peer review comments apply to.

The versions of the paper that follow are the versions that were submitted during the peer review process. They are not the versions of record or the final published versions. They should not be cited or distributed as the published version of this manuscript.

BMJ Open is an open access journal and the full, final, typeset and author-corrected version of record of the manuscript is available on our site with no access controls, subscription charges or pay-per-view fees (http://bmjopen.bmj.com).

If you have any questions on BMJ Open's open peer review process please email info.bmjopen@bmj.com

BMJ Open

A Scoping Review of Food Safety at Transport stations in Africa

Journal:	*BMJ Open*
Manuscript ID	bmjopen-2021-053856
Article Type:	Original research
Date Submitted by the Author:	25-May-2021

Complete List of Authors:	Ncama, Busisiwe; College of Health Sciences University of KwaZulu-Natal<br>Kuupiel , Desmond ; College of Health Sciences University of KwaZulu-Natal<br>Duma, Sinegugu; University of KwaZulu-Natal College of Health Sciences<br>Mchunu , Gugu ; University of KwaZulu-Natal College of Health Sciences<br>Guga , Phindile ; University of KwaZulu-Natal College of Health Sciences<br>Slotow, Rob; University of KwaZulu-Natal

Keywords:	Public health < INFECTIOUS DISEASES, PUBLIC HEALTH, Health & safety < HEALTH SERVICES ADMINISTRATION & MANAGEMENT

SCHOLARONE™
Manuscripts

I, the Submitting Author has the right to grant and does grant on behalf of all authors of the Work (as defined in the below author licence), an exclusive licence and/or a non-exclusive licence for contributions from authors who are: i) UK Crown employees; ii) where BMJ has agreed a CC-BY licence shall apply, and/or iii) in accordance with the terms applicable for US Federal Government officers or employees acting as part of their official duties; on a worldwide, perpetual, irrevocable, royalty-free basis to BMJ Publishing Group Ltd ("BMJ") its licensees and where the relevant Journal is co-owned by BMJ to the co-owners of the Journal, to publish the Work in this journal and any other BMJ products and to exploit all rights, as set out in our licence.

The Submitting Author accepts and understands that any supply made under these terms is made by BMJ to the Submitting Author unless you are acting as an employee on behalf of your employer or a postgraduate student of an affiliated institution which is paying any applicable article publishing charge ("APC") for Open Access articles. Where the Submitting Author wishes to make the Work available on an Open Access basis (and intends to pay the relevant APC), the terms of reuse of such Open Access shall be governed by a Creative Commons licence – details of these licences and which Creative Commons licence will apply to this Work are set out in our licence referred to above.

Other than as permitted in any relevant BMJ Author's Self Archiving Policies, I confirm this Work has not been accepted for publication elsewhere, is not being considered for publication elsewhere and does not duplicate material already published. I confirm all authors consent to publication of this Work and authorise the granting of this licence.

For peer review only

A Scoping Review of Food Safety at Transport stations in Africa

Busisiwe P. Ncama[1], Desmond Kuupiel[1,2], Sinegugu E. Duma[1], Gugu Mchunu[1], Phindile Guga[1], Rob Slotow[3]

[1]School of Nursing and Public Health, College of Health Sciences, University of KwaZulu-Natal, Durban, South Africa

[2]Research for Sustainable Development Consult, Sunyani, Ghana

[3]School of Life Sciences, College of Agriculture, Engineering and Science, University of Kwazulu-Natal, Pietermaritzburg, South Africa.

Corresponding Author: Desmond Kuupiel (PhD)

Address: 2nd Floor George Campbell Building, Department of Public Health Medicine, School of Nursing and Public Health, University of KwaZulu-Natal, Durban, 4001, South Africa

Email address: desmondkuupiel98@hotmail.com / KuupielD@ukzn.ac.za

Tel: +27735568200/ +233550972968

Email addresses of co-authors

Prof. Busisiwe P. Ncama (Ncamab@ukzn.ac.za)

Prof. Sinegugu E. Duma (DumaS1@ukzn.ac.za)

Prof. Gugu Mchunu (Mchunug@ukzn.ac.za)

Ms. Phindile Guga (phindile.guga@gmail.com)

Prof. Rob Slotow (Slotow@ukzn.ac.za)

For peer review only

Abstract

Background: The World Health Organisation (WHO) has declared food safety as a public health concern. Transport hubs such as taxi ranks, bus stations, and other transport exchange sites are major food trading/purchasing sites, particularly in Africa. Research evidence is needed to improve food safety policies and ensure consumption of safe food, owing to the increasing burden of foodborne diseases, particularly in the WHO Africa Region. Therefore, we systematically mapped and described research evidence on food safety at transport stations in Africa.

Methods: Guided by the Arksey and O'Malley framework, we searched for original research articles in PubMed, Web of Science, and EBSCOhost (Academic search complete, CINAHL with Full-text, and Health Source), SCOPUS, and Google Scholar from their inception to October 2020. We included studies that focused on food safety, involved transport stations, involved African countries, and were published in English. Two investigators independently screened the articles at the abstract and full-text stages in parallel, guided by the eligibility criteria, and then extracted all relevant data. Thematic analysis was used to organise the data into themes and sub-themes, and a narrative summary of the findings is presented.

Results: Of the total 23,852 articles obtained from the database searches, 18 studies published in six countries met the inclusion criteria. These 18 studies were published between 1997 and 2019, with the most (5) in 2014. Of the 18 studies, 50% (9) were conducted in South Africa, three studies in Ghana, two in Ethiopia, and one study each in Nigeria, Kenya, Lesotho, and Zambia. Most (44.4%) of the included studies focused on microbial safety of food; few studies (22.2%) focused on hygienic practices, and one study investigated the perspective of consumers or buyers. Most of the included studies reported that food sold at transport hubs failed to meet the minimum standard. The microbes detected in the foods were *Salmonella* spp, *E. coli*, *Shigella* spp, *Bacillus* sp, *Staphylococcus Aureu*, which resulted mainly from poor hygiene practices.

Conclusion: There is limited research that focused on food safety at transport stations in Africa, especially on aspects such as hygiene practices, food storage, occupational health and food safety, and nutritious aspects. Therefore, we recommend more research in these areas, using various primary study designs, to inform and improve food safety policies and practices for transport stations in African countries alongside improving access to clean water/hand washing facilities, and undertaking of structural changes to facilitate behaviours and monitoring for unintended consequences such as livelihoods of vulnerable populations.

For peer review only - http://bmjopen.bmj.com/site/about/guidelines.xhtml

Keywords: Food safety, Transport stations, Taxi ranks, Automobile station, Bus stops, Africa

Article summary

Strengths and limitations of this study

- To the best of our knowledge this is the first scoping review to systematically explore literature and describe research evidence on food safety at transport stations as well as identify research gaps for future studies in Africa.
- This scoping review evidence sources were searched using systematic approach, and duplicate screening.
- This review is limited to Africa as well as English language publications.

For peer review only - http://bmjopen.bmj.com/site/about/guidelines.xhtml

Background

The World Health Organisation (WHO) estimates that more than 600 million people fall sick (almost 1 in 10 people) with foodborne diseases annually, of which nearly 420 000 people die, and about 33 million years of healthy lives are lost every year worldwide [1, 2]. The burden of foodborne diseases is estimated to be highest in the WHO African and South-East Asia Regions, mainly occurring among vulnerable populations such as infants, young children, pregnant women, older people, poor people, and individuals with underlying illnesses [3]. Food contamination mostly results throughout the food supply chain (from the procedures used in processing the foods, inadequate storage temperatures, unhygienic practices by food handlers, poor sanitation at place of cooking/vending areas, poor waste management, and inadequate treatment of leftovers) [4].

Unsafe food has negative implications on health systems, and affects the development and national economies of countries, as well as trade [3]. Therefore, eating unsafe foods poses a significant public health threat. To avert the consequences of unsafe food on health systems, and to sustain national economies, development, trade, and tourism [5, 6], the WHO in 2006 declared food safety as a global public health concern [7, 8]. Food safety consists of food preparation, handling, storage, and hygienic practices aimed to prevent food contamination by microbial, chemical, and physical hazards in the food production chain [5]. To reduce the incidence of food-related diseases, particularly in high burden regions, the observations of food safety measures/precautions at all levels of the food processing chain, including the places where food is prepared and sold, are critical [9, 10].

Like other WHO Regions, especially in low-and-middle-income countries, food trading in the Africa Region takes place at several formal and informal places, such as in the markets, restaurants, streets, open spaces in academic institutions, and transport stations (taxi ranks, bus stations, lorry parks), and other transport exchange sites. Food vending at public spaces serves as a source of livelihood [6, 10, 11], and more than two billion people eat food sold at various vending locations. including transportations stations on daily basis globally [12, 13]. To this end, evidence is essential to inform in-country policies/guidelines, and further research, to ensure that food prepared and sold at transport stations promotes livelihoods, nutrition, food safety, and environmentally sustainable practices. This scoping review systematically mapped literature focused on food safety at transport stations in Africa, to provide research evidence and gaps.

For peer review only - http://bmjopen.bmj.com/site/about/guidelines.xhtml

Methods

The Arksey & O'Malley framework (research question identification; identifying relevant studies; selection of study; data charting, collating, and summarising and reporting the findings [14, 15]) was employed to scope and synthesise literature to answer the question - what evidence exists on food safety at transport stations in Africa? This review's study protocol was developed *a priori* [16]. This study included published peer-reviewed articles that reported findings from any African country/countries, focused on food safety, and involved transport stations. However, this study was limited to English publications (due to lack of expertise in other international languages), and primary study designs [16]. We followed the Preferred Reporting Items for Systematic and Meta-analysis (PRISMA) extension for Scoping Reviews checklist to report this study [17].

Identify relevant studies

We searched for primary research articles relating to food safety at transport stations in PubMed, Web of Science, and EBSCOhost (Academic search complete, CINAHL with Full-text, and Health Source), SCOPUS, and Google Scholar from their inception to October 2020. To enable the capturing of all relevant articles, a comprehensive search strategy (developed in consultation with an expert librarian) consisting of keywords, Boolean terms (AND/OR), and Medical Subject Heading terms, was used for the electronic database search (Supplementary file 1). Syntax was modified appropriately where needed. Filters such as date and study design were not applied during the literature search in the databases. DK and PG independently conducted the database search and title screening, and imported all potentially eligible articles onto an EndNote Library. The reference lists of all included articles were also screened for potentially relevant articles using the same approach.

Selection of articles and edibility criteria

Prior to the abstract screening, the 'find duplicates' function in EndNote was used to find all duplicate articles, and they were removed from the library. A screening form was developed in Google forms, using this study's eligibility criteria, for the abstract and full text screening phases. Two reviewers independently screened the abstracts as well as the full text articles. Discrepancies that arose during the abstract stage were resolved by discussion among the review team until a consensus was reached. At the full text screening phase, discrepancies were resolved by a third reviewer. All the additional articles identified from the reference list of the included articles equally underwent full text assessment. The PRISMA flow diagram was

For peer review only - http://bmjopen.bmj.com/site/about/guidelines.xhtml

employed to account for all the articles involved [18].

Charting the data

A data extraction form was designed consisting of the following: Author(s) and publication details, country of study, study design, study setting, study population, sample size, sex, study findings, and recommendations. To ensure consistency and reliability, two reviewers piloted the data extraction sheet using a random sample of three included studies. The pilot testing of the form also enabled the review team to discuss discrepancies, and to revise the data extraction form prior to its final usage. Subsequently, two reviewers conducted the data extraction for the remaining 15 included studies using both inductive and deductive approaches. The review team resolved all discrepancies at this stage through discussion.

Collating, summarizing, and reporting the results

This study subsequently employed thematic analysis, and collated all the emerging themes and sub-themes relating to food safety. A summary of the findings from the included studies is presented narratively.

Results

Of the 23,852 articles obtained from the database searches (see Figure 1 flow diagram), 146 articles met the eligibility criteria at the title screening stage. Using EndNote "Find Duplicates" function, 30 duplicates were found and removed before abstract screening was conducted. Subsequently, 83 articles were removed at the abstract screening, and 18 at full text (16 of these did not include transport stations/taxi ranks/bus stations, but did involve sale from market centres, public places, chop bars, mini restaurants, major streets, and sidewalks, and were excluded). Finally, 15 studies were included, and, from a manual search of their reference lists, a further three articles were added, giving a total of 18 articles for further analysis.

Characteristics of the included studies

Table 1 presents a summary of the characteristics of the included studies. Of the 18 included studies, nine (50%) were conducted in South Africa [19-26], three (16.7%) in Ghana [4, 27, 28], two (11.1%) in Ethiopia [29, 30], and one (5.6%) each in Nigeria [31], Kenya [32], Lesotho [33], and Zambia [34]. Most of the studies were published in the last six years, however, no published study was found in 2015 and 2020 (Figure 2). Seventeen (94.4%) of the included

For peer review only - http://bmjopen.bmj.com/site/about/guidelines.xhtml

studies were cross-sectional studies, and one (5.6%) was a mixed-method study. Of the 18 included studies, 44.4% reported on microbial safety of food [4, 19, 22, 23, 28-30, 34], and 22.2% reported hygiene practices of food handlers/vendors [6, 21, 31, 32]. One included study each reported on the following: food safety risk communication [25]; knowledge of hygiene practice [27]; knowledge of food safety measures [26]; occupational health and food safety risk [20]; hygiene practices of food handlers/vendors and microbial safety [35]; and knowledge of food safety measures and hygiene practice by food handlers/vendors [33].

Findings from the included studies

Microbial safety of food

Of the nine included studies that reported findings on microbial safety of food, 44.4% were conducted in South Africa [22, 23, 35, 36], 22.2% each in Ghana [4, 28] and Ethiopia [29, 30], and the last 11.1% in Zambia [34]. Seven of the eight studies reported unacceptable level of microbes in the food [4, 19, 22, 23, 28-30, 34]. Table 2 presents a summary of the key findings as well as the sample type, analytic approach, and the microbes reported.

Hygiene practices of food handlers/vendors

Food preparation

Of the 18 included studies, eight reported research finding relating to food preparation. Fifty percent of these eight studies were from South Africa [6, 21, 23, 35], and the remainder were from Ghana [27], Nigeria [31], Kenya[32], and Lesotho [33]. The studies in South Africa focused on the following: hygiene practices and implications for consumers [21]; food and nutrition knowledge as well as practices related to food preparation [6], the effect of hygiene practices and attitudes of meat vendors [35], and sources of food contamination [23]. The study from Ghana investigated how fast food operators washed their hands [27], whilst the studies from Nigeria, Kenya, and Lesotho evaluated food safety and sanitary practices [31]; food vendors and hygiene practices [32]; and food safety knowledge, attitudes and practices of food vendors and consumers' perceptions [33]. A summary of the key findings from these studies are presented below (Table 3).

Knowledge of hygiene practices/food safety precautions

For peer review only - http://bmjopen.bmj.com/site/about/guidelines.xhtml

In South Africa, Qekwana et. al. [26] survey involving traditional goat slaughters revealed that traditional and ritual slaughter includes preslaughter activities aimed to mitigate risk of slaughtering animals that are not fit for human consumption, yet none of the study respondents was aware of the need for a health declaration for slaughter stock [26]. Few (21%) slaughter practitioners perform a prepurchase inspection of stock to ascertain their health status [26]. McArthur-Floyd et. al. [27] study in Ghana, revealed that the majority (94%) of fast food operators knew food safety precautions [27]. Letuka et. al. [33] study in Lesotho, indicated that 95% of food vendors had incorrect knowledge that washing utensils with detergent leave them free of contamination [33]. The mean knowledge (49%±11) of the food vendors included in the study was considered as poor [33]. About 6% of the consumers that participated in the study chose not to buy food sold at taxi ranks due to food safety issues and hygiene [33].

Food safety risk communication

Qekwana et.al. [25] study in South Africa, observed that communication technology such as cell phones with e-mails, web pages, and Facebook, as well as posters and electronic media (radio and television) can be employed to communicate risk associated with hygienic precautions and food safety at taxi ranks [25].

Occupational health and food safety risk

In South Africa, Qekwana et.al. [20] evaluated the occupational health and food safety risks associated with the traditional slaughter of goats, and the consumption of such meat [20]. Approximately 63% of the practitioners were not wearing protective clothing during slaughter, and about 78% of practitioners did not know their own health status [20]. Almost 83% of the practitioners hung up their carcass to facilitate bleeding, flaying, and evisceration [20]. The study further observed that none of the practitioners practiced meat inspection [20]. In Nigeria, Aluko et al. [31] study revealed that approximately 62% of the vendors had no formal training, and their medical status was also unknown [31].

Discussion

This scoping review mapped evidence on food safety at transport stations in Africa, and revealed a very low number of papers that are published in this area, given many African employees in both formal and informal sectors commute through these transport hubs [12, 13]. An average of one paper per year relating to food safety at transport hubs in Africa as revealed

For peer review only - http://bmjopen.bmj.com/site/about/guidelines.xhtml

by this review is simply not enough. Nonetheless, the few papers depict an imbalance of research, with most focused on microbial safety [4, 19, 22, 23, 28-30, 34], and few on socio-economic aspects such as hygiene practices [6, 21, 31, 32], food safety risk communication [25], and occupational health and food safety risk [20]. Even more worrying is that no study looked at the nutritious aspects of meals sold, despite an established prevalence of poor nutrition and ill-health [37-39]. Moreover, this review revealed no study evaluated the storage of food or how the food is transported to the vending site.

As evidence by this review, most of the food sold at transport hubs do not meet the minimum standards, and is not safe for consumption due to the presence of several microbes [4, 23, 28, 30, 34-36, 40]. There are several reasons for this such as poor practices relating to hygiene, storage, preparation, cooking, cleaning, and serving [4, 19, 22, 23, 28-30, 34]. However, these findings are similar to previous review findings involving markets [41], homes and restaurants [42]. A recent publication by Gizaw [41] indicated that several studies reported microbial contamination of foods sold in the market, with bacteria and fungi similar to those identified in our review [41]. Also, a review by the WHO reported that the main factors contributing to foodborne disease outbreaks in homes or restaurants were poor temperature control in preparing, cooking, and storing food [42]. Although very few papers were found by this review, the evidence is compelling that there should be policy interventions to address issues relating poor hygiene practices, including food storage, preparation, cooking, cleaning, and serving by food handlers at transport hubs, not only in South Africa, but across Africa.

Similar to a previous scoping review [43], most of the included papers were published within the last six years but, no published study was found in 2015 and 2020. Whilst the reason for the lack of published papers in 2015 might be difficult to determine, the COVID-19 pandemic which resulted in "covidisation" of research might be the reason for the lack of publication in this field of research in 2020. Although we cannot conclude that no primary research has been conducted in these countries focusing on the safety of food sold at transport stations, it suggests a research/publication gap. Food safety research is, perhaps, more relevant now than ever in Africa, since the burden of foodborne diseases is rising annually, resulting in the declaration of food safety as a public health concern by the WHO [7, 8]. Aside from this, most commuters tend to buy ready-to-eat (RTE) food from street food vendors, including those at transport hubs [44, 45]; hence, the sale of food at transport stations is rising [45, 46], particularly in Africa [6] partly due to an increase in demand for RTE, and the employment opportunities it offers to many individuals who otherwise would not have had any source of income [43, 47]. Even more

For peer review only - http://bmjopen.bmj.com/site/about/guidelines.xhtml

worrying is the fact that most of the articles included that focused on microbial safety, reported high levels of food contamination with several microorganisms, especially *Salmonella* spp and *E. coli* [4, 23, 28, 30, 34-36, 40]. Therefore, more research is needed across African countries to prevent potential negative consequences.

Our study findings have implications for practice and research. For instance, the likelihood of food poisoning with microbes such as *Salmonella* spp, *E. coli., Shigella* spp*, Bacillus* spp*, S. aureu,* and several others, revealed by most of the included studies that focused on microbial contamination of food, is alarming. This, if not checked, could further worsen the already high burden of foodborne diseases in a continent that has several of its countries already experiencing many health systems and economic challenges. Aside from this, the majority individuals who commute through transport hubs, possible will purchase a meal from a transport hub/exchanges sites, which may be the only meal of the day [12, 13], and yet the food safety standards are poor [4, 19, 22, 23, 28-30, 34]. Thus, if not checked, the excess cases of foodborne diseases from any outbreak will further impact negatively on the already challenged public health systems in Africa. Also, poor people who are exposed to these unsafe food get disease, may have to pay more for healthcare, which can further exacerbate their poverty situation. Moreover, people who are already living in extremely poverty who get exposed to foodborne disease may not even make it to the hospital for care, and can end up dying at home [48].

Good hygiene and sanitation practices, such as adequate hand washing, adequate washing and storage of pots and dishes, good waste management, observation of food preparation standards and serving etiquette, among others, have the potential to reduce the risk of food contamination from both biological and non-biological hazards, yet this study reveal fewer studies that focused on hygienic practices. We, therefore, recommend more research to further inform contextualised policy decisions aimed at improving hygiene and sanitation practices by food vendors at transport stations. Also, very relevant to ensuring food safety is the occupational health practices of the vendors. Regular food handling tests and food inspections, conducted by the appropriate local authorities, should be mandatory in all African countries. Food handler tests should seek to ensure that food vendors are fit healthwise to prepare and serve food meant for public consumption. However, our review found limited studies that evaluated occupational health and food safety. Considering that two studies conducted in South Africa and Nigeria found that 78% and 62% of food vendors at transport stations did not know their health status [20, 31], and the increasing number of informal food sellers at various transport exchange sites,

For peer review only

future studies are recommended to focus on occupational health and food safety in Africa. The means and manner of storing food, especially leftover RTE food, can either increase or reduce the risk of food contamination, but, again, this scoping review found no study that focused on food storage practices of the vendors at transport stations. Also essential, and yet we did not find any study focusing on it, is the quality of food (nutritious aspects) of the meals sold at transport stations. Eating a well nourishing diet or balanced meals is critical to ensure good health [49-51], hence, we encourage future primary studies to include the nutritious aspects. Such studies may help streamline guidelines or inform policies to improve the quality of the food sold at transport exchange sites or taxi ranks. Moreover, this review found that the majority (17 out of 18) of the respondents in the included studies were the vendors (mostly females) or food samples taken from the vendors. The perspectives of consumers (buyers) or commuters regarding food safety at transport stations are also very relevant, and we recommend future research to involve them. A comparative study to investigate food safety practices among males and females food vendors at transport stations might be relevant, since many males are now getting involved in the business [6, 52, 53].

To the best of our knowledge, this study is the first scoping review that systematically mapped literature relating to food safety at transport stations in Africa. A major strength of our study method is that it permits the inclusion of multiple study designs. Also, the choice of this study method permitted us to highlight literature gaps, and made recommendations for future research. Aside from this, we conducted a thorough search in six databases using a comprehensive search strategy which enabled us to capture the most relevant articles to answer the review question. Moreover, two independent reviewers were used to select the studies and perform data extraction processes which helped to prevent selection bias and ensured the reliability and trustworthiness of this study results. Despite this, our scoping review has many limitations. This study included only original study peer reviewed papers, which resulted in the exclusion of one review paper [43], and one Masters' dissertation [54]. We did not also consult the websites of WHO and the Food and Agriculture Organisation websites for possible relevant studies. Furthermore, this study cannot be generalised since the search was limited to African countries only. Although date limitation was removed, we limited the publication language to English only, which perhaps eliminated relevant articles published in other languages. Despite these limitations, this study has provided essential evidence relating to food safety at transport stations and has shown literature gaps to guide future research.

For peer review only - http://bmjopen.bmj.com/site/about/guidelines.xhtml

Conclusion

Based on this scoping review's eligibility criteria, our study results suggest there is limited research focusing on food safety at transport stations in Africa. Most of the existing published studies are focused on microbial safety of food, and very few/none on other aspects such as hygiene practices, food storage, occupational health and food safety, and nutrition. Hence, we recommend more primary research involving community members and policy makers in these areas going forward alongside improving access to clean water/hand washing facilities, and undertaking of structural changes to facilitate behaviours and monitoring for unintended consequences such as livelihoods of vulnerable populations.

Abbreviations

PRISMA- Preferred Reporting Items for Systematic and Meta-analysis

RTE- Ready-to-eat

WHO- World Health Organization

Ethics and dissemination

Not required. All sources of data have been adequately referenced.

Patient and Public Involvement

No patient involved

Statements

Acknowledgments

Support for this work was provided by the Sustainable and Healthy Food Systems (SHEFS) Programme, supported through the Wellcome Trust's Our Planet, Our Health Programme

Authors' contributions

BPN, DK, SED, SM, and RS conceptualized and designed the study. DK developed and designed the database search strategy and conducted the search. PG contributed to the screening of the studies and data extraction. DK wrote the draft manuscript and BPN, SED, GM, and RS critically review it and made revisions. All the authors approved the final version of the manuscript.

Funding

For peer review only - http://bmjopen.bmj.com/site/about/guidelines.xhtml

Funding for this work was provided by the Sustainable and Healthy Food Systems (SHEFS) Programme, supported through the Wellcome Trust's Our Planet, Our Health Programme [grant number: 205200/Z/16/Z]. The funder played no role in the literature search and writing of the manuscript.

Competing interests

None declared

References

1. The Lancet Gastroenterology H. Food safety really is everyone's business. The lancet Gastroenterology & hepatology. 2019;4(8):571.

2. World Health Organization. Estimating the burden of foodborne diseases Geneva: World Health Organization; 2015 [cited 2019 25/10/2019]. Available from: https://www.who.int/activities/estimating-the-burden-of-foodborne-diseases.

3. World Health Organization. Food Safety Geneva: World Health Organization; 2016 [cited 2020 17/11/2020]. Available from: https://www.afro.who.int/health-topics/food-safety.

4. Feglo P, Sakyi K. Bacterial contamination of street vending food in Kumasi, Ghana. Journal of Medical Biomedical Sciences. 2012;1(1):1-8.

5. Organization WH. Food Safety: What you should know Geneva: World Health Organization; 2015 [cited 2019 25/10/2019]. Available from: http://www.searo.who.int/entity/world_health_day/2015/whd-what-you-should-know/en/.

6. Hill J, Mchiza Z, Puoane T, Steyn NP. The development of an evidence-based street food vending model within a socioecological framework: a guide for African countries. PloS one. 2019;14(10):e0223535.

7. World Health Organization. Five keys to safer food manual Geneva: World Health Organization; 2006 [cited 2019 25/10/2019]. Available from: https://www.who.int/foodsafety/publications/consumer/manual_keys.pdf.

8. Lamin-Boima P. Knowledge, Attitude And Practice Of Street Food Vendors In Selected Schools Within Bo City Southern Sierra Leone.

For peer review only - http://bmjopen.bmj.com/site/about/guidelines.xhtml

9. Lues JFR, Rasephei MR, Venter P, Theron MM. Assessing food safety and associated food handling practices in street food vending. International Journal of Environmental Health Research. 2006;16(5):319-28.

10. Abrahale K, Sousa S, Albuquerque G, Padrao P, Lunet N. Street food research worldwide: a scoping review. Journal of human nutrition and dietetics : the official journal of the British Dietetic Association. 2019;32(2):152-74.

11. Liu Z, Zhang G, Zhang X. Urban street foods in Shijiazhuang city, China: Current status, safety practices and risk mitigating strategies. Food Control. 2014;41:212-8.

12. Food and Agriculture Organization. Selling street and snack foods Rome: Food and Agriculture Organization; 2011 [cited 2019 25/10/2019]. Available from: http://www.fao.org/3/a-i2474e.pdf.

13. Gelormini M, Damasceno A, Lopes SA, Malo S, Chongole C, Muholove P, et al. Street Food Environment in Maputo (STOOD Map): a Cross-Sectional Study in Mozambique. JMIR research protocols. 2015;4(3):e98.

14. Arksey H, O'Malley L. Scoping studies: towards a methodological framework. International Journal of Social Research Methodology. 2005;8(1):19-32.

15. Levac D, Colquhoun H, O'Brien KK. Scoping studies: advancing the methodology. Implementation Science. 2010;5(1):69.

16. Ncama BP, Kuupiel D, Duma SE, McHunu G, Guga P, Slotow R. Mapping evidence of food safety at transport stations in Africa: a scoping review protocol. BMJ Open. 2020;10(8):e035879-e.

17. Tricco AC, Lillie E, Zarin W, O'Brien KK, Colquhoun H, Levac D, et al. PRISMA extension for scoping reviews (PRISMA-ScR): checklist and explanation. Annals of internal medicine. 2018;169(7):467-73.

18. Moher D, Liberati A, Tetzlaff J, Altman DG, The PRISMA Group. PRISMA 2009 Flow Diagram. 2009;6(2009):1000097.

19. Oguttu JW, McCrindle CM, Makita K, Grace D. Investigation of the food value chain of ready-to-eat chicken and the associated risk for staphylococcal food poisoning in Tshwane Metropole, South Africa. Food Control. 2014;45:87-94.

For peer review only

For peer review only - http://bmjopen.bmj.com/site/about/guidelines.xhtml

20. Qekwana DN, McCrindle CM, Oguttu JW, Grace D. Assessment of the occupational health and food safety risks associated with the traditional slaughter and consumption of goats in Gauteng, South Africa. International Journal of Environmental Research. 2017;14(4):420.

21. Kok R, Balkaran R. Street food vending and hygiene practices and implications for consumers. Journal of Economics Behavioral Studies. 2014;6(3):188-93.

22. Mafune TS, Takalani TK, Anyasi TA, Ramashia SE. Microbial Safety of Street Vended Foods Sold in Thohoyandou, South Africa. Journal of Human Ecology. 2016;53(3):205-12.

23. Mazizi B, Muchenje V, Makepe M, Mutero G. Assessment of Aerobic Plate Counts, Staphylococcus aureus, Escherichia coli and Salmonella in Meat Sold by Street Vendors in the Eastern Cape Province, South Africa. Journal of Food Nutrition Research. 2017;5(6):436-42.

24. Hill J, McHiza Z, Puoane T, Steyn NP. Food sold by street-food vendors in Cape Town and surrounding areas: a focus on food and nutrition knowledge as well as practices related to food preparation of street-food vendors. Journal of Hunger & Environmental Nutrition. 2019;14(3):401-15.

25. Qekwana DN, McCrindle CME, Oguttu JW. Designing a risk communication strategy for health hazards posed by traditional slaughter of goats in Tshwane, South Africa. Journal of the South African Veterinary Association. 2014;85(1).

26. Qekwana ND, Oguttu JW. Assessment of Food Safety Risks Associated with Preslaughter Activities during the Traditional Slaughter of Goats in Gauteng, South Africa. Journal of Food Protection. 2014;77(6):1031-7.

27. McArthur-Floyd M, Commey V, Boakye NAB. Evaluation of Food Safety among Fast Food Operators in Madina, Accra. Evaluation. 2016;54.

28. Abakari G, Cobbina SJ, Yeleliere E. Microbial quality of ready-to-eat vegetable salads vended in the central business district of tamale, Ghana. International Journal of Food Contamination. 2018;5(1).

29. Kibret M, Tadesse M. The bacteriological safety and antimicrobial susceptibility of bacteria isolated from street-vended white lupin (Lupinus albus) in Bahir Dar, Ethiopia. Ethiopian Journal of Health Sciences. 2013;23(1):19-26.

For peer review only - http://bmjopen.bmj.com/site/about/guidelines.xhtml

30. Eromo T, Tassew H, Daka D, Kibru G. Bacteriological Quality of Street Foods and Antimicrobial Resistance of Isolates in Hawassa, Ethiopia. Ethiopian Journal of Health Sciences. 2016;26(6):533-42.

31. Aluko OO, Ojeremi TT, Olaleke DA, Ajidagba EB. Evaluation of food safety and sanitary practices among food vendors at car parks in Ile Ife, southwestern Nigeria. Food Control. 2014;40:165-71.

32. Odundo A, Okemo P, Chege P. An Assessment of Food Safety Practices among Street Vendors in Mombasa, Kenya. International Journal of Health Sciences Research. 2018;8(5):235-43.

33. Letuka P, Nkhebenyane J, Thekisoe O. Assessment of Food Safety Knowledge, Attitudes and Practices among Street Food Vendors and Consumers' Perceptions of Street Food Vending in Maseru Lesotho. 2019.

34. Jermini M, Bryan FL, Schmitt R, Mwandwe C, Mwenya J, Zyuulu MH, et al. Hazards and Critical Control Points of Food Vending Operations in a City in Zambia. J Food Prot. 1997;60(3):288-99.

35. Tshipamba M, Lubanza N, Adetunji M, Mwanza MJJFMSH. Evaluation of the Effect of Hygiene Practices and Attitudes on the Microbial Quality of Street Vended Meats Sold in Johannesburg, South-Africa. 2018;3(137):2476-059.1000137.

36. Oguttu JW, McCrindle CME, Makita K, Grace D. Investigation of the food value chain of ready-to-eat chicken and the associated risk for staphylococcal food poisoning in Tshwane Metropole, South Africa. Food Control. 2014;45:87-94.

37. Caristia S, Filigheddu N, Barone-Adesi F, Sarro A, Testa T, Magnani C, et al. Vitamin D as a Biomarker of Ill Health among the Over-50s: A Systematic Review of Cohort Studies. Nutrients. 2019;11(10).

38. Huynh G, Huynh QHN, Nguyen NHT, Do QT, Khanh Tran V. Malnutrition among 6-59-Month-Old Children at District 2 Hospital, Ho Chi Minh City, Vietnam: Prevalence and Associated Factors. Biomed Res Int. 2019;2019:6921312.

39. Oftedal S, Glozier N, Holliday EG, Duncan MJ. Diet quality and depressive symptoms. Assessing the direction of the association in a population-based cohort study. Journal of affective disorders. 2020;274:347-53.

For peer review only - http://bmjopen.bmj.com/site/about/guidelines.xhtml

40. Kibret M, Tadesse MJEjohs. The bacteriological safety and antimicrobial susceptibility of bacteria isolated from street-vended white lupin (Lupinus albus) in Bahir Dar, Ethiopia. 2013;23(1):19-26.

41. Gizaw Z. Public health risks related to food safety issues in the food market: a systematic literature review. Environmental Health and Preventive Medicine. 2019;24(1):68.

42. Todd EC. Epidemiology of foodborne diseases: a worldwide review. World health statistics quarterly Rapport trimestriel de statistiques sanitaires mondiales. 1997;50(1-2):30-50.

43. Abrahale K, Sousa S, Albuquerque G, Padrao P, Lunet N. Street food research worldwide: a scoping review. Journal of Human Nutrition and Dietetics. 2019;32(2):152-74.

44. Bellia C, Pilato M, Seraphin H. Street food and food safety: a driver for tourism? Calitatea. 2016;17(S1):20.

45. Mosupye FM, Von Holy A. Microbiological hazard identification and exposure assessment of street food vending in Johannesburg, South Africa. International Journal of Food Microbiology

2000;61(2-3):137-45.

46. Mosupye FM, von Holy A. Microbiological quality and safety of ready-to-eat street-vended foods in Johannesburg, South Africa. International Journal of Food Microbiology. 1999;62(11):1278-84.

47. Muzaffar AT, Huq I, Mallik BA. Entrepreneurs of the streets: An analytical work on the street food vendors of Dhaka City. International journal of Business

Management. 2009;4(2):80-8.

48. World Health Organisation. Universal Health Coverage Geneva2020 [cited 2021 09/04/2021]. Available from: https://www.who.int/health-topics/universal-health-coverage#tab=tab_1.

49. Melanson K. Nutrition review: lifestyle approaches to promoting healthy eating for children. American journal of lifestyle medicine. 2008;2(1):26-9.

For peer review only

For peer review only - http://bmjopen.bmj.com/site/about/guidelines.xhtml

50. Carrier N, Villalon L, Lengyel C, Slaughter SE, Duizer L, Morrison-Koechl J, et al. Diet quality is associated with malnutrition and low calf circumference in Canadian long-term care residents. BMC nutrition. 2019;5:57.

51. Freeland-Graves JH, Nitzke S. Position of the academy of nutrition and dietetics: total diet approach to healthy eating. Journal of the Academy of Nutrition

Dietetics. 2013;113(2):307-17.

52. Okojie P, Isah E. Sanitary conditions of food vending sites and food handling practices of street food vendors in Benin City, Nigeria: implication for food hygiene and safety. Journal of environmental public Health Reports. 2014;2014.

53. da Silva SA, Cardoso RdCV, Góes JÂW, Santos JN, Ramos FP, de Jesus RB, et al. Street food on the coast of Salvador, Bahia, Brazil: A study from the socioeconomic and food safety perspectives. Food control. 2014;40:78-84.

54. Acheampong BE. Assessment of food hygiene practices by street food vendors and microbial quality of selected foods sold. A Study at Dunkwa-On-Offin, Upper Denkyira East Municipality of the Central Region 2015.

For peer review only - http://bmjopen.bmj.com/site/about/guidelines.xhtml

542 **Table 1:** Characteristic of the included sources of evidence

**Author, year**	**Country**	**City/Town**	**Study design**	**Study setting**	**Study population**	**Sample size**	**Sex of vendors**	**Outcome reported**
Oguttu et. al., 2014 [19]	South Africa	Tshwane Metropole, Gauteng Province	Mixed-methods study	Taxi rank	Vendors selling Ready–to-eat chicken	100 samples of Ready–to-eat chicken	Females	Microbial safety of food
Mafune et. al., 2016 [22]	South Africa	Thohoyandou, Limpopo Province	Cross-sectional study	Taxi rank, bus station, shopping mall, and street stalls	Food samples from street vendors	28 samples	Not specified	Microbial safety of food
Kibret et. al., 2013 [29]	Ethiopia	Bahir Dar Town	Cross-sectional study	Main roads sites, bus station, groceries, taxi ranks	Ready–to-eat white lupin sample from vendors	40 samples (200 grams of white lupin)	Not specified	Microbial safety of food
Abakari et. al., 2018 [28]	Ghana	Tamale, Northern Region	Cross-sectional study	Taxi rank, bus stops, transport yard, and timber market	Ready-to-eat salad samples from food vendors	30 salad samples	Not specified	Microbial safety of food
Aluko et.al., 2014 [31]	Nigeria	Ile Ife, southwestern Nigeria	Cross-sectional study	Car parks	Food vendors	160 (117 stationery and 43 mobile vendors)	Males and females	Hygiene practices of food handlers/vendors
Odundo et. al., 2018 [32]	Kenya		Cross-sectional study	Major bus stops, markets, shopping areas, construction	Food vendors	130	Males and females	Hygiene practices of food handlers/vendors

For peer review only - http://bmjopen.bmj.com/site/about/guidelines.xhtml

				sites, and commercial areas				
Kok et. al., 2014 [21]	South Africa	Durban, KwaZulu-Natal Province	Cross-sectional study	Transport exchange site	Food vendors	29	Not specified	Hygiene practices of food handlers/vendors
Letuka et. al., 2019 [33]	Lesotho	Maseru	Cross-sectional study	Taxi ranks	Food vendors	141 (48 food handlers and 93 consumers)	Male and female	Knowledge of food safety measures and hygiene practice by food handlers/vendors
Eromo et. al., 2016 [30]	Ethiopia	Hawassa City	Cross-sectional study	Bus station	Food samples from street food vendors	72 samples from six food items	Not specified	Microbial safety of food
McArthur-Floyd et. al., 2016 [27]	Ghana	Madina (Accra), Greater Accra Region	Cross-sectional study	Taxi rank, and transport exchange sites	Food vendors	200	Males and females	Knowledge of hygiene practice
Hill et. al., 2019 [6]	South Africa	Cape Town	Cross-sectional study	Train, bus stations, and taxi ranks, community centers, market	Food vendors	831	Males and females	Hygiene practices of food handlers/vendors
Mazizi et. al., 2017 [23]	South Africa	Alice (Nkonkobe) and King William's Town (Buffalo City), Eastern Cape province	Cross-sectional study	Taxi rank and bus stations	Street food vendors	136 food samples-cooked and raw.	Not specified	Microbial safety of food

For peer review only - http://bmjopen.bmj.com/site/about/guidelines.xhtml

Qekwana et.al, 2014 [26]	South Africa	Tshwane, Gauteng Province	Cross-sectional study	Taxi ranks and informal markets	Traditional goat slaughters	105 people	Males and females	Knowledge of food safety precautions
Qekwana et.al, 2014 [25]	South Africa	Tshwane, Gauteng Province	Cross-sectional study	Taxi ranks	Street food vendors	105 people	Males and females	Hygiene precautions communication channel for food safety
Qekwana et.al, 2017 [20]	South Africa	Tshwane Metropolitan Municipality, Gauteng Province	Cross-sectional study	Taxi ranks and Informal markets	Traditional goat slaughter	105 people	Males and females	Occupational health and food safety risk
Flego et. al., 2012 [4]	Ghana	Kumasi, Ashanti Region	Cross-sectional study	Bus terminals	Food samples from vendors	60 food samples	Not specified	Microbial safety of food
Tshipamba et. al., 2018 [35]	South Africa	Johannesburg	Cross-sectional study	Taxi ranks and streets	Meat samples from vendors	115 meat samples	Not specified	Hygiene practices of food handlers/vendors, and microbial safety of food
Jermini et. al., 1997 [34]	Zambia	Not specified	Cross-sectional study	bus park/station and large market	Samples of raw, processed, and cooked Foods from street food vendors	Not specified	Not specified	Microbial safety of food

543

For peer review only - http://bmjopen.bmj.com/site/about/guidelines.xhtml

Table 2: Microbial safety of food at transport stations

Study	Type of sample	Analytical approach	Microbes reported	Key results	Key conclusion
Oguttu et. al. [19]	Ready-to-eat (RTE) chicken	3M™ Petrifilm™ plates	*S. aureus*	• High prevalence of *S. aureus* in the sample was (44%; 90% CI: 36.1%-52.2%), with mean *S. aureus* counts of $10^{3.6}$ (90%CI: $10^{3.3}$-$10^{3.9}$).<br>• The likelihood of food poisoning with *S. aureus* from RTE chicken was estimated to be 1.3% (90% CI: 0% -2.7%)	To reduce the levels of concentration of *S. aureus* on the RTE chicken and promote the sale of safer and affordable RTE chicken for the large urban poor population in South Africa, training of RTE chicken vendors on hygiene is still needed.
Mafune et. al. [22]	Unfermented porridge, boiled cabbage and carrots, boiled peanuts, salad, potato chips, traditional mageu, and stewed beef and grilled chicken	Standard microbiological method	*S. aureus*	• *S. aureus* was <2.4771 log10 cfu/g in all samples and places.<br>• Except for fried potato chips, microbial contamination was observed in the remaining food samples using the total plate count method.	Most of the vended foods investigated met the microbiological standard of RTE foods
Mazizi et. al. [23]	Cooked and raw beef, pork, and mutton samples, surface contact plates, and water samples	Biochemical tests according to international standards methods	*S. aureus, E. coli*, and *Salmonella spp.*	• Mean score of raw beef, mutton, and pork were aerobic plate counts (4.8, 3.7 and 2.8 Log (cfu/g)), *S. aureus* (3.3, 3.7 and 2.8 Log cfu/g), and *E. coli* (1.0, 0.6 and 0.3 Log cfu/g) respectively.	The levels of contamination in cooked meat were lower when compared to the standards set by Commission Regulation for determining the microbiological quality of RTE foods.

For peer review only

For peer review only - http://bmjopen.bmj.com/site/about/guidelines.xhtml

Tshipamba et. al. [35]	RTE meat	Standard biochemical and Molecular methods	*Bacillus thuringienis, Bacillus spp., Bacillus subtilis, Bacillus cereus, Citrobacter spp., Enterococcus faecium, Enterococcus faecalis, Kurthia spp., Lysinibacillus spp. Macrococcus caseolyticus, Planomicrobium glaciei, Planococcus antarcticus, S. aureus, S. equorum*, and *S. vitulinus*	• Overall mean total bacteria in the samples ranged from 4.3-6.03 cfu/ml × 102 and coliform counts ranged from 1.60-1.95 × 102 cfu/ml<br>• Of the 15 microbes identified, S. aureus occurred in all the meat types and the percentage of occurrence was chicken meat (14%), beef head (43%), beef intestine (50%), and wors (sausage) (20%)	Consumers RTE meat are at risk of food borne diseases due to poor hygiene practices of the vendors.
Kibret et. al. [29]	White lupin	Standard bacteriological techniques, and Kirby-Bauer disk	*E. coli, Salmonella spp, and Shigella spp.*	• Prevalence of bacteria total coliform counts were 954.2±385 at the surface and 756.2±447.3 at the core of white lupin.	Contamination of white lupin and a potential health risk to consumers revealed, and the bacteria isolated showed high rates of multiple drug resistance.

		diffusion method for antimicrobial susceptibility test		• Pathogens isolated were as follows *E. coli* 29 (72.5%), *Salmonella* spp. 23 (57.5%) and *Shigella* spp. 8 (20%).<br>• Overall multiple antimicrobial resistances rate was 75%	
Eromo et. al. [30]	Local bread ('ambasha' and'kita'), raw fish, chilli ('awaze'), avocado, and cooked potato	Standard microbiological techniques	*E. scoli, Salmonella spp.*, and *S. aureus*	• The microbiological quality in nearly 31% of RTE food samples was beyond the acceptable limits.<br>• Total colony counts detected ranged from 1.7x105 to 6.7x106 cfu/g.<br>• *E.coli* (29.6%), *Salmonella* spp. (12.7%, and *S. aureus* (9.9%) were the most frequent isolates.<br>• All isolates were 100% sensitive to ciprofloxacin, but 89% of *Salmonella* spp. was resistant to chloramphenicol, 14.3% of *S.aureus* was resistant to vancomycin	Considerable rate of contamination in the foods confirmed. The identified foodborne bacteria and antibiotic resistance isolates could pose a public health problem in the study location.
Abakari et. al. [28]	Pre-cut vegetable salads	Standard microbiological methods	*E. coli, Bacillus cereus, Salmonella spp, and Shigella spp.*	• *E. coli* levels ranged from 0 to 7.56 log10 cfu/g; *Bacillus cereus* levels ranged from 0 to 7.44 log10 cfu/g; *Salmonella* spp. ranged from 0 to 4.54 log10 cfu/g, and *Shigella* spp. ranged from 5.54 log10 cfu/g were detected in 96.7%, 93.3%, 73.3%, and 76.7% of the salads samples, respectively.	Salads were revealed to be unwholesome for human consumption and could be deleterious to the health of consumers.

For peer review only - http://bmjopen.bmj.com/site/about/guidelines.xhtml

Flego et. al. [4]	RTE foods (ice-kenkey (15), cocoa drink (15), fufu (5), ready-to-eat red pepper for kenkey) (5), salad (10), and macaroni (10))	Standard microbiological methods	*Staphylococci*, *Bacillus* spp., *Klebsiella pneumoniae*, *Aeromonas pneumophila*, *E. cloacae*, *S. aureus*, *E. coli*, and *P. aeruginosa*	• RTE foods were found to be contaminated with enteric bacteria and other potential food poisoning organisms with bacterial counts higher than the acceptable levels (<5.0 log10 CFU/ml).<br>• Coagulate negative staphylococci (23.7%), Bacillus species (21.5%), *K. pneumoniae* (18.0%), *Aeromonas pneumophila* (17.7%), E. cloacae (6.7%), *S. aureus* (3.7%), E. coli (2.2%) and *P. aeruginosa* (2.2%) were the main isolates detected.	Most RTE foods were contaminated with enteric bacteria and other potential food poisoning organisms with bacterial counts higher than the acceptable levels.
Jermini et. al. [34]	Raw foods (ground meat, chicken, and chicken intestine); and processed foods (dried "minnows" and "kapenta")		*Salmonellae Spp.*, *S. aureus*, *Clostridium peifringens*	• Raw foods such as ground meat, chicken, chicken intestine; and processed foods such as dried "minnows" and "kapenta" were contaminated by salmonellae or contained high populations of S. aureus in pasteurized milk.<br>• High populations (> 105) of S. aureus were detected from a sample of leftover chicken, more than 107 were detected in leftover rice, and 10 million *C. peifringens* per gram were detected in leftover beef stew sample	Time-temperature exposures during reheating had variable effects in terms of killing the microorganisms that germinated from surviving spores or that reached the foods after cooking.

For peer review only - http://bmjopen.bmj.com/site/about/guidelines.xhtml

Table 3: Key reported findings on food preparation

Study	Key findings reported
Kok et. al. [21]	• Water being used for washing utensils was left unchanged,<br>• Piles of dirty pots and dishes was left near the serving areas and RTE foods, and garbage left uncovered with many flies at the site,<br>• RTE food was left uncovered,<br>• Most of the food handlers were not wearing gloves, hairnets, or aprons
Hill et. al. [6]	• 85.5% of the vending stalls lacked soap or surface sanitizer,<br>• 71% lacked basin for washing,<br>• 75% did not have drying cloth,<br>• 76.6% of vendors handled food and money concurrently,<br>• About 57% left the food uncovered.<br>• 39% of the vendors were using their hands to pick up food items, with only 6% wearing gloves, and<br>• 29% of vendors had a wet clean sponge/cloth obtainable at the site
Mazizi et. al. [23]	• Major sources of food contamination identified were poor hygiene practices of the food vendor, holding area, and the utensils
Tshipamba et. al. [35]	• Approximately 90% of RTE meat vendors at the taxi rank exposed their meats to dust and flies,<br>• 94% of them handled money whilst serving food, and<br>• Stagnant water found in about 22% of the vending locations at the taxi-rank
McArthur-Floyd et. al. [27]	• 64% of food vendors washed their hands from elbow to finger and the remainder (36%) washed from their wrist to finger (the WHO recommends handwashing from elbow to fingers), and<br>• 62% of the vendors test their meal in the palm whilst 38% of them test it with a spoon (the best way to test a meal)
Aluko et. al. [31]	• Approximately 17% of food vendors washed their hands always after using the toilet,<br>• 63% of them rarely kept their fingernails short, and<br>• Nearly 4% of them always kept their leftover cooked food in a refrigerator, despites having unstable power supply
Odundo et. al. [32]	• Food vendors had poor hygiene practices however, men were observed to have better hygienic practices than women ($P<0.05$),<br>• Hygiene practice of the vendors was found to be significantly associated with training (those trained observe hygiene), and<br>• Wearing of jewellery, long and unclean nails, and lack of protective clothing were observed.
Letuka et. al. [33]	• Observed that the food handlers operated under unhygienic environment

Figure

Figure 1: PRISMA 2009 Flow Diagram

Figure 2: Trend of published studies relating to food safety at transport station in Africa

Supplementary File

Supplementary file 1: PRISMA-P Checklist

For peer review only - http://bmjopen.bmj.com/site/about/guidelines.xhtml

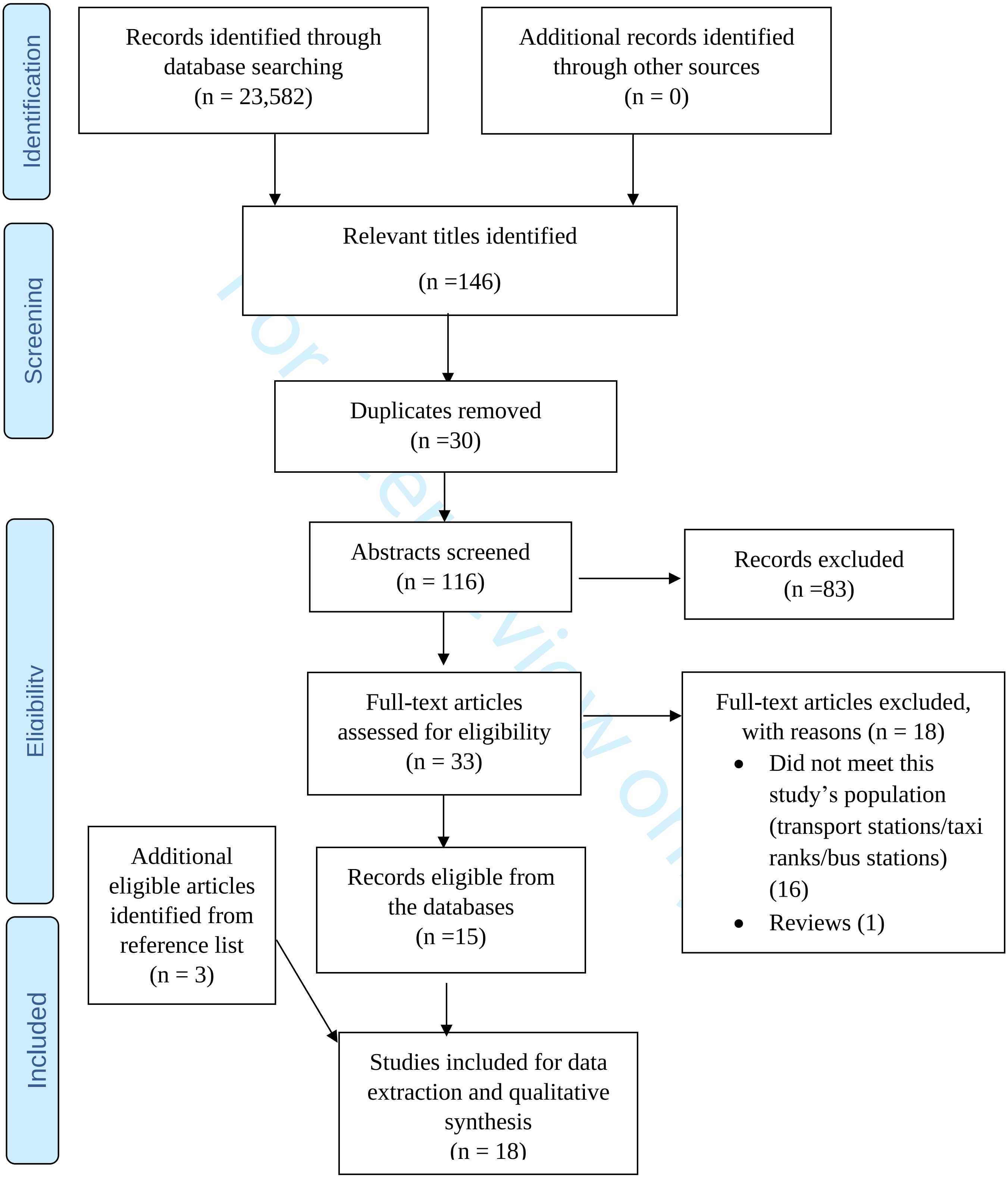

Figure 1: PRISMA 2009 Flow Diagram [18]

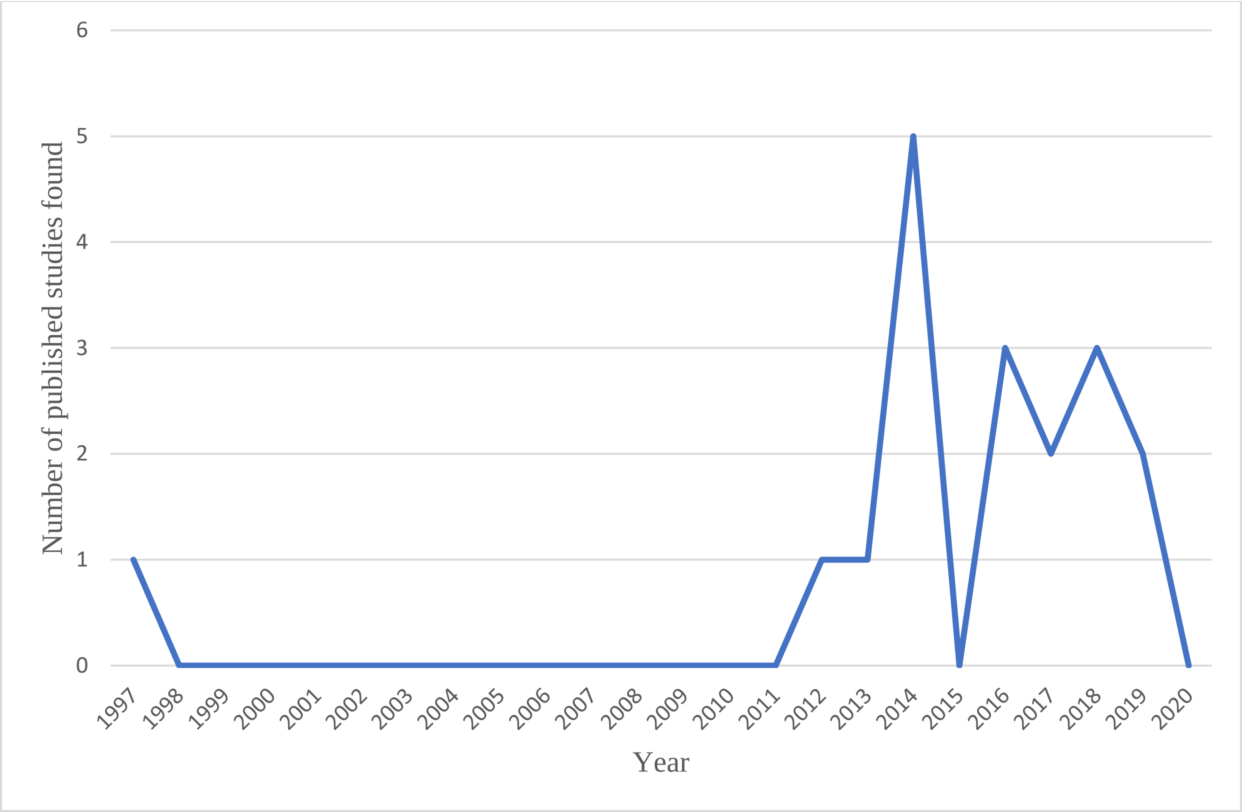

Figure 2: Trend of published studies relating to food safety at transport station in Africa

Supplementary file 1: Electronic databases search

Search date	Database	Keywords	Search results	Number eligible
09/11/2019	EBSCOhost (Academic search complete, CINAHL with Full-text, and Health Source	SU food safety AND SU (food preparation or meal preparation or cooking) OR SU food handling OR SU food storage OR hygiene practices AND (food trading or food selling or food vending or street food) AND (transport station or taxi rank or bus station or transport exchange sites or car park or lorry park) AND africa	2,549	14
10/11/2019	PubMed	"food safety"[MeSH Terms] OR ("food"[All Fields] AND "safety"[All Fields]) OR "food safety"[All Fields]) OR ("food supply"[MeSH Terms] OR ("food"[All Fields] AND "supply"[All Fields]) OR "food supply"[All Fields] OR ("food"[All Fields] AND "security"[All Fields]) OR "food security"[All Fields]) AND (("food"[MeSH Terms] OR "food"[All Fields]) AND vending[All Fields]) OR (("food"[MeSH Terms] OR "food"[All Fields]) AND trading[All Fields]) AND streets[All Fields] OR (("motor vehicles"[MeSH Terms] OR ("motor"[All Fields] AND "vehicles"[All Fields]) OR "motor vehicles"[All Fields] OR "lorry"[All Fields]) AND parks[All Fields]) OR (("motor vehicles"[MeSH Terms] OR ("motor"[All Fields] AND "vehicles"[All Fields]) OR "motor vehicles"[All Fields] OR "lorry"[All Fields]) AND station[All Fields]) OR (taxi[All Fields] AND ranks[All Fields]) AND ("algeria"[MeSH Terms] OR "algeria"[All Fields]) OR ("angola"[MeSH Terms] OR "angola"[All Fields]) OR ("benin"[MeSH Terms] OR "benin"[All Fields]) OR ("botswana"[MeSH Terms] OR "botswana"[All Fields]) OR ("burkina faso"[MeSH Terms] OR ("burkina"[All Fields] AND "faso"[All Fields]) OR "burkina faso"[All Fields]) OR ("burundi"[MeSH Terms] OR "burundi"[All Fields]) OR ("cameroon"[MeSH Terms] OR "cameroon"[All Fields]) OR ("cabo verde"[MeSH Terms] OR ("cabo"[All Fields] AND "verde"[All Fields]) OR "cabo verde"[All Fields] OR ("cape"[All Fields] AND "verde"[All Fields]) OR "cape verde"[All Fields]) OR ("central african republic"[MeSH Terms]	2,834	33

For peer review only

		OR ("central"[All Fields] AND "african"[All Fields] AND "republic"[All Fields]) OR "central african republic"[All Fields]) OR ("chad"[MeSH Terms] OR "chad"[All Fields]) OR ("democratic republic of the congo"[MeSH Terms] OR ("democratic"[All Fields] AND "republic"[All Fields] AND "congo"[All Fields]) OR "democratic republic of the congo"[All Fields]) OR ("congo"[MeSH Terms] OR "congo"[All Fields] OR ("republic"[All Fields] AND "congo"[All Fields]) OR "republic of the congo"[All Fields]) OR ("djibouti"[MeSH Terms] OR "djibouti"[All Fields]) OR ("egypt"[MeSH Terms] OR "egypt"[All Fields]) OR ("equatorial guinea"[MeSH Terms] OR ("equatorial"[All Fields] AND "guinea"[All Fields]) OR "equatorial guinea"[All Fields]) OR ("eritrea"[MeSH Terms] OR "eritrea"[All Fields]) OR ("ethiopia"[MeSH Terms] OR "ethiopia"[All Fields]) OR ("gabon"[MeSH Terms] OR "gabon"[All Fields]) OR ("gambia"[MeSH Terms] OR "gambia"[All Fields]) OR ("ghana"[MeSH Terms] OR "ghana"[All Fields]) OR ("guinea"[MeSH Terms] OR "guinea"[All Fields]) OR ("guinea-bissau"[MeSH Terms] OR "guinea-bissau"[All Fields] OR ("guinea"[All Fields] AND "bissau"[All Fields]) OR "guinea bissau"[All Fields]) OR ("cote d'ivoire"[MeSH Terms] OR ("cote"[All Fields] AND "d'ivoire"[All Fields]) OR "cote d'ivoire"[All Fields] OR ("ivory"[All Fields] AND "coast"[All Fields]) OR "ivory coast"[All Fields]) OR ("kenya"[MeSH Terms] OR "kenya"[All Fields]) OR ("lesotho"[MeSH Terms] OR "lesotho"[All Fields]) OR ("liberia"[MeSH Terms] OR "liberia"[All Fields]) OR ("libya"[MeSH Terms] OR "libya"[All Fields]) OR ("madagascar"[MeSH Terms] OR "madagascar"[All Fields]) OR ("malawi"[MeSH Terms] OR "malawi"[All Fields]) OR ("mali"[MeSH Terms] OR "mali"[All Fields]) OR ("mauritania"[MeSH Terms] OR "mauritania"[All Fields]) OR ("mauritius"[MeSH Terms] OR "mauritius"[All Fields]) OR ("morocco"[MeSH Terms] OR "morocco"[All Fields]) OR ("mozambique"[MeSH Terms] OR "mozambique"[All Fields]) OR ("namibia"[MeSH Terms] OR "namibia"[All Fields]) OR ("niger"[MeSH Terms] OR "niger"[All Fields]) OR ("nigeria"[MeSH Terms] OR "nigeria"[All Fields]) OR ("rwanda"[MeSH Terms] OR		

For peer review only

		"rwanda"[All Fields]) OR ("sao tome and principe"[MeSH Terms] OR ("sao"[All Fields] AND "tome"[All Fields] AND "principe"[All Fields]) OR "sao tome and principe"[All Fields]) OR ("senegal"[MeSH Terms] OR "senegal"[All Fields]) OR ("seychelles"[MeSH Terms] OR "seychelles"[All Fields]) OR ("sierra leone"[MeSH Terms] OR ("sierra"[All Fields] AND "leone"[All Fields]) OR "sierra leone"[All Fields]) OR ("somalia"[MeSH Terms] OR "somalia"[All Fields]) OR (("south africa"[MeSH Terms] OR ("south"[All Fields] AND "africa"[All Fields]) OR "south africa"[All Fields]) AND South[All Fields]) OR ("sudan"[MeSH Terms] OR "sudan"[All Fields]) OR ("sudan"[MeSH Terms] OR "sudan"[All Fields]) OR ("eswatini"[MeSH Terms] OR "eswatini"[All Fields] OR "swaziland"[All Fields]) OR ("tanzania"[MeSH Terms] OR "tanzania"[All Fields]) OR ("togo"[MeSH Terms] OR "togo"[All Fields]) OR ("tunisia"[MeSH Terms] OR "tunisia"[All Fields]) OR ("uganda"[MeSH Terms] OR "uganda"[All Fields]) OR ("zambia"[MeSH Terms] OR "zambia"[All Fields]) OR ("zimbabwe"[MeSH Terms] OR "zimbabwe"[All Fields]) Filters: Free full text, Comparative Study, Observational Study, Randomized Controlled Trial, Humans, English, MEDLINE		
11/11/2019	Web of Science	(food safety AND food preparation OR meal preparation OR cooking OR food handling OR food storage OR hygiene practices AND food trading OR food selling OR food vending or street food AND transport station OR taxi rank OR bus station OR transport exchange sites OR car park or lorry park AND africa)Refined by: Open Access: (OPEN ACCESS) AND DOCUMENT TYPES: (ARTICLE) AND DOCUMENT TYPES: (ARTICLE) AND WEB OF SCIENCE CATEGORIES: (FOOD SCIENCE TECHNOLOGY OR PUBLIC ENVIRONMENTAL OCCUPATIONAL HEALTH) AND WEB OF SCIENCE CATEGORIES: (FOOD SCIENCE TECHNOLOGY OR PUBLIC ENVIRONMENTAL OCCUPATIONAL HEALTH OR NUTRITION DIETETICS)Timespan: All years. Indexes: SCI-EXPANDED, SSCI, A&HCI, CPCI-S, CPCI-SSH, BKCI-S, BKCI-SSH, ESCI.	8,263	36
12/11/2019	SCOPUS	food safety AND food preparation OR meal preparation OR cooking OR food handling OR food storage OR hygiene practices AND food trading OR	116	7

For peer review only - http://bmjopen.bmj.com/site/about/guidelines.xhtml

		food selling OR food vending or street food AND transport station OR taxi rank OR bus station OR transport exchange sites OR car park or lorry park AND africa		
13/11/2019	Google Scholar	food safety AND food preparation OR meal preparation OR cooking OR food handling OR food storage OR hygiene practices AND food trading OR food selling OR food vending or street food AND transport station OR taxi rank OR bus station OR transport exchange sites OR car park or lorry park AND africa	9,820	56
			23,582	146
**Duplicates**				**30**

For peer review only

Supplementary file 1: Preferred Reporting Items for Systematic reviews and Meta-Analyses extension for Scoping Reviews (PRISMA-ScR) Checklist

SECTION	ITEM	PRISMA-ScR CHECKLIST ITEM	REPORTED ON PAGE #
**TITLE**			
Title	1	Identify the report as a scoping review.	
**ABSTRACT**			
Structured summary	2	Provide a structured summary that includes (as applicable): background, objectives, eligibility criteria, sources of evidence, charting methods, results, and conclusions that relate to the review questions and objectives.	
**INTRODUCTION**			
Rationale	3	Describe the rationale for the review in the context of what is already known. Explain why the review questions/objectives lend themselves to a scoping review approach.	
Objectives	4	Provide an explicit statement of the questions and objectives being addressed with reference to their key elements (e.g., population or participants, concepts, and context) or other relevant key elements used to conceptualize the review questions and/or objectives.	
**METHODS**			
Protocol and registration	5	Indicate whether a review protocol exists; state if and where it can be accessed (e.g., a Web address); and if available, provide registration information, including the registration number.	
Eligibility criteria	6	Specify characteristics of the sources of evidence used as eligibility criteria (e.g., years considered, language, and publication status), and provide a rationale.	
Information sources*	7	Describe all information sources in the search (e.g., databases with dates of coverage and contact with authors to identify additional sources), as well as the date the most recent search was executed.	
Search	8	Present the full electronic search strategy for at least 1 database, including any limits used, such that it could be repeated.	
Selection of sources of evidence†	9	State the process for selecting sources of evidence (i.e., screening and eligibility) included in the scoping review.	
Data charting process‡	10	Describe the methods of charting data from the included sources of evidence (e.g., calibrated forms or forms that have been tested by the team before their use, and whether data charting was done independently or in duplicate) and any processes for obtaining and confirming data from investigators.	
Data items	11	List and define all variables for which data were sought and any assumptions and simplifications made.	
Critical appraisal of individual sources of evidence§	12	If done, provide a rationale for conducting a critical appraisal of included sources of evidence; describe the methods used and how this information was used in any data synthesis (if appropriate).	
Synthesis of results	13	Describe the methods of handling and summarizing the data that were charted.	

St. Michael's
Inspired Care.
Inspiring Science.

SECTION	ITEM	PRISMA-ScR CHECKLIST ITEM	REPORTED ON PAGE #
**RESULTS**			
Selection of sources of evidence	14	Give numbers of sources of evidence screened, assessed for eligibility, and included in the review, with reasons for exclusions at each stage, ideally using a flow diagram.	
Characteristics of sources of evidence	15	For each source of evidence, present characteristics for which data were charted and provide the citations.	
Critical appraisal within sources of evidence	16	If done, present data on critical appraisal of included sources of evidence (see item 12).	
Results of individual sources of evidence	17	For each included source of evidence, present the relevant data that were charted that relate to the review questions and objectives.	
Synthesis of results	18	Summarize and/or present the charting results as they relate to the review questions and objectives.	
**DISCUSSION**			
Summary of evidence	19	Summarize the main results (including an overview of concepts, themes, and types of evidence available), link to the review questions and objectives, and consider the relevance to key groups.	
Limitations	20	Discuss the limitations of the scoping review process.	
Conclusions	21	Provide a general interpretation of the results with respect to the review questions and objectives, as well as potential implications and/or next steps.	
**FUNDING**			
Funding	22	Describe sources of funding for the included sources of evidence, as well as sources of funding for the scoping review. Describe the role of the funders of the scoping review.	

JBI = Joanna Briggs Institute; PRISMA-ScR = Preferred Reporting Items for Systematic reviews and Meta-Analyses extension for Scoping Reviews.

* Where *sources of evidence* (see second footnote) are compiled from, such as bibliographic databases, social media platforms, and Web sites.

† A more inclusive/heterogeneous term used to account for the different types of evidence or data sources (e.g., quantitative and/or qualitative research, expert opinion, and policy documents) that may be eligible in a scoping review as opposed to only studies. This is not to be confused with *information sources* (see first footnote).

‡ The frameworks by Arksey and O'Malley (6) and Levac and colleagues (7) and the JBI guidance (4, 5) refer to the process of data extraction in a scoping review as data charting.

§ The process of systematically examining research evidence to assess its validity, results, and relevance before using it to inform a decision. This term is used for items 12 and 19 instead of "risk of bias" (which is more applicable to systematic reviews of interventions) to include and acknowledge the various sources of evidence that may be used in a scoping review (e.g., quantitative and/or qualitative research, expert opinion, and policy document).

From: Tricco AC, Lillie E, Zarin W, O'Brien KK, Colquhoun H, Levac D, et al. PRISMA Extension for Scoping Reviews (PRISMA-ScR): Checklist and Explanation. Ann Intern Med. ;169:467–473. doi: 10.7326/M18-0850

For peer review only - http://bmjopen.bmj.com/site/about/guidelines.xhtml

BMJ Open

A Scoping Review of Food Safety at Transport stations in Africa

Journal:	*BMJ Open*
Manuscript ID	bmjopen-2021-053856.R1
Article Type:	Original research
Date Submitted by the Author:	17-Aug-2021
Complete List of Authors:	Ncama, Busisiwe; University of KwaZulu-Natal College of Health Sciences<br>Kuupiel , Desmond ; University of KwaZulu-Natal College of Health Sciences<br>Duma, Sinegugu; University of KwaZulu-Natal College of Health Sciences<br>Mchunu , Gugu ; University of KwaZulu-Natal College of Health Sciences<br>Guga , Phindile ; University of KwaZulu-Natal College of Health Sciences<br>Slotow, Rob; University of KwaZulu-Natal
**Primary Subject Heading**:	Public health
Secondary Subject Heading:	Public health
Keywords:	Public health < INFECTIOUS DISEASES, PUBLIC HEALTH, Health & safety < HEALTH SERVICES ADMINISTRATION & MANAGEMENT

SCHOLARONE™
Manuscripts

I, the Submitting Author has the right to grant and does grant on behalf of all authors of the Work (as defined in the below author licence), an exclusive licence and/or a non-exclusive licence for contributions from authors who are: i) UK Crown employees; ii) where BMJ has agreed a CC-BY licence shall apply, and/or iii) in accordance with the terms applicable for US Federal Government officers or employees acting as part of their official duties; on a worldwide, perpetual, irrevocable, royalty-free basis to BMJ Publishing Group Ltd ("BMJ") its licensees and where the relevant Journal is co-owned by BMJ to the co-owners of the Journal, to publish the Work in this journal and any other BMJ products and to exploit all rights, as set out in our licence.

The Submitting Author accepts and understands that any supply made under these terms is made by BMJ to the Submitting Author unless you are acting as an employee on behalf of your employer or a postgraduate student of an affiliated institution which is paying any applicable article publishing charge ("APC") for Open Access articles. Where the Submitting Author wishes to make the Work available on an Open Access basis (and intends to pay the relevant APC), the terms of reuse of such Open Access shall be governed by a Creative Commons licence – details of these licences and which Creative Commons licence will apply to this Work are set out in our licence referred to above.

Other than as permitted in any relevant BMJ Author's Self Archiving Policies, I confirm this Work has not been accepted for publication elsewhere, is not being considered for publication elsewhere and does not duplicate material already published. I confirm all authors consent to publication of this Work and authorise the granting of this licence.

For peer review only

A Scoping Review of Food Safety at Transport stations in Africa

Busisiwe P. Ncama[1], Desmond Kuupiel[1,2], Sinegugu E. Duma[1], Gugu Mchunu[1], Phindile Guga[1], Rob Slotow[3]

[1]School of Nursing and Public Health, College of Health Sciences, University of KwaZulu-Natal, Durban, South Africa

[2]Research for Sustainable Development Consult, Sunyani, Ghana

[3]School of Life Sciences, College of Agriculture, Engineering and Science, University of Kwazulu-Natal, Pietermaritzburg, South Africa.

Corresponding Author: Desmond Kuupiel (PhD)

Address: 2nd Floor George Campbell Building, Department of Public Health Medicine, School of Nursing and Public Health, University of KwaZulu-Natal, Durban, 4001, South Africa

Email address: desmondkuupiel98@hotmail.com / KuupielD@ukzn.ac.za

Tel: +27735568200/ +233550972968

Email addresses of co-authors

Prof. Busisiwe P. Ncama (Ncamab@ukzn.ac.za)

Prof. Sinegugu E. Duma (DumaS1@ukzn.ac.za)

Prof. Gugu Mchunu (Mchunug@ukzn.ac.za)

Ms. Phindile Guga (phindile.guga@gmail.com)

Prof. Rob Slotow (Slotow@ukzn.ac.za)

For peer review only

For peer review only - http://bmjopen.bmj.com/site/about/guidelines.xhtml

Abstract

Background: The World Health Organization (WHO) has declared food safety as a public health concern. Transport hubs such as taxi ranks, bus stations, and other transport exchange sites are major food trading/purchasing sites, particularly in Africa. Research evidence is needed to improve food safety policies and ensure consumption of safe food, owing to the increasing burden of foodborne diseases, particularly in the WHO Africa Region. Therefore, we systematically mapped and described research evidence on food safety at transport stations in Africa.

Methods: Guided by the Arksey and O'Malley framework, we searched for original research articles in PubMed, Web of Science, and EBSCOhost (Academic search complete, CINAHL with Full-text, and Health Source), SCOPUS, and Google Scholar from their inception to October 2020. We included studies that focused on food safety, involved transport stations, involved African countries, and were published in English. Two investigators independently screened the articles at the abstract and full-text stages in parallel, guided by the eligibility criteria, and then extracted all relevant data. Thematic analysis was used to organise the data into themes and sub-themes, and a narrative summary of the findings is presented.

Results: Of the total 23,852 articles obtained from the database searches, 16 studies published in six countries met the inclusion criteria. These 16 studies were published between 1997 and 2019, with the most (5) in 2014. Of the 16 studies, 43.8% (7) were conducted in South Africa, three studies in Ghana, two in Ethiopia, and one study each in Nigeria, Kenya, Lesotho, and Zambia. Most (44.4%) of the included studies focused on microbial safety of food; few studies (22.2%) focused on hygienic practices, and one study investigated the perspective of consumers or buyers. Microbes detected in the foods samples were *Salmonella* spp, *E. coli*, *Shigella* spp, *Bacillus* sp, *Staphylococcus aureus*, which resulted mainly from poor hygiene practices.

Discussion: There is limited research that focused on food safety at transport stations in Africa, especially on aspects such as hygiene practices, food storage, and occupational health and food safety. Therefore, we recommend more research in these areas, using various primary study designs, to inform and improve food safety policies and practices for transport stations in African countries alongside improving access to clean water/handwashing facilities, and undertaking structural changes to facilitate behaviours and monitoring for unintended consequences such as livelihoods of vulnerable populations.

For peer review only - http://bmjopen.bmj.com/site/about/guidelines.xhtml

Funding: Funding for this work was provided by the Sustainable and Healthy Food Systems (SHEFS) Programme.

Article summary

Strengths and limitations of this study

- To the best of our knowledge, this is the first scoping review to systematically explore literature and describe research evidence on food safety at transport stations as well as identify gaps for future research in Africa.
- This scoping review's evidence sources were searched using a systematic approach, and duplicate screening.
- This review is limited to Africa as well as English language publications.

For peer review only - http://bmjopen.bmj.com/site/about/guidelines.xhtml

Background

The World Health Organisation (WHO) estimates that more than 600 million people fall sick (almost 1 in 10 people) with foodborne diseases annually, of which nearly 420 000 people die, and about 33 million years of healthy lives are lost every year worldwide [1, 2]. The burden of foodborne diseases is estimated to be highest in the WHO African and South-East Asia Regions, mainly occurring among vulnerable populations such as infants, young children, pregnant women, older people, poor people, and individuals with underlying illnesses [3]. Food contamination mostly results throughout the food supply chain (from the procedures used in processing the foods, inadequate storage temperatures, unhygienic practices by food handlers, poor sanitation at cooking places/vending areas, poor waste management, and inadequate treatment of leftovers) [4].

Unsafe food has negative implications on health systems, and affects the development and national economies of countries, as well as trade [3]. Therefore, eating unsafe foods poses a significant public health threat. To avert the consequences of unsafe food on health systems, and to sustain national economies, development, trade, and tourism [5, 6], the WHO in 2006 declared food safety as a global public health concern [7, 8]. “Food safety refers to routines in the preparation, handling and storage of food meant to prevent foodborne illness and injury” [5]. To reduce the incidence of food-related diseases, particularly in high burden regions, the observations of food safety measures/precautions at all levels of the food processing chain, including the places where food is prepared and sold, are critical [9, 10].

Like other WHO Regions, especially in low-and-middle-income countries, food trading in the Africa Region takes place at several formal and informal places, such as in the markets, restaurants, streets, open spaces in academic institutions, and transport stations (taxi ranks, bus stations, lorry parks), and other transport exchange sites. Food vending at public spaces serves as a source of livelihood [6, 10, 11], and more than two billion people eat food sold at various vending locations. including transportations stations on daily basis globally [12, 13]. To this end, evidence is essential to inform in-country policies/guidelines, and further research, to ensure that food prepared and sold at transport stations promotes livelihoods, nutrition, food safety, and environmentally sustainable practices. This scoping review systematically mapped literature focused on food safety at transport stations in Africa, to summarise evidence and identify gaps.

For peer review only - http://bmjopen.bmj.com/site/about/guidelines.xhtml

Methods

Scope of the review

The Arksey & O'Malley framework (research question identification; identifying relevant studies; selection of study; data charting, collating, and summarising and reporting the findings [14, 15]) was employed to scope and synthesise literature to answer the question - what evidence exists on food safety at transport stations in Africa? This review's study protocol was developed *a priori* [16]. This study included published peer-reviewed articles that reported findings from any African country/countries, focused on food safety, and involved transport stations. However, this study was limited to English publications (due to lack of expertise in other international languages), and primary study designs [16]. A detailed description of this scoping review study eligibility criteria is captured in the published protocol [16]. We followed the Preferred Reporting Items for Systematic and Meta-analysis (PRISMA) extension for Scoping Reviews checklist to report this study [17].

Identify relevant studies

We searched for primary research articles relating to food safety at transport stations in PubMed, Web of Science, and EBSCOhost (Academic search complete, CINAHL with Full-text, and Health Source), SCOPUS, and Google Scholar from their inception to October 2020. To enable the capturing of all relevant articles, a comprehensive search strategy (developed in consultation with an expert librarian) consisting of keywords, Boolean terms (AND/OR), and Medical Subject Heading terms, was used for the electronic database search (Supplementary file 1). Syntax was modified appropriately where needed. Filters such as date and study design were not applied during the literature search in the databases. DK and PG independently conducted the database search and title screening, and imported all potentially eligible articles onto an EndNote Library. The reference lists of all included articles were also screened for potentially relevant articles using the same approach.

Selection of articles

Prior to the abstract screening, the 'find duplicates' function in EndNote was used to find all duplicate articles, and they were removed from the library. A screening form was developed in Google forms, using this study's eligibility criteria, for the abstract and full text screening phases. Two reviewers (co-authors) independently screened the abstracts as well as the full text articles. Discrepancies that arose during the abstract stage were resolved by discussion among

For peer review only - http://bmjopen.bmj.com/site/about/guidelines.xhtml

the review team until a consensus was reached. At the full text screening phase, discrepancies were resolved by a third reviewer. All the additional articles identified from the reference list of the included articles equally underwent full text assessment. The PRISMA flow diagram was employed to account for all the articles involved [18].

Charting the data

A data extraction form was designed consisting of the following: Author(s) and publication details, country of study, study design, study setting, study population, sample size, sex, study findings, and recommendations. To ensure consistency and reliability, two reviewers piloted the data extraction sheet using a random sample of three included studies. The pilot testing of the form also enabled the review team to discuss discrepancies, and to revise the data extraction form prior to its final usage. Subsequently, two reviewers conducted the data extraction for the remaining 15 included studies using both inductive and deductive approaches. The review team resolved all discrepancies at this stage through discussion.

Collating, summarizing, and reporting the results

This study subsequently employed thematic analysis, and collated all the emerging themes and sub-themes relating to food safety. A summary of the findings from the included studies is presented narratively.

Patient and Public Involvement

No patient involved

Results

Of the 23,852 articles obtained from the database searches (see Figure 1 flow diagram), 146 articles met the eligibility criteria at the title screening stage. Using EndNote “Find Duplicates” function, 30 duplicates were found and removed before abstract screening was conducted. Subsequently, 83 articles were removed at the abstract screening, and 20 at full text (17 of these did not include transport stations/taxi ranks/bus stations, but did involve sale from market centres, public places, chop bars, mini restaurants, major streets, and sidewalks, and were excluded). Finally, 13 studies were included, and, from a manual search of their reference lists, a further three articles were added, giving a total of 16 articles for further analysis.

For peer review only - http://bmjopen.bmj.com/site/about/guidelines.xhtml

Characteristics of the included studies

Table 1 presents a summary of the characteristics of the included studies. Of the 16 included studies, about fourty-four (43.8%) were conducted in South Africa [19-25], three (18.8%) in Ghana [4, 26, 27], two (12.5%) in Ethiopia [28, 29], and one (6.2%) each in Nigeria [30], Kenya [31], Lesotho [32], and Zambia [33]. Most of the studies were published in the last six years, however, no published study was found in 2015 and 2020 (Figure 2). Fifteen (93.8%) of the included studies were cross-sectional studies, and one (6.2%) was a mixed-method study. Of the 16 included studies, 50.0% reported on microbial safety of food [4, 20, 23, 27-29, 33, 34], and 25.0% reported hygiene practices of food handlers/vendors [6, 21, 30, 31]. One included study each reported on the following: occupational health and food safety risk [24]; knowledge of hygiene practice [26]; hygiene practices of food handlers/vendors and microbial safety [25]; and knowledge of food safety measures and hygiene practice by food handlers/vendors [32].

Findings from the included studies

Microbial safety of food

Of the nine included studies that reported findings on microbial safety of food, 44.4% were conducted in South Africa [19, 20, 23, 25], 22.2% each in Ghana [4, 27] and Ethiopia [28, 29], and the last 11.1% in Zambia [33]. Seven of the eight studies reported unacceptable levels of microbes in the food [4, 20, 23, 27-29, 33, 34]. Table 2 presents a summary of the key findings as well as the sample type, analytic approach, and the microbes reported.

Hygiene practices of food handlers/vendors

Food preparation

Of the 16 included studies, eight reported research findings relating to food preparation. Fifty percent of these eight studies were from South Africa [6, 21, 23, 25], and the remainder were from Ghana [26], Nigeria [30], Kenya[31], and Lesotho [32]. The studies in South Africa focused on the following: hygiene practices and implications for consumers [21]; food and nutrition knowledge as well as practices related to food preparation [6], the effect of hygiene practices and attitudes of meat vendors [25], and sources of food contamination [23]. The study from Ghana investigated how fast food operators washed their hands [26], whilst the studies from Nigeria, Kenya, and Lesotho evaluated food safety and sanitary practices [30]; food

For peer review only - http://bmjopen.bmj.com/site/about/guidelines.xhtml

vendors and hygiene practices [31]; and food safety knowledge, attitudes and practices of food vendors and consumers' perceptions [32]. A summary of the key findings from these studies is presented below (Table 3).

Knowledge of hygiene practices/food safety precautions

In Ghana, McArthur-Floyd et. al. study [26], revealed that the majority (94%) of fast food operators knew food safety precautions [26]. Letuka et. al. study [32] in Lesotho, indicated that 95% of food vendors did not know washing utensils with detergents helps reduce contamination [32]. The mean knowledge (49%±11) of the food vendors included in the study was considered poor [32]. About 6% of the consumers that participated in the study chose not to buy food sold at taxi ranks due to food safety issues and hygiene [32].

Occupational health and food safety risk

In South Africa, Qekwana et.al. [24]evaluated the occupational health and food safety risks associated with the traditional slaughter of goats, and the consumption of such meat [24]. Approximately 63% of the practitioners were not wearing protective clothing during slaughter, and about 78% of practitioners did not know their health status [24]. Almost 83% of the practitioners hung up their carcass to facilitate bleeding, flaying, and evisceration [24]. The study further observed that none of the practitioners practiced meat inspection [24]. In Nigeria, Aluko et al. [30] study revealed that approximately 62% of the vendors had no formal training, and their medical status was also unknown [30].

Discussion

This scoping review mapped evidence on food safety at transport stations in Africa, and revealed a very low number of papers that are published in this area, given many African employees in both formal and informal sectors commute through these transport hubs [12, 13]. An average of one paper per year relating to food safety at transport hubs in Africa as revealed by this review is simply not enough. Nonetheless, the few papers depict an imbalance of research, with most focused on microbial safety [4, 20, 23, 27-29, 33, 34], and few on socio-economic aspects such as hygiene practices [6, 21, 30, 31], and occupational health and food safety risk [24]. Moreover, this review revealed no study evaluated the storage of food or how the food is transported to the vending site.

For peer review only - http://bmjopen.bmj.com/site/about/guidelines.xhtml

As evidence by this review, most of the food sold at transport hubs does not meet the minimum standards and is not safe for consumption due to the presence of several microbes [4, 19, 23, 25, 27, 29, 33, 35]. There are several reasons for this such as poor practices relating to hygiene, storage, preparation, cooking, cleaning, and serving [4, 20, 23, 27-29, 33, 34]. However, these findings are similar to previous review findings involving markets [36], homes and restaurants [37]. A recent publication by Gizaw [36] indicated that several studies reported microbial contamination of foods sold in the market, with bacteria and fungi similar to those identified in our review [36]. Also, a review by the WHO reported that the main factors contributing to foodborne disease outbreaks in homes or restaurants were poor temperature control in preparing, cooking, and storing food [37]. Although very few papers were found by this review, the evidence is compelling that there should be policy interventions to address issues relating poor hygiene practices, including food storage, preparation, cooking, cleaning, and serving by food handlers at transport hubs, not only in South Africa but across Africa.

Similar to a previous scoping review [38], most of the included papers were published within the last six years but, no published study was found in 2015 and 2020. Whilst the reason for the lack of published papers in 2015 might be difficult to determine, the COVID-19 pandemic which resulted in "covidisation" of research might be the reason for the lack of publication in this field of research in 2020. Although we cannot conclude that no primary research has been conducted in these countries focusing on the safety of food sold at transport stations, it suggests a research/publication gap. Food safety research is, perhaps, more relevant now than ever in Africa, since the burden of foodborne diseases is rising annually, resulting in the declaration of food safety as a public health concern by the WHO [7, 8]. Aside from this, most commuters tend to buy ready-to-eat (RTE) food from street food vendors, including those at transport hubs [39, 40]; hence, the sale of food at transport stations is rising [40, 41], particularly in Africa [6] partly due to an increase in demand for RTE, and the employment opportunities it offers to many individuals who otherwise would not have had any source of income [38, 42]. Even more worrying is the fact that most of the articles included that focused on microbial safety, reported high levels of food contamination with several microorganisms, especially *Salmonella* spp and *E. coli* [4, 19, 23, 25, 27, 29, 33, 35]. Therefore, more research is needed across African countries to prevent potential negative consequences.

Our study findings have implications for practice and research. For instance, the likelihood of food poisoning with microbes such as *Salmonella* spp, *E. coli., Shigella* spp*, Bacillus* spp*, S. aureus,* and several others, revealed by most of the included studies that focused on microbial

For peer review only - http://bmjopen.bmj.com/site/about/guidelines.xhtml

contamination of food, is alarming. This, if not checked, could further worsen the already high burden of foodborne diseases in a continent that has several of its countries already experiencing many health systems and economic challenges. Aside from this, the majority of individuals who commute through transport hubs, possible will purchase a meal from a transport hub/exchanges site, which may be the only meal of the day [12, 13], and yet the food safety standards are poor [4, 20, 23, 27-29, 33, 34]. Thus, if not checked, the excess cases of foodborne diseases from any outbreak will further impact negatively on the already challenged public health systems in Africa. Also, poor people who are exposed to these unsafe foods get an infection, may have to pay more for healthcare, which can further exacerbate their poverty situation. Moreover, people who are already living in extreme poverty who get exposed to foodborne disease may not even make it to the hospital for care and can end up dying at home [43].

Good hygiene and sanitation practices, such as adequate hand washing, adequate washing and storage of pots and dishes, good waste management, observation of food preparation standards and serving etiquette, among others, have the potential to reduce the risk of food contamination from both biological and non-biological hazards, yet this study reveals fewer studies that focused on hygienic practices. We, therefore, recommend more research to further inform contextualised policy decisions aimed at improving hygiene and sanitation practices by food vendors at transport stations. Also, very relevant to ensuring food safety is the occupational health practices of the vendors. Regular food handling tests and food inspections, conducted by the appropriate local authorities, should be mandatory in all African countries. Food handler tests should seek to ensure that food vendors are fit healthwise to prepare and serve food meant for public consumption. However, our review found limited studies that evaluated occupational health and food safety. Considering that evidence from South Africa and Nigeria suggests about 78% and 62% of food vendors do not know their health status [30, 44], and the increasing number of informal food sellers at various transport exchange sites, future studies are recommended to focus on occupational health and food safety in Africa. The means and manner of storing food, especially leftover RTE food, can either increase or reduce the risk of food contamination, but, again, this scoping review found no study that focused on food storage practices of the vendors at transport stations. Also essential, and yet we did not find any study focusing on it, is the quality of food (nutritious aspects) of the meals sold at transport stations. Eating a well nourishing diet or balanced meals is critical to ensure good health [45-47], hence, we encourage future primary studies to include the nutritious aspects. Such studies may help

For peer review only

streamline guidelines or inform policies to improve the quality of the food sold at transport exchange sites or taxi ranks. Moreover, this review found that the majority (17 out of 18) of the respondents in the included studies were the vendors (mostly females) or food samples taken from the vendors. The perspectives of consumers (buyers) or commuters regarding food safety at transport stations are also very relevant, and we recommend future research to involve them. A comparative study to investigate food safety practices among males and females food vendors at transport stations might be relevant since many males are now getting involved in the business [6, 48, 49].

To the best of our knowledge, this study is the first scoping review that systematically mapped literature relating to food safety at transport stations in Africa. A major strength of our study method is that it permits the inclusion of multiple study designs. Also, the choice of this study method permitted us to highlight literature gaps, and made recommendations for future research. Aside from this, we conducted a thorough search in six databases using a comprehensive search strategy which enabled us to capture the most relevant articles to answer the review question. Moreover, two independent reviewers were used to select the studies and perform data extraction processes which helped to prevent selection bias and ensured the reliability and trustworthiness of this study results. Despite this, our scoping review has many limitations. This study included only original study peer reviewed papers, which resulted in the exclusion of one review paper [38], and one Masters' dissertation [50]. We did not also consult the websites of WHO and the Food and Agriculture Organisation websites for possible relevant studies. Furthermore, this study cannot be generalised since the search was limited to African countries only. Although date limitation was removed, we limited the publication language to English only, which perhaps eliminated relevant articles published in other languages. Despite these limitations, this study has provided essential evidence relating to food safety at transport stations and has shown literature gaps to guide future research.

Conclusion

Based on this scoping review's eligibility criteria, our study results suggest there is limited research focusing on food safety at transport stations in Africa. Most of the existing published studies are focused on microbial safety of food, and very few/none on other aspects such as hygiene practices, food storage, occupational health and food safety, and nutrition. Hence, we recommend more primary research involving community members and policy makers in these areas going forward alongside improving access to clean water/handwashing facilities, and

For peer review only - http://bmjopen.bmj.com/site/about/guidelines.xhtml

undertaking structural changes to facilitate behaviours and monitoring for unintended consequences such as livelihoods of vulnerable populations.

Abbreviations

PRISMA- Preferred Reporting Items for Systematic and Meta-analysis

RTE- Ready-to-eat

WHO- World Health Organization

Ethics and dissemination

Not required. This study did not include human participants.

Data availability statement

Not applicable. All sources of data have been adequately referenced

Patient and Public Involvement

No patient involved

Statements

Acknowledgments

Support for this work was provided by the Sustainable and Healthy Food Systems (SHEFS) Programme, supported through the Wellcome Trust's Our Planet, Our Health Programme

Authors' contributions

BPN, DK, SED, SM, and RS conceptualized and designed the study. DK developed and designed the database search strategy and conducted the search. PG contributed to the screening of the studies and data extraction. DK wrote the draft manuscript and BPN, SED, GM, and RS critically review it and made revisions. All the authors approved the final version of the manuscript.

Funding

Funding for this work was provided by the Sustainable and Healthy Food Systems (SHEFS) Programme, supported through the Wellcome Trust's Our Planet, Our Health Programme [grant number: 205200/Z/16/Z]. The funder played no role in the literature search and writing of the manuscript.

For peer review only - http://bmjopen.bmj.com/site/about/guidelines.xhtml

Competing interests

None declared

References

1. The Lancet Gastroenterology H. Food safety really is everyone's business. The lancet Gastroenterology & hepatology. 2019;4(8):571.

2. World Health Organization. Estimating the burden of foodborne diseases Geneva: World Health Organization; 2015 [cited 2019 25/10/2019]. Available from: https://www.who.int/activities/estimating-the-burden-of-foodborne-diseases.

3. World Health Organization. Food Safety Geneva: World Health Organization; 2016 [cited 2020 17/11/2020]. Available from: https://www.afro.who.int/health-topics/food-safety.

4. Feglo P, Sakyi K. Bacterial contamination of street vending food in Kumasi, Ghana. Journal of Medical Biomedical Sciences. 2012;1(1):1-8.

5. Organization WH. Food Safety: What you should know Geneva: World Health Organization; 2015 [cited 2019 25/10/2019]. Available from: http://www.searo.who.int/entity/world_health_day/2015/whd-what-you-should-know/en/.

6. Hill J, Mchiza Z, Puoane T, Steyn NP. The development of an evidence-based street food vending model within a socioecological framework: a guide for African countries. PloS one. 2019;14(10):e0223535.

7. World Health Organization. Five keys to safer food manual Geneva: World Health Organization; 2006 [cited 2019 25/10/2019]. Available from: https://www.who.int/foodsafety/publications/consumer/manual_keys.pdf.

8. Lamin-Boima P. Knowledge, Attitude And Practice Of Street Food Vendors In Selected Schools Within Bo City Southern Sierra Leone.

9. Lues JFR, Rasephei MR, Venter P, Theron MM. Assessing food safety and associated food handling practices in street food vending. International Journal of Environmental Health Research. 2006;16(5):319-28.

10. Abrahale K, Sousa S, Albuquerque G, Padrao P, Lunet N. Street food research worldwide: a scoping review. Journal of human nutrition and dietetics : the official journal of the British Dietetic Association. 2019;32(2):152-74.

For peer review only - http://bmjopen.bmj.com/site/about/guidelines.xhtml

11. Liu Z, Zhang G, Zhang X. Urban street foods in Shijiazhuang city, China: Current status, safety practices and risk mitigating strategies. Food Control. 2014;41:212-8.

12. Food and Agriculture Organization. Selling street and snack foods Rome: Food and Agriculture Organization; 2011 [cited 2019 25/10/2019]. Available from: http://www.fao.org/3/a-i2474e.pdf.

13. Gelormini M, Damasceno A, Lopes SA, Malo S, Chongole C, Muholove P, et al. Street Food Environment in Maputo (STOOD Map): a Cross-Sectional Study in Mozambique. JMIR research protocols. 2015;4(3):e98.

14. Arksey H, O'Malley L. Scoping studies: towards a methodological framework. International Journal of Social Research Methodology. 2005;8(1):19-32.

15. Levac D, Colquhoun H, O'Brien KK. Scoping studies: advancing the methodology. Implementation Science. 2010;5(1):69.

16. Ncama BP, Kuupiel D, Duma SE, McHunu G, Guga P, Slotow R. Mapping evidence of food safety at transport stations in Africa: a scoping review protocol. BMJ Open. 2020;10(8):e035879-e.

17. Tricco AC, Lillie E, Zarin W, O'Brien KK, Colquhoun H, Levac D, et al. PRISMA extension for scoping reviews (PRISMA-ScR): checklist and explanation. Annals of internal medicine. 2018;169(7):467-73.

18. Moher D, Liberati A, Tetzlaff J, Altman DG, The PRISMA Group. PRISMA 2009 Flow Diagram. 2009;6(2009):1000097.

19. Oguttu JW, McCrindle CME, Makita K, Grace D. Investigation of the food value chain of ready-to-eat chicken and the associated risk for staphylococcal food poisoning in Tshwane Metropole, South Africa. Food Control. 2014;45:87-94.

20. Mafune TS, Takalani TK, Anyasi TA, Ramashia SE. Microbial Safety of Street Vended Foods Sold in Thohoyandou, South Africa. Journal of Human Ecology. 2016;53(3):205-12.

21. Kok R, Balkaran R. Street food vending and hygiene practices and implications for consumers. Journal of Economics Behavioral Studies. 2014;6(3):188-93.

22. Hill J, McHiza Z, Puoane T, Steyn NP. Food sold by street-food vendors in Cape Town and surrounding areas: a focus on food and nutrition knowledge as well as practices related to

For peer review only - http://bmjopen.bmj.com/site/about/guidelines.xhtml

food preparation of street-food vendors. Journal of Hunger & Environmental Nutrition. 2019;14(3):401-15.

23. Mazizi B, Muchenje V, Makepe M, Mutero G. Assessment of Aerobic Plate Counts, Staphylococcus aureus, Escherichia coli and Salmonella in Meat Sold by Street Vendors in the Eastern Cape Province, South Africa. Journal of Food Nutrition Research. 2017;5(6):436-42.

24. Qekwana DN, McCrindle CM, Oguttu JW, Grace D. Assessment of the occupational health and food safety risks associated with the traditional slaughter and consumption of goats in Gauteng, South Africa. International journal of environmental research and public health. 2017;14(4):420.

25. Tshipamba M, Lubanza N, Adetunji M, Mwanza MJJFMSH. Evaluation of the Effect of Hygiene Practices and Attitudes on the Microbial Quality of Street Vended Meats Sold in Johannesburg, South-Africa. 2018;3(137):2476-059.1000137.

26. McArthur-Floyd M, Commey V, Boakye NAB. Evaluation of Food Safety among Fast Food Operators in Madina, Accra. Evaluation. 2016;54.

27. Abakari G, Cobbina SJ, Yeleliere E. Microbial quality of ready-to-eat vegetable salads vended in the central business district of tamale, Ghana. International Journal of Food Contamination. 2018;5(1).

28. Kibret M, Tadesse M. The bacteriological safety and antimicrobial susceptibility of bacteria isolated from street-vended white lupin (Lupinus albus) in Bahir Dar, Ethiopia. Ethiopian Journal of Health Sciences. 2013;23(1):19-26.

29. Eromo T, Tassew H, Daka D, Kibru G. Bacteriological quality of street foods and antimicrobial resistance of isolates in Hawassa, Ethiopia. Ethiopian journal of health sciences. 2016;26(6):533-42.

30. Aluko OO, Ojeremi TT, Olaleke DA, Ajidagba EB. Evaluation of food safety and sanitary practices among food vendors at car parks in Ile Ife, southwestern Nigeria. Food Control. 2014;40:165-71.

31. Odundo A, Okemo P, Chege P. An Assessment of Food Safety Practices among Street Vendors in Mombasa, Kenya. International Journal of Health Sciences Research. 2018;8(5):235-43.

For peer review only

32. Letuka P, Nkhebenyane J, Thekisoe O. Assessment of Food Safety Knowledge, Attitudes and Practices among Street Food Vendors and Consumers' Perceptions of Street Food Vending in Maseru Lesotho. 2019.

33. Jermini M, Bryan FL, Schmitt R, Mwandwe C, Mwenya J, Zyuulu MH, et al. Hazards and Critical Control Points of Food Vending Operations in a City in Zambia. J Food Prot. 1997;60(3):288-99.

34. Oguttu JW, McCrindle CM, Makita K, Grace D. Investigation of the food value chain of ready-to-eat chicken and the associated risk for staphylococcal food poisoning in Tshwane Metropole, South Africa. Food Control. 2014;45:87-94.

35. Kibret M, Tadesse MJEjohs. The bacteriological safety and antimicrobial susceptibility of bacteria isolated from street-vended white lupin (Lupinus albus) in Bahir Dar, Ethiopia. 2013;23(1):19-26.

36. Gizaw Z. Public health risks related to food safety issues in the food market: a systematic literature review. Environmental Health and Preventive Medicine. 2019;24(1):68.

37. Todd EC. Epidemiology of foodborne diseases: a worldwide review. World health statistics quarterly Rapport trimestriel de statistiques sanitaires mondiales. 1997;50(1-2):30-50.

38. Abrahale K, Sousa S, Albuquerque G, Padrao P, Lunet N. Street food research worldwide: a scoping review. Journal of Human Nutrition and Dietetics. 2019;32(2):152-74.

39. Bellia C, Pilato M, Seraphin H. Street food and food safety: a driver for tourism? Calitatea. 2016;17(S1):20.

40. Mosupye FM, Von Holy A. Microbiological hazard identification and exposure assessment of street food vending in Johannesburg, South Africa. International Journal of Food Microbiology

2000;61(2-3):137-45.

41. Mosupye FM, von Holy A. Microbiological quality and safety of ready-to-eat street-vended foods in Johannesburg, South Africa. International Journal of Food Microbiology. 1999;62(11):1278-84.

For peer review only - http://bmjopen.bmj.com/site/about/guidelines.xhtml

42. Muzaffar AT, Huq I, Mallik BA. Entrepreneurs of the streets: An analytical work on the street food vendors of Dhaka City. International journal of Business Management. 2009;4(2):80-8.

43. World Health Organisation. Universal Health Coverage Geneva2020 [cited 2021 09/04/2021]. Available from: https://www.who.int/health-topics/universal-health-coverage#tab=tab_1.

44. Qekwana DN, McCrindle CM, Oguttu JW, Grace D. Assessment of the occupational health and food safety risks associated with the traditional slaughter and consumption of goats in Gauteng, South Africa. International Journal of Environmental Research. 2017;14(4):420.

45. Melanson K. Nutrition review: lifestyle approaches to promoting healthy eating for children. American journal of lifestyle medicine. 2008;2(1):26-9.

46. Carrier N, Villalon L, Lengyel C, Slaughter SE, Duizer L, Morrison-Koechl J, et al. Diet quality is associated with malnutrition and low calf circumference in Canadian long-term care residents. BMC nutrition. 2019;5:57.

47. Freeland-Graves JH, Nitzke S. Position of the academy of nutrition and dietetics: total diet approach to healthy eating. Journal of the Academy of Nutrition Dietetics. 2013;113(2):307-17.

48. Okojie P, Isah E. Sanitary conditions of food vending sites and food handling practices of street food vendors in Benin City, Nigeria: implication for food hygiene and safety. Journal of environmental public Health Reports. 2014;2014.

49. da Silva SA, Cardoso RdCV, Góes JÂW, Santos JN, Ramos FP, de Jesus RB, et al. Street food on the coast of Salvador, Bahia, Brazil: A study from the socioeconomic and food safety perspectives. Food control. 2014;40:78-84.

50. Acheampong BE. Assessment of food hygiene practices by street food vendors and microbial quality of selected foods sold. A Study at Dunkwa-On-Offin, Upper Denkyira East Municipality of the Central Region 2015.

For peer review only - http://bmjopen.bmj.com/site/about/guidelines.xhtml

Table 1: Characteristic of the included sources of evidence

**Author, year**	**Country**	**City/Town**	**Study design**	**Study setting**	**Study population**	**Sample size**	**Sex of vendors**	**Outcome reported**
Oguttu et. al., 2014 [34]	South Africa	Tshwane Metropole, Gauteng Province	Mixed-methods study	Taxi rank	Vendors selling Ready–to-eat chicken	100 samples of Ready–to-eat chicken	Females	Microbial safety of food
Mafune et. al., 2016 [20]	South Africa	Thohoyandou, Limpopo Province	Cross-sectional study	Taxi rank, bus station, shopping mall, and street stalls	Food samples from street vendors	28 samples	Not specified	Microbial safety of food
Kibret et. al., 2013 [28]	Ethiopia	Bahir Dar Town	Cross-sectional study	Main roads sites, bus station, groceries, taxi ranks	Ready–to-eat white lupin sample from vendors	40 samples (200 grams of white lupin)	Not specified	Microbial safety of food
Abakari et. al., 2018 [27]	Ghana	Tamale, Northern Region	Cross-sectional study	Taxi rank, bus stops, transport yard, and timber Market	Ready-to-eat salad samples from food vendors	30 salad samples	Not specified	Microbial safety of food
Aluko et.al., 2014 [30]	Nigeria	Ile Ife, southwestern Nigeria	Cross-sectional study	Car parks	Food vendors	160 (117 stationery and 43 mobile vendors)	Males and females	Hygiene practices of food handlers/vendors
Odundo et. al., 2018 [31]	Kenya		Cross-sectional study	Major bus stops, markets, shopping areas, construction	Food vendors	130	Males and females	Hygiene practices of food handlers/vendors

For peer review only

				sites, and commercial areas				
Kok et. al., 2014 [21]	South Africa	Durban, KwaZulu-Natal Province	Cross-sectional study	Transport exchange site	Food vendors	29	Not specified	Hygiene practices of food handlers/vendors
Letuka et. al., 2019 [32]	Lesotho	Maseru	Cross-sectional study	Taxi ranks	Food vendors	141 (48 food handlers and 93 consumers)	Male and female	Knowledge of food safety measures and hygiene practice by food handlers/vendors
Eromo et. al., 2016 [29]	Ethiopia	Hawassa City	Cross-sectional study	Bus station	Food samples from street food vendors	72 samples from six food items	Not specified	Microbial safety of food
McArthur-Floyd et. al., 2016 [26]	Ghana	Madina (Accra), Greater Accra Region	Cross-sectional study	Taxi rank, and transport exchange sites	Food vendors	200	Males and females	Knowledge of hygiene practice
Hill et. al., 2019 [6]	South Africa	Cape Town	Cross-sectional study	Train, bus stations, and taxi ranks, community centers, market	Food vendors	831	Males and females	Hygiene practices of food handlers/vendors
Mazizi et. al., 2017 [23]	South Africa	Alice (Nkonkobe) and King William's Town (Buffalo City), Eastern Cape province	Cross-sectional study	Taxi rank and bus stations	Street food vendors	136 food samples-cooked and raw.	Not specified	Microbial safety of food

For peer review only - http://bmjopen.bmj.com/site/about/guidelines.xhtml

Qekwana et.al, 2017 [24]	South Africa	Tshwane Metropolitan Municipality, Gauteng Province	Cross-sectional study	Taxi ranks and Informal markets	Traditional goat slaughter	105 people	Males and females	Occupational health and food safety risk
Flego et. al., 2012 [4]	Ghana	Kumasi, Ashanti Region	Cross-sectional study	Bus terminals	Food samples from vendors	60 food samples	Not specified	Microbial safety of food
Tshipamba et. al., 2018 [25]	South Africa	Johannesburg	Cross-sectional study	Taxi ranks and streets	Meat samples from vendors	115 meat samples	Not specified	Hygiene practices of food handlers/vendors, and microbial safety of food
Jermini et. al., 1997 [33]	Zambia	Not specified	Cross-sectional study	bus park/station and large market	Samples of raw, processed, and cooked Foods from street food vendors	Not specified	Not specified	Microbial safety of food

524

525 **Table 2:** Microbial safety of food at transport stations

Study	Type of sample	Analytical approach	Microbes reported	Key results	Key conclusion
Oguttu et. al. [34]	Ready-to-eat (RTE) chicken	3M™ Petrifilm™ plates	*S. aureus*	• High prevalence of *S. aureus* in the sample was (44%; 90% CI: 36.1%-52.2%), with mean *S. aureus* counts of $10^{3.6}$ (90%CI: $10^{3.3}$-$10^{3.9}$).	To reduce the levels of concentration of *S. aureus* on the RTE chicken and promote the sale of safer and

For peer review only - http://bmjopen.bmj.com/site/about/guidelines.xhtml

				• The likelihood of food poisoning with *S. aureus* from RTE chicken was estimated to be 1.3% (90% CI: 0% -2.7%)	affordable RTE chicken for the large urban poor population in South Africa, training of RTE chicken vendors on hygiene is still needed.
Mafune et. al. [20]	Unfermented porridge, boiled cabbage and carrots, boiled peanuts, salad, potato chips, traditional mageu, and stewed beef and grilled chicken	Standard microbiological method	*S. aureus*	• *S. aureus* was <2.4771 log10 cfu/g in all samples and places.<br>• Except for fried potato chips, microbial contamination was observed in the remaining food samples using the total plate count method.	Most of the vended foods investigated met the microbiological standard of RTE foods
Mazizi et. al. [23]	Cooked and raw beef, pork, and mutton samples, surface contact plates, and water samples	Biochemical tests according to international standards methods	*S. aureus, E. coli*, and *Salmonella spp.*	• Mean score of raw beef, mutton, and pork were aerobic plate counts (4.8, 3.7 and 2.8 Log (cfu/g)), *S. aureus* (3.3, 3.7 and 2.8 Log cfu/g), and *E. coli* (1.0, 0.6 and 0.3 Log cfu/g) respectively.	The levels of contamination in cooked meat were lower when compared to the standards set by Commission Regulation for determining the microbiological quality of RTE foods.
Tshipamba et. al. [25]	RTE meat	Standard biochemical and Molecular methods	*Bacillus thuringienis, Bacillus spp., Bacillus subtilis, Bacillus cereus, Citrobacter spp., Enterococcus faecium,*	• Overall mean total bacteria in the samples ranged from 4.3-6.03 cfu/ml × 102 and coliform counts ranged from 1.60-1.95 × 102 cfu/ml<br>• Of the 15 microbes identified, S. aureus occurred in all the meat types and the percentage of occurrence was chicken meat (14%), beef head	Consumers RTE meat are at risk of food borne diseases due to poor hygiene practices of the vendors.

For peer review only - http://bmjopen.bmj.com/site/about/guidelines.xhtml

			Enterococcus faecalis, Kurthia spp., Lysinibacillus spp. Macrococcus caseolyticus, Planomicrobium glaciei, Planococcus antarcticus, S. aureus, S. equorum, and *S. vitulinus*	(43%), beef intestine (50%), and wors (sausage) (20%)	
Kibret et. al. [28]	White lupin	Standard bacteriological techniques, and Kirby-Bauer disk diffusion method for antimicrobial susceptibility test	*E. coli, Salmonella spp, and Shigella spp.*	• Prevalence of bacteria total coliform counts were 954.2±385 at the surface and 756.2±447.3 at the core of white lupin.<br>• Pathogens isolated were as follows *E. coli* 29 (72.5%), *Salmonella* spp. 23 (57.5%) and *Shigella* spp. 8 (20%).<br>• Overall multiple antimicrobial resistances rate was 75%	Contamination of white lupin and a potential health risk to consumers revealed, and the bacteria isolated showed high rates of multiple drug resistance.
Eromo et. al. [29]	Local bread ('ambasha' and'kita'),	Standard microbiological techniques	*E. scoli, Salmonella spp.*, and *S. aureus*	• The microbiological quality in nearly 31% of RTE food samples was beyond the acceptable limits.	Considerable rate of contamination in the foods confirmed. The identified foodborne bacteria and antibiotic resistance isolates could pose a

For peer review only - http://bmjopen.bmj.com/site/about/guidelines.xhtml

	raw fish, chilli ('awaze'), avocado, and cooked potato			• Total colony counts detected ranged from 1.7x105 to 6.7x106 cfu/g.<br>• *E.coli* (29.6%), *Salmonella* spp. (12.7%, and *S. aureus* (9.9%) were the most frequent isolates.<br>• All isolates were 100% sensitive to ciprofloxacin, but 89% of *Salmonella* spp. was resistant to chloramphenicol, 14.3% of *S.aureus* was resistant to vancomycin	public health problem in the study location.
Abakari et. al. [27]	Pre-cut vegetable salads	Standard microbiological methods	*E. coli, Bacillus cereus, Salmonella spp, and Shigella spp.*	• *E. coli* levels ranged from 0 to 7.56 log10 cfu/g; *Bacillus cereus* levels ranged from 0 to 7.44 log10 cfu/g; *Salmonella* spp. ranged from 0 to 4.54 log10 cfu/g, and *Shigella* spp. ranged from 5.54 log10 cfu/g were detected in 96.7%, 93.3%, 73.3%, and 76.7% of the salads samples, respectively.	Salads were revealed to be unwholesome for human consumption and could be deleterious to the health of consumers.
Flego et. al. [4]	RTE foods (ice-kenkey (15), cocoa drink (15), fufu (5), ready-to-eat red pepper for kenkey) (5), salad (10), and macaroni (10))	Standard microbiological methods	*Staphylococci*, *Bacillus* spp., *Klebsiella pneumoniae*, *Aeromonas pneumophila*, *E. cloacae*, *S. aureus*, *E. coli*, and *P. aeruginosa*	• RTE foods were found to be contaminated with enteric bacteria and other potential food poisoning organisms with bacterial counts higher than the acceptable levels (<5.0 log10 CFU/ml).<br>• Coagulate negative staphylococci (23.7%), Bacillus species (21.5%), *K. pneumoniae* (18.0%), *Aeromonas pneumophila* (17.7%), E. cloacae (6.7%), *S. aureus* (3.7%), E. coli (2.2%) and *P. aeruginosa* (2.2%) were the main isolates detected.	Most RTE foods were contaminated with enteric bacteria and other potential food poisoning organisms with bacterial counts higher than the acceptable levels.

For peer review only - http://bmjopen.bmj.com/site/about/guidelines.xhtml

Jermini et. al. [33]	Raw foods (ground meat, chicken, and chicken intestine); and processed foods (dried “minnows” and “kapenta”)		*Salmonellae Spp., S. aureus, Clostridium peifringens*	• Raw foods such as ground meat, chicken, chicken intestine; and processed foods such as dried “minnows” and “kapenta” were contaminated by salmonellae or contained high populations of S. aureus in pasteurized milk.<br>• High populations (> 105) of S. aureus were detected from a sample of leftover chicken, more than 107 were detected in leftover rice, and 10 million *C. peifringens* per gram were detected in leftover beef stew sample	Time-temperature exposures during reheating had variable effects in terms of killing the microorganisms that germinated from surviving spores or that reached the foods after cooking.

For peer review only

Table 3: Key reported findings on food preparation

Study	Key findings reported
Kok et. al. [21]	• Water being used for washing utensils was left unchanged,<br>• Piles of dirty pots and dishes was left near the serving areas and RTE foods, and garbage left uncovered with many flies at the site,<br>• RTE food was left uncovered,<br>• Most of the food handlers were not wearing gloves, hairnets, or aprons
Hill et. al. [6]	• 85.5% of the vending stalls lacked soap or surface sanitizer,<br>• 71% lacked basin for washing,<br>• 75% did not have drying cloth,<br>• 76.6% of vendors handled food and money concurrently,<br>• About 57% left the food uncovered.<br>• 39% of the vendors were using their hands to pick up food items, with only 6% wearing gloves, and<br>• 29% of vendors had a wet clean sponge/cloth obtainable at the site
Mazizi et. al. [23]	• Major sources of food contamination identified were poor hygiene practices of the food vendor, holding area, and the utensils
Tshipamba et. al. [25]	• Approximately 90% of RTE meat vendors at the taxi rank exposed their meats to dust and flies,<br>• 94% of them handled money whilst serving food, and<br>• Stagnant water found in about 22% of the vending locations at the taxi-rank
McArthur-Floyd et. al. [26]	• 64% of food vendors washed their hands from elbow to finger and the remainder (36%) washed from their wrist to finger (the WHO recommends handwashing from elbow to fingers), and<br>• 62% of the vendors test their meal in the palm whilst 38% of them test it with a spoon (the best way to test a meal)
Aluko et. al. [30]	• Approximately 17% of food vendors washed their hands always after using the toilet,<br>• 63% of them rarely kept their fingernails short, and<br>• Nearly 4% of them always kept their leftover cooked food in a refrigerator, despites having unstable power supply
Odundo et. al. [31]	• Food vendors had poor hygiene practices however, men were observed to have better hygienic practices than women (P<0.05),<br>• Hygiene practice of the vendors was found to be significantly associated with training (those trained observe hygiene), and<br>• Wearing of jewellery, long and unclean nails, and lack of protective clothing were observed.
Letuka et. al. [32]	• Observed that the food handlers operated under unhygienic environment

For peer review only

Figure

Figure 1: PRISMA 2009 Flow Diagram

Figure 2: Trend of published studies relating to food safety at transport station in Africa

Supplementary File

Supplementary file 1: PRISMA-P Checklist

For peer review only - http://bmjopen.bmj.com/site/about/guidelines.xhtml

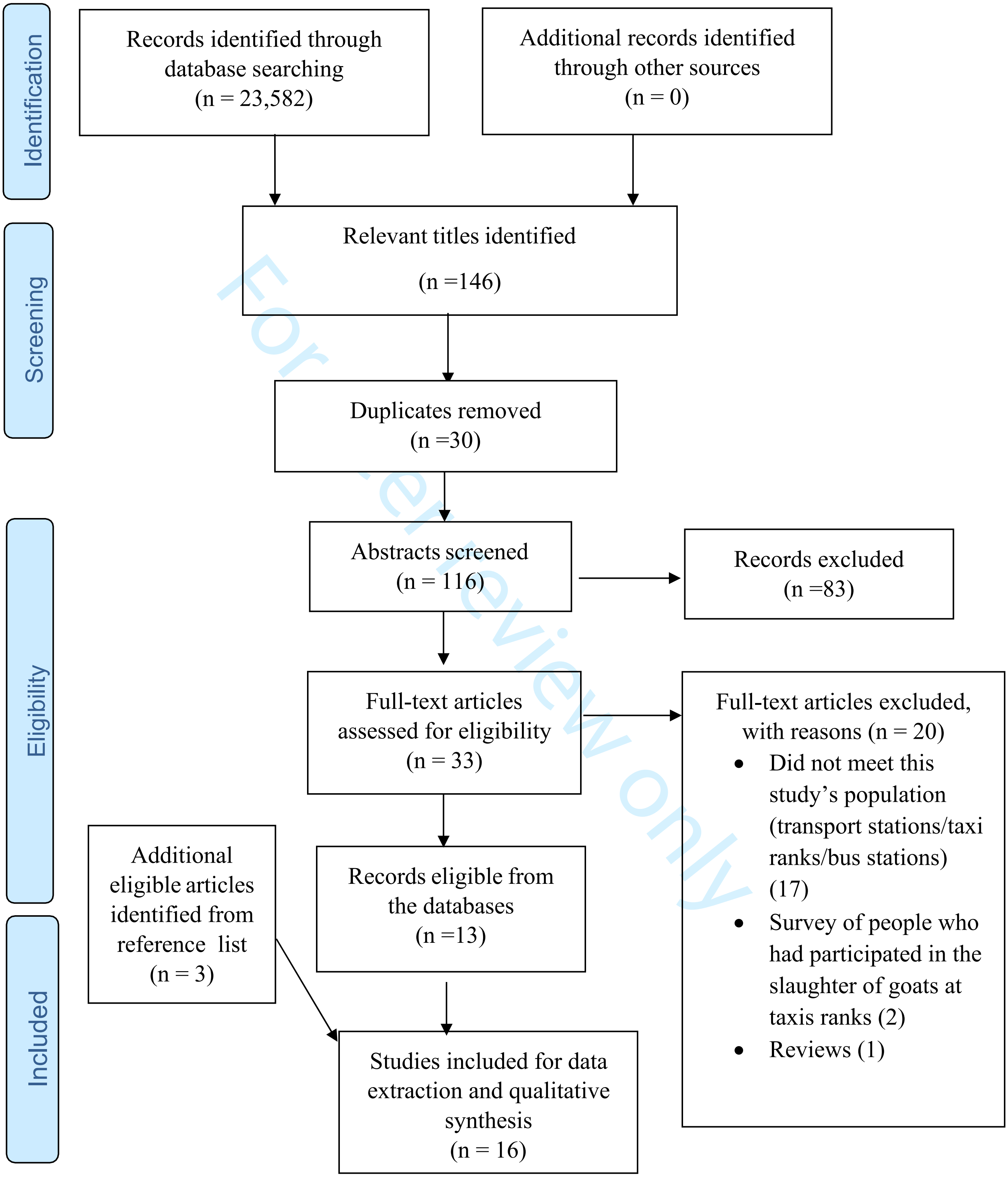

Figure 1: PRISMA 2009 Flow Diagram [18]

For peer review only

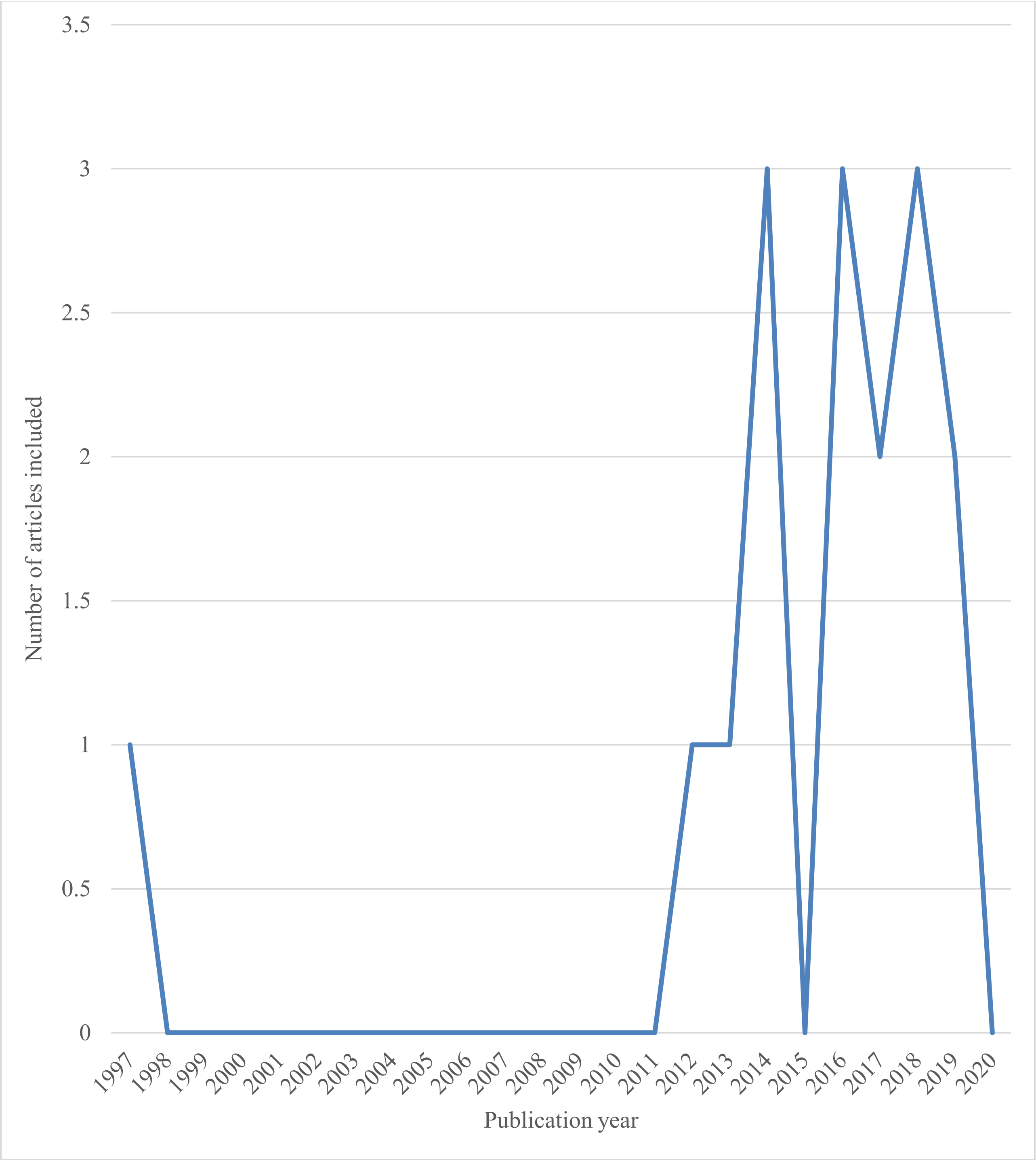

Figure 2: Trend of published studies relating to food safety at transport stations in Africa

Supplementary file 1: Electronic databases search

Search date	Database	Keywords	Search results	Number eligible
09/11/2019	EBSCOhost (Academic search complete, CINAHL with Full-text, and Health Source	SU food safety AND SU (food preparation or meal preparation or cooking) OR SU food handling OR SU food storage OR hygiene practices AND (food trading or food selling or food vending or street food) AND (transport station or taxi rank or bus station or transport exchange sites or car park or lorry park) AND africa	2,549	14
10/11/2019	PubMed	"food safety"[MeSH Terms] OR ("food"[All Fields] AND "safety"[All Fields]) OR "food safety"[All Fields]) OR ("food supply"[MeSH Terms] OR ("food"[All Fields] AND "supply"[All Fields]) OR "food supply"[All Fields] OR ("food"[All Fields] AND "security"[All Fields]) OR "food security"[All Fields]) AND (("food"[MeSH Terms] OR "food"[All Fields]) AND vending[All Fields]) OR (("food"[MeSH Terms] OR "food"[All Fields]) AND trading[All Fields]) AND streets[All Fields] OR (("motor vehicles"[MeSH Terms] OR ("motor"[All Fields] AND "vehicles"[All Fields]) OR "motor vehicles"[All Fields] OR "lorry"[All Fields]) AND parks[All Fields]) OR (("motor vehicles"[MeSH Terms] OR ("motor"[All Fields] AND "vehicles"[All Fields]) OR "motor vehicles"[All Fields] OR "lorry"[All Fields]) AND station[All Fields]) OR (taxi[All Fields] AND ranks[All Fields]) AND ("algeria"[MeSH Terms] OR "algeria"[All Fields]) OR ("angola"[MeSH Terms] OR "angola"[All Fields]) OR ("benin"[MeSH Terms] OR "benin"[All Fields]) OR ("botswana"[MeSH Terms] OR "botswana"[All Fields]) OR ("burkina faso"[MeSH Terms] OR ("burkina"[All Fields] AND "faso"[All Fields]) OR "burkina faso"[All Fields]) OR ("burundi"[MeSH Terms] OR "burundi"[All Fields]) OR ("cameroon"[MeSH Terms] OR "cameroon"[All Fields]) OR ("cabo verde"[MeSH Terms] OR ("cabo"[All Fields] AND "verde"[All Fields]) OR "cabo verde"[All Fields] OR ("cape"[All Fields] AND "verde"[All Fields]) OR "cape verde"[All Fields]) OR ("central african republic"[MeSH Terms]	2,834	33

For peer review only

		OR ("central"[All Fields] AND "african"[All Fields] AND "republic"[All Fields]) OR "central african republic"[All Fields]) OR ("chad"[MeSH Terms] OR "chad"[All Fields]) OR ("democratic republic of the congo"[MeSH Terms] OR ("democratic"[All Fields] AND "republic"[All Fields] AND "congo"[All Fields]) OR "democratic republic of the congo"[All Fields]) OR ("congo"[MeSH Terms] OR "congo"[All Fields] OR ("republic"[All Fields] AND "congo"[All Fields]) OR "republic of the congo"[All Fields]) OR ("djibouti"[MeSH Terms] OR "djibouti"[All Fields]) OR ("egypt"[MeSH Terms] OR "egypt"[All Fields]) OR ("equatorial guinea"[MeSH Terms] OR ("equatorial"[All Fields] AND "guinea"[All Fields]) OR "equatorial guinea"[All Fields]) OR ("eritrea"[MeSH Terms] OR "eritrea"[All Fields]) OR ("ethiopia"[MeSH Terms] OR "ethiopia"[All Fields]) OR ("gabon"[MeSH Terms] OR "gabon"[All Fields]) OR ("gambia"[MeSH Terms] OR "gambia"[All Fields]) OR ("ghana"[MeSH Terms] OR "ghana"[All Fields]) OR ("guinea"[MeSH Terms] OR "guinea"[All Fields]) OR ("guinea-bissau"[MeSH Terms] OR "guinea-bissau"[All Fields] OR ("guinea"[All Fields] AND "bissau"[All Fields]) OR "guinea bissau"[All Fields]) OR ("cote d'ivoire"[MeSH Terms] OR ("cote"[All Fields] AND "d'ivoire"[All Fields]) OR "cote d'ivoire"[All Fields] OR ("ivory"[All Fields] AND "coast"[All Fields]) OR "ivory coast"[All Fields]) OR ("kenya"[MeSH Terms] OR "kenya"[All Fields]) OR ("lesotho"[MeSH Terms] OR "lesotho"[All Fields]) OR ("liberia"[MeSH Terms] OR "liberia"[All Fields]) OR ("libya"[MeSH Terms] OR "libya"[All Fields]) OR ("madagascar"[MeSH Terms] OR "madagascar"[All Fields]) OR ("malawi"[MeSH Terms] OR "malawi"[All Fields]) OR ("mali"[MeSH Terms] OR "mali"[All Fields]) OR ("mauritania"[MeSH Terms] OR "mauritania"[All Fields]) OR ("mauritius"[MeSH Terms] OR "mauritius"[All Fields]) OR ("morocco"[MeSH Terms] OR "morocco"[All Fields]) OR ("mozambique"[MeSH Terms] OR "mozambique"[All Fields]) OR ("namibia"[MeSH Terms] OR "namibia"[All Fields]) OR ("niger"[MeSH Terms] OR "niger"[All Fields]) OR ("nigeria"[MeSH Terms] OR "nigeria"[All Fields]) OR ("rwanda"[MeSH Terms] OR		

For peer review only

		"rwanda"[All Fields]) OR ("sao tome and principe"[MeSH Terms] OR ("sao"[All Fields] AND "tome"[All Fields] AND "principe"[All Fields]) OR "sao tome and principe"[All Fields]) OR ("senegal"[MeSH Terms] OR "senegal"[All Fields]) OR ("seychelles"[MeSH Terms] OR "seychelles"[All Fields]) OR ("sierra leone"[MeSH Terms] OR ("sierra"[All Fields] AND "leone"[All Fields]) OR "sierra leone"[All Fields]) OR ("somalia"[MeSH Terms] OR "somalia"[All Fields]) OR (("south africa"[MeSH Terms] OR ("south"[All Fields] AND "africa"[All Fields]) OR "south africa"[All Fields]) AND South[All Fields]) OR ("sudan"[MeSH Terms] OR "sudan"[All Fields]) OR ("sudan"[MeSH Terms] OR "sudan"[All Fields]) OR ("eswatini"[MeSH Terms] OR "eswatini"[All Fields] OR "swaziland"[All Fields]) OR ("tanzania"[MeSH Terms] OR "tanzania"[All Fields]) OR ("togo"[MeSH Terms] OR "togo"[All Fields]) OR ("tunisia"[MeSH Terms] OR "tunisia"[All Fields]) OR ("uganda"[MeSH Terms] OR "uganda"[All Fields]) OR ("zambia"[MeSH Terms] OR "zambia"[All Fields]) OR ("zimbabwe"[MeSH Terms] OR "zimbabwe"[All Fields]) Filters: Free full text, Comparative Study, Observational Study, Randomized Controlled Trial, Humans, English, MEDLINE		
11/11/2019	Web of Science	(food safety AND food preparation OR meal preparation OR cooking OR food handling OR food storage OR hygiene practices AND food trading OR food selling OR food vending or street food AND transport station OR taxi rank OR bus station OR transport exchange sites OR car park or lorry park AND africa)Refined by: Open Access: (OPEN ACCESS) AND DOCUMENT TYPES: (ARTICLE) AND DOCUMENT TYPES: (ARTICLE) AND WEB OF SCIENCE CATEGORIES: (FOOD SCIENCE TECHNOLOGY OR PUBLIC ENVIRONMENTAL OCCUPATIONAL HEALTH) AND WEB OF SCIENCE CATEGORIES: (FOOD SCIENCE TECHNOLOGY OR PUBLIC ENVIRONMENTAL OCCUPATIONAL HEALTH OR NUTRITION DIETETICS)Timespan: All years. Indexes: SCI-EXPANDED, SSCI, A&HCI, CPCI-S, CPCI-SSH, BKCI-S, BKCI-SSH, ESCI.	8,263	36
12/11/2019	SCOPUS	food safety AND food preparation OR meal preparation OR cooking OR food handling OR food storage OR hygiene practices AND food trading OR	116	7

For peer review only - http://bmjopen.bmj.com/site/about/guidelines.xhtml

		food selling OR food vending or street food AND transport station OR taxi rank OR bus station OR transport exchange sites OR car park or lorry park AND africa		
13/11/2019	Google Scholar	food safety AND food preparation OR meal preparation OR cooking OR food handling OR food storage OR hygiene practices AND food trading OR food selling OR food vending or street food AND transport station OR taxi rank OR bus station OR transport exchange sites OR car park or lorry park AND africa	9,820	56
			23,582	146
**Duplicates**				**30**

For peer review only

Supplementary file 1: Preferred Reporting Items for Systematic reviews and Meta-Analyses extension for Scoping Reviews (PRISMA-ScR) Checklist

SECTION	ITEM	PRISMA-ScR CHECKLIST ITEM	REPORTED ON PAGE #
**TITLE**			
Title	1	Identify the report as a scoping review.	
**ABSTRACT**			
Structured summary	2	Provide a structured summary that includes (as applicable): background, objectives, eligibility criteria, sources of evidence, charting methods, results, and conclusions that relate to the review questions and objectives.	
**INTRODUCTION**			
Rationale	3	Describe the rationale for the review in the context of what is already known. Explain why the review questions/objectives lend themselves to a scoping review approach.	
Objectives	4	Provide an explicit statement of the questions and objectives being addressed with reference to their key elements (e.g., population or participants, concepts, and context) or other relevant key elements used to conceptualize the review questions and/or objectives.	
**METHODS**			
Protocol and registration	5	Indicate whether a review protocol exists; state if and where it can be accessed (e.g., a Web address); and if available, provide registration information, including the registration number.	
Eligibility criteria	6	Specify characteristics of the sources of evidence used as eligibility criteria (e.g., years considered, language, and publication status), and provide a rationale.	
Information sources*	7	Describe all information sources in the search (e.g., databases with dates of coverage and contact with authors to identify additional sources), as well as the date the most recent search was executed.	
Search	8	Present the full electronic search strategy for at least 1 database, including any limits used, such that it could be repeated.	
Selection of sources of evidence†	9	State the process for selecting sources of evidence (i.e., screening and eligibility) included in the scoping review.	
Data charting process‡	10	Describe the methods of charting data from the included sources of evidence (e.g., calibrated forms or forms that have been tested by the team before their use, and whether data charting was done independently or in duplicate) and any processes for obtaining and confirming data from investigators.	
Data items	11	List and define all variables for which data were sought and any assumptions and simplifications made.	
Critical appraisal of individual sources of evidence§	12	If done, provide a rationale for conducting a critical appraisal of included sources of evidence; describe the methods used and how this information was used in any data synthesis (if appropriate).	
Synthesis of results	13	Describe the methods of handling and summarizing the data that were charted.	

St. Michael's
Inspired Care. Inspiring Science.

For peer review only - http://bmjopen.bmj.com/site/about/guidelines.xhtml

SECTION	ITEM	PRISMA-ScR CHECKLIST ITEM	REPORTED ON PAGE #
**RESULTS**			
Selection of sources of evidence	14	Give numbers of sources of evidence screened, assessed for eligibility, and included in the review, with reasons for exclusions at each stage, ideally using a flow diagram.	
Characteristics of sources of evidence	15	For each source of evidence, present characteristics for which data were charted and provide the citations.	
Critical appraisal within sources of evidence	16	If done, present data on critical appraisal of included sources of evidence (see item 12).	
Results of individual sources of evidence	17	For each included source of evidence, present the relevant data that were charted that relate to the review questions and objectives.	
Synthesis of results	18	Summarize and/or present the charting results as they relate to the review questions and objectives.	
**DISCUSSION**			
Summary of evidence	19	Summarize the main results (including an overview of concepts, themes, and types of evidence available), link to the review questions and objectives, and consider the relevance to key groups.	
Limitations	20	Discuss the limitations of the scoping review process.	
Conclusions	21	Provide a general interpretation of the results with respect to the review questions and objectives, as well as potential implications and/or next steps.	
**FUNDING**			
Funding	22	Describe sources of funding for the included sources of evidence, as well as sources of funding for the scoping review. Describe the role of the funders of the scoping review.	

JBI = Joanna Briggs Institute; PRISMA-ScR = Preferred Reporting Items for Systematic reviews and Meta-Analyses extension for Scoping Reviews.
* Where *sources of evidence* (see second footnote) are compiled from, such as bibliographic databases, social media platforms, and Web sites.
† A more inclusive/heterogeneous term used to account for the different types of evidence or data sources (e.g., quantitative and/or qualitative research, expert opinion, and policy documents) that may be eligible in a scoping review as opposed to only studies. This is not to be confused with *information sources* (see first footnote).
‡ The frameworks by Arksey and O'Malley (6) and Levac and colleagues (7) and the JBI guidance (4, 5) refer to the process of data extraction in a scoping review as data charting.
§ The process of systematically examining research evidence to assess its validity, results, and relevance before using it to inform a decision. This term is used for items 12 and 19 instead of "risk of bias" (which is more applicable to systematic reviews of interventions) to include and acknowledge the various sources of evidence that may be used in a scoping review (e.g., quantitative and/or qualitative research, expert opinion, and policy document).

From: Tricco AC, Lillie E, Zarin W, O'Brien KK, Colquhoun H, Levac D, et al. PRISMA Extension for Scoping Reviews (PRISMA-ScR): Checklist and Explanation. Ann Intern Med. ;169:467–473. doi: 10.7326/M18-0850

For peer review only

A Scoping Review of Food Safety at Transport stations in Africa

Journal:	*BMJ Open*
Manuscript ID	bmjopen-2021-053856.R2
Article Type:	Original research
Date Submitted by the Author:	10-Oct-2021
Complete List of Authors:	Ncama, Busisiwe; University of KwaZulu-Natal College of Health Sciences<br>Kuupiel , Desmond ; University of KwaZulu-Natal College of Health Sciences, Public Health Medicine; Research for Sustainable Development Consult,<br>Duma, Sinegugu; University of KwaZulu-Natal College of Health Sciences<br>Mchunu , Gugu ; University of KwaZulu-Natal College of Health Sciences<br>Guga , Phindile ; University of KwaZulu-Natal College of Health Sciences<br>Slotow, Rob; University of KwaZulu-Natal
**Primary Subject Heading**:	Public health
Secondary Subject Heading:	Public health
Keywords:	Public health < INFECTIOUS DISEASES, PUBLIC HEALTH, Health & safety < HEALTH SERVICES ADMINISTRATION & MANAGEMENT

SCHOLARONE™
Manuscripts

I, the Submitting Author has the right to grant and does grant on behalf of all authors of the Work (as defined in the below author licence), an exclusive licence and/or a non-exclusive licence for contributions from authors who are: i) UK Crown employees; ii) where BMJ has agreed a CC-BY licence shall apply, and/or iii) in accordance with the terms applicable for US Federal Government officers or employees acting as part of their official duties; on a worldwide, perpetual, irrevocable, royalty-free basis to BMJ Publishing Group Ltd ("BMJ") its licensees and where the relevant Journal is co-owned by BMJ to the co-owners of the Journal, to publish the Work in this journal and any other BMJ products and to exploit all rights, as set out in our licence.

The Submitting Author accepts and understands that any supply made under these terms is made by BMJ to the Submitting Author unless you are acting as an employee on behalf of your employer or a postgraduate student of an affiliated institution which is paying any applicable article publishing charge ("APC") for Open Access articles. Where the Submitting Author wishes to make the Work available on an Open Access basis (and intends to pay the relevant APC), the terms of reuse of such Open Access shall be governed by a Creative Commons licence – details of these licences and which Creative Commons licence will apply to this Work are set out in our licence referred to above.

Other than as permitted in any relevant BMJ Author's Self Archiving Policies, I confirm this Work has not been accepted for publication elsewhere, is not being considered for publication elsewhere and does not duplicate material already published. I confirm all authors consent to publication of this Work and authorise the granting of this licence.

For peer review only

A Scoping Review of Food Safety at Transport stations in Africa

Busisiwe P. Ncama[1], Desmond Kuupiel[1,2], Sinegugu E. Duma[1], Gugu Mchunu[1], Phindile Guga[1], Rob Slotow[3]

[1]School of Nursing and Public Health, College of Health Sciences, University of KwaZulu-Natal, Durban, South Africa

[2]Research for Sustainable Development Consult, Sunyani, Ghana

[3]School of Life Sciences, College of Agriculture, Engineering and Science, University of Kwazulu-Natal, Pietermaritzburg, South Africa.

Corresponding Author: Desmond Kuupiel (PhD)

Address: 2nd Floor George Campbell Building, Department of Public Health Medicine, School of Nursing and Public Health, University of KwaZulu-Natal, Durban, 4001, South Africa

Email address: desmondkuupiel98@hotmail.com / KuupielD@ukzn.ac.za

Tel: +27735568200/ +233550972968

Email addresses of co-authors

Prof. Busisiwe P. Ncama (Ncamab@ukzn.ac.za)

Prof. Sinegugu E. Duma (DumaS1@ukzn.ac.za)

Prof. Gugu Mchunu (Mchunug@ukzn.ac.za)

Ms. Phindile Guga (phindile.guga@gmail.com)

Prof. Rob Slotow (Slotow@ukzn.ac.za)

For peer review only - http://bmjopen.bmj.com/site/about/guidelines.xhtml

Abstract

Objective: The World Health Organization (WHO) has declared food safety as a public health concern. Transport hubs such as taxi ranks, bus stations, and other transport exchange sites are major food trading/purchasing sites, particularly in Africa. Research evidence is needed to improve food safety policies and ensure consumption of safe food, owing to the increasing burden of foodborne diseases, particularly in the WHO Africa Region. We systematically mapped and described research evidence on food safety at transport stations in Africa.

Design: A scoping review guided by the Arksey and O'Malley framework.

Data sources: We searched for original research articles in PubMed, Web of Science, and EBSCOhost (Academic search complete, CINAHL with Full-text, and Health Source), SCOPUS, and Google Scholar from their inception to 25th October 2020.

Eligibility criteria for selecting studies: We included studies that focused on food safety, involved transport stations, involved African countries, and were published in English.

Data extraction and synthesis: Data extraction was performed by two reviewers using a piloted-tested form. Thematic analysis was used to organise the data into themes and sub-themes, and a narrative summary of the findings is presented.

Results: Of the total 23,852 articles obtained from the database searches, 16 studies published in six countries met the inclusion criteria. These 16 studies were published between 1997 and 2019, with the most (5) in 2014. Of the 16 studies, 43.8% (7) were conducted in South Africa, three studies in Ghana, two in Ethiopia, and one study each in Nigeria, Kenya, Lesotho, and Zambia. Most (44.4%) of the included studies focused on microbial safety of food; few studies (22.2%) focused on hygienic practices, and one study investigated the perspective of consumers or buyers. Microbes detected in the foods samples were *Salmonella* spp, *E. coli*, *Shigella* spp, *Bacillus* sp, *Staphylococcus aureus*, which resulted mainly from poor hygiene practices.

Conclusions: There is limited research that focused on food safety at transport stations in Africa, especially on aspects such as hygiene practices, food storage, and occupational health and food safety. Therefore, we recommend more research in these areas, using various primary study designs, to inform and improve food safety policies and practices for transport stations in African countries alongside improving access to clean water/handwashing facilities, and undertaking structural changes to facilitate behaviours and monitoring for unintended consequences such as livelihoods of vulnerable populations.

For peer review only - http://bmjopen.bmj.com/site/about/guidelines.xhtml

Article summary

Strengths and limitations of this study

- To the best of our knowledge, this is the first scoping review to systematically explore literature and describe research evidence on food safety at transport stations as well as identify gaps for future research in Africa.
- This scoping review's evidence sources were searched using a systematic approach, and duplicate screening.
- This review is limited to Africa as well as English language publications.

For peer review only

For peer review only - http://bmjopen.bmj.com/site/about/guidelines.xhtml

Background

The World Health Organisation (WHO) estimates that more than 600 million people fall sick (almost 1 in 10 people) with foodborne diseases annually, of which nearly 420 000 people die, and about 33 million years of healthy lives are lost every year worldwide [1, 2]. The burden of foodborne diseases is estimated to be highest in the WHO African and South-East Asia Regions, mainly occurring among vulnerable populations such as infants, young children, pregnant women, older people, poor people, and individuals with underlying illnesses [3]. Food contamination mostly results throughout the food supply chain (from the procedures used in processing the foods, inadequate storage temperatures, unhygienic practices by food handlers, poor sanitation at cooking places/vending areas, poor waste management, and inadequate treatment of leftovers) [4].

Unsafe food has negative implications on health systems, and affects the development and national economies of countries, as well as trade [3]. Therefore, eating unsafe foods poses a significant public health threat. To avert the consequences of unsafe food on health systems, and to sustain national economies, development, trade, and tourism [5, 6], the WHO in 2006 declared food safety as a global public health concern [7, 8]. "Food safety refers to routines in the preparation, handling and storage of food meant to prevent foodborne illness and injury" [5]. To reduce the incidence of food-related diseases, particularly in high burden regions, the observations of food safety measures/precautions at all levels of the food processing chain, including the places where food is prepared and sold, are critical [9, 10].

Like other WHO Regions, especially in low-and-middle-income countries, food trading in the Africa Region takes place at several formal and informal places, such as in the markets, restaurants, streets, open spaces in academic institutions, and transport stations (taxi ranks, bus stations, lorry parks), and other transport exchange sites. Food vending at public spaces serves as a source of livelihood [6, 10, 11], and more than two billion people eat food sold at various vending locations. including transportations stations on daily basis globally [12, 13]. To this end, evidence is essential to inform in-country policies/guidelines, and further research, to ensure that food prepared and sold at transport stations promotes livelihoods, nutrition, food safety, and environmentally sustainable practices. This scoping review systematically mapped literature focused on food safety at transport stations in Africa, to summarise evidence and identify gaps.

For peer review only - http://bmjopen.bmj.com/site/about/guidelines.xhtml

Methods

Scope of the review

The Arksey & O'Malley framework (research question identification; identifying relevant studies; selection of study; data charting, collating, and summarising and reporting the findings [14, 15]) was employed to scope and synthesise literature to answer the question - what evidence exists on food safety at transport stations in Africa? This review's study protocol was developed *a priori* [16]. This study included published peer-reviewed articles that reported findings from any African country/countries, focused on food safety, and involved transport stations. However, this study was limited to English publications (due to lack of expertise in other international languages), and primary study designs [16]. A detailed description of this scoping review study eligibility criteria is captured in the published protocol [16]. We followed the Preferred Reporting Items for Systematic and Meta-analysis (PRISMA) extension for Scoping Reviews checklist to report this study [17].

Identify relevant studies

We searched for primary research articles relating to food safety at transport stations in PubMed, Web of Science, and EBSCOhost (Academic search complete, CINAHL with Full-text, and Health Source), SCOPUS, and Google Scholar from their inception to 25th October 2020. To enable the capturing of all relevant articles, a comprehensive search strategy (developed in consultation with an expert librarian) consisting of keywords, Boolean terms (AND/OR), and Medical Subject Heading terms, was used for the electronic database search (Supplementary file 1). Syntax was modified appropriately where needed. Filters such as date and study design were not applied during the literature search in the databases. DK and PG independently conducted the database search and title screening, and imported all potentially eligible articles onto an EndNote Library. The reference lists of all included articles were also screened for potentially relevant articles using the same approach.

Selection of articles

Prior to the abstract screening, the 'find duplicates' function in EndNote was used to find all duplicate articles, and they were removed from the library. A screening form was developed in Google forms, using this study's eligibility criteria, for the abstract and full text screening phases. Two reviewers (co-authors) independently screened the abstracts as well as the full text articles. Discrepancies that arose during the abstract stage were resolved by discussion among

For peer review only - http://bmjopen.bmj.com/site/about/guidelines.xhtml

the review team until a consensus was reached. At the full text screening phase, discrepancies were resolved by a third reviewer. All the additional articles identified from the reference list of the included articles equally underwent full text assessment. The PRISMA flow diagram was employed to account for all the articles involved [18].

Charting the data

A data extraction form was designed consisting of the following: Author(s) and publication details, country of study, study design, study setting, study population, sample size, sex, study findings, and recommendations. To ensure consistency and reliability, two reviewers piloted the data extraction sheet using a random sample of three included studies. The pilot testing of the form also enabled the review team to discuss discrepancies, and to revise the data extraction form prior to its final usage. Subsequently, two reviewers conducted the data extraction for the remaining 15 included studies using both inductive and deductive approaches. The review team resolved all discrepancies at this stage through discussion.

Collating, summarizing, and reporting the results

This study subsequently employed thematic analysis, and collated all the emerging themes and sub-themes relating to food safety. A summary of the findings from the included studies is presented narratively.

Patient and Public Involvement

No patient involved

Results

Of the 23,852 articles obtained from the database searches (see Figure 1 flow diagram), 146 articles met the eligibility criteria at the title screening stage. Using EndNote "Find Duplicates" function, 30 duplicates were found and removed before abstract screening was conducted. Subsequently, 83 articles were removed at the abstract screening, and 20 at full text (17 of these did not include transport stations/taxi ranks/bus stations, but did involve sale from market centres, public places, chop bars, mini restaurants, major streets, and sidewalks, and were excluded). Finally, 13 studies were included, and, from a manual search of their reference lists, a further three articles were added, giving a total of 16 articles for further analysis.

For peer review only - http://bmjopen.bmj.com/site/about/guidelines.xhtml

Characteristics of the included studies

Table 1 A & B present a summary of the characteristics of the included studies. Of the 16 included studies, about fourty-four (43.8%) were conducted in South Africa [19-25], three (18.8%) in Ghana [4, 26, 27], two (12.5%) in Ethiopia [28, 29], and one (6.2%) each in Nigeria [30], Kenya [31], Lesotho [32], and Zambia [33]. Most of the studies were published in the last six years, however, no published study was found in 2015 and 2020 (Figure 2). Fifteen (93.8%) of the included studies were cross-sectional studies, and one (6.2%) was a mixed-method study. Of the 16 included studies, 50.0% reported on microbial safety of food [4, 20, 23, 27-29, 33, 34], and 25.0% reported hygiene practices of food handlers/vendors [6, 21, 30, 31]. One included study each reported on the following: occupational health and food safety risk [24]; knowledge of hygiene practice [26]; hygiene practices of food handlers/vendors and microbial safety [25]; and knowledge of food safety measures and hygiene practice by food handlers/vendors [32].

Findings from the included studies

Microbial safety of food

Of the nine included studies that reported findings on microbial safety of food, 44.4% were conducted in South Africa [19, 20, 23, 25], 22.2% each in Ghana [4, 27] and Ethiopia [28, 29], and the last 11.1% in Zambia [33]. Seven of the eight studies reported unacceptable levels of microbes in the food [4, 20, 23, 27-29, 33, 34]. Table 2 A, B & C present a summary of the key findings as well as the sample type, analytic approach, and the microbes reported.

Hygiene practices of food handlers/vendors

Food preparation

Of the 16 included studies, eight reported research findings relating to food preparation. Fifty percent of these eight studies were from South Africa [6, 21, 23, 25], and the remainder were from Ghana [26], Nigeria [30], Kenya[31], and Lesotho [32]. The studies in South Africa focused on the following: hygiene practices and implications for consumers [21]; food and nutrition knowledge as well as practices related to food preparation [6], the effect of hygiene practices and attitudes of meat vendors [25], and sources of food contamination [23]. The study from Ghana investigated how fast food operators washed their hands [26], whilst the studies from Nigeria, Kenya, and Lesotho evaluated food safety and sanitary practices [30]; food

For peer review only - http://bmjopen.bmj.com/site/about/guidelines.xhtml

vendors and hygiene practices [31]; and food safety knowledge, attitudes and practices of food vendors and consumers' perceptions [32]. A summary of the key findings from these studies is presented below (Table 3).

Knowledge of hygiene practices/food safety precautions

In Ghana, McArthur-Floyd et. al. study [26], revealed that the majority (94%) of fast food operators knew food safety precautions [26]. Letuka et. al. study [32] in Lesotho, indicated that 95% of food vendors did not know washing utensils with detergents helps reduce contamination [32]. The mean knowledge (49%±11) of the food vendors included in the study was considered poor [32]. About 6% of the consumers that participated in the study chose not to buy food sold at taxi ranks due to food safety issues and hygiene [32].

Occupational health and food safety risk

In South Africa, Qekwana et.al. [24]evaluated the occupational health and food safety risks associated with the traditional slaughter of goats, and the consumption of such meat [24]. Approximately 63% of the practitioners were not wearing protective clothing during slaughter, and about 78% of practitioners did not know their health status [24]. Almost 83% of the practitioners hung up their carcass to facilitate bleeding, flaying, and evisceration [24]. The study further observed that none of the practitioners practiced meat inspection [24]. In Nigeria, Aluko et al. [30] study revealed that approximately 62% of the vendors had no formal training, and their medical status was also unknown [30].

Discussion

This scoping review mapped evidence on food safety at transport stations in Africa, and revealed a very low number of papers that are published in this area, given many African employees in both formal and informal sectors commute through these transport hubs [12, 13]. An average of one paper per year relating to food safety at transport hubs in Africa as revealed by this review is simply not enough. Nonetheless, the few papers depict an imbalance of research, with most focused on microbial safety [4, 20, 23, 27-29, 33, 34], and few on socio-economic aspects such as hygiene practices [6, 21, 30, 31], and occupational health and food safety risk [24]. Moreover, this review revealed no study evaluated the storage of food or how the food is transported to the vending site.

For peer review only - http://bmjopen.bmj.com/site/about/guidelines.xhtml

As evidence by this review, most of the food sold at transport hubs does not meet the minimum standards and is not safe for consumption due to the presence of several microbes [4, 19, 23, 25, 27, 29, 33, 35]. There are several reasons for this such as poor practices relating to hygiene, storage, preparation, cooking, cleaning, and serving [4, 20, 23, 27-29, 33, 34]. However, these findings are similar to previous review findings involving markets [36], homes and restaurants [37]. A recent publication by Gizaw [36] indicated that several studies reported microbial contamination of foods sold in the market, with bacteria and fungi similar to those identified in our review [36]. Also, a review by the WHO reported that the main factors contributing to foodborne disease outbreaks in homes or restaurants were poor temperature control in preparing, cooking, and storing food [37]. Although very few papers were found by this review, the evidence is compelling that there should be policy interventions to address issues relating poor hygiene practices, including food storage, preparation, cooking, cleaning, and serving by food handlers at transport hubs, not only in South Africa but across Africa.

Similar to a previous scoping review [38], most of the included papers were published within the last six years but, no published study was found in 2015 and 2020. Whilst the reason for the lack of published papers in 2015 might be difficult to determine, the COVID-19 pandemic which resulted in "covidisation" of research might be the reason for the lack of publication in this field of research in 2020. Although we cannot conclude that no primary research has been conducted in these countries focusing on the safety of food sold at transport stations, it suggests a research/publication gap. Food safety research is, perhaps, more relevant now than ever in Africa, since the burden of foodborne diseases is rising annually, resulting in the declaration of food safety as a public health concern by the WHO [7, 8]. Aside from this, most commuters tend to buy ready-to-eat (RTE) food from street food vendors, including those at transport hubs [39, 40]; hence, the sale of food at transport stations is rising [40, 41], particularly in Africa [6] partly due to an increase in demand for RTE, and the employment opportunities it offers to many individuals who otherwise would not have had any source of income [38, 42]. Even more worrying is the fact that most of the articles included that focused on microbial safety, reported high levels of food contamination with several microorganisms, especially *Salmonella* spp and *E. coli* [4, 19, 23, 25, 27, 29, 33, 35]. Therefore, more research is needed across African countries to prevent potential negative consequences.

Our study findings have implications for practice and research. For instance, the likelihood of food poisoning with microbes such as *Salmonella* spp, *E. coli.*, *Shigella* spp, *Bacillus* spp, *S. aureus,* and several others, revealed by most of the included studies that focused on microbial

For peer review only - http://bmjopen.bmj.com/site/about/guidelines.xhtml

contamination of food, is alarming. This, if not checked, could further worsen the already high burden of foodborne diseases in a continent that has several of its countries already experiencing many health systems and economic challenges. Aside from this, the majority of individuals who commute through transport hubs, possible will purchase a meal from a transport hub/exchanges site, which may be the only meal of the day [12, 13], and yet the food safety standards are poor [4, 20, 23, 27-29, 33, 34]. Thus, if not checked, the excess cases of foodborne diseases from any outbreak will further impact negatively on the already challenged public health systems in Africa. Also, poor people who are exposed to these unsafe foods get an infection, may have to pay more for healthcare, which can further exacerbate their poverty situation. Moreover, people who are already living in extreme poverty who get exposed to foodborne disease may not even make it to the hospital for care and can end up dying at home [43].

Good hygiene and sanitation practices, such as adequate hand washing, adequate washing and storage of pots and dishes, good waste management, observation of food preparation standards and serving etiquette, among others, have the potential to reduce the risk of food contamination from both biological and non-biological hazards, yet this study reveals fewer studies that focused on hygienic practices. We, therefore, recommend more research to further inform contextualised policy decisions aimed at improving hygiene and sanitation practices by food vendors at transport stations. Also, very relevant to ensuring food safety is the occupational health practices of the vendors. Regular food handling tests and food inspections, conducted by the appropriate local authorities, should be mandatory in all African countries. Food handler tests should seek to ensure that food vendors are fit healthwise to prepare and serve food meant for public consumption. However, our review found limited studies that evaluated occupational health and food safety. Considering that evidence from South Africa and Nigeria suggests about 78% and 62% of food vendors do not know their health status [30, 44], and the increasing number of informal food sellers at various transport exchange sites, future studies are recommended to focus on occupational health and food safety in Africa. The means and manner of storing food, especially leftover RTE food, can either increase or reduce the risk of food contamination, but, again, this scoping review found no study that focused on food storage practices of the vendors at transport stations. Also essential, and yet we did not find any study focusing on it, is the quality of food (nutritious aspects) of the meals sold at transport stations. Eating a well nourishing diet or balanced meals is critical to ensure good health [45-47], hence, we encourage future primary studies to include the nutritious aspects. Such studies may help

For peer review only - http://bmjopen.bmj.com/site/about/guidelines.xhtml

streamline guidelines or inform policies to improve the quality of the food sold at transport exchange sites or taxi ranks. Moreover, this review found that the majority (17 out of 18) of the respondents in the included studies were the vendors (mostly females) or food samples taken from the vendors. The perspectives of consumers (buyers) or commuters regarding food safety at transport stations are also very relevant, and we recommend future research to involve them. A comparative study to investigate food safety practices among males and females food vendors at transport stations might be relevant since many males are now getting involved in the business [6, 48, 49].

To the best of our knowledge, this study is the first scoping review that systematically mapped literature relating to food safety at transport stations in Africa. A major strength of our study method is that it permits the inclusion of multiple study designs. Also, the choice of this study method permitted us to highlight literature gaps, and made recommendations for future research. Aside from this, we conducted a thorough search in six databases using a comprehensive search strategy which enabled us to capture the most relevant articles to answer the review question. Moreover, two independent reviewers were used to select the studies and perform data extraction processes which helped to prevent selection bias and ensured the reliability and trustworthiness of this study results. Despite this, our scoping review has many limitations. This study included only original study peer reviewed papers, which resulted in the exclusion of one review paper [38], and one Masters' dissertation [50]. We did not also consult the websites of WHO and the Food and Agriculture Organisation websites for possible relevant studies. Furthermore, this study cannot be generalised since the search was limited to African countries only. Although date limitation was removed, we limited the publication language to English only, which perhaps eliminated relevant articles published in other languages. Despite these limitations, this study has provided essential evidence relating to food safety at transport stations and has shown literature gaps to guide future research.

Conclusion

Based on this scoping review's eligibility criteria, our study results suggest there is limited research focusing on food safety at transport stations in Africa. Most of the existing published studies are focused on microbial safety of food, and very few/none on other aspects such as hygiene practices, food storage, occupational health and food safety, and nutrition. Hence, we recommend more primary research involving community members and policy makers in these areas going forward alongside improving access to clean water/handwashing facilities, and

For peer review only - http://bmjopen.bmj.com/site/about/guidelines.xhtml

undertaking structural changes to facilitate behaviours and monitoring for unintended consequences such as livelihoods of vulnerable populations.

Abbreviations

PRISMA- Preferred Reporting Items for Systematic and Meta-analysis

RTE- Ready-to-eat

WHO- World Health Organization

Ethics and dissemination

Not required. This study did not include human participants.

Data availability statement

Not applicable. All sources of data have been adequately referenced

Patient and Public Involvement

No patient involved

Statements

Acknowledgments

Support for this work was provided by the Sustainable and Healthy Food Systems (SHEFS) Programme, supported through the Wellcome Trust's Our Planet, Our Health Programme

Authors' contributions

BPN, DK, SED, SM, and RS conceptualized and designed the study. DK developed and designed the database search strategy and conducted the search. PG contributed to the screening of the studies and data extraction. DK wrote the draft manuscript and BPN, SED, GM, and RS critically review it and made revisions. All the authors approved the final version of the manuscript.

Funding

Funding for this work was provided by the Sustainable and Healthy Food Systems (SHEFS) Programme, supported through the Wellcome Trust's Our Planet, Our Health Programme [grant number: 205200/Z/16/Z]. The funder played no role in the literature search and writing of the manuscript.

For peer review only - http://bmjopen.bmj.com/site/about/guidelines.xhtml

Competing interests

None declared

References

1. The Lancet Gastroenterology H. Food safety really is everyone's business. The lancet Gastroenterology & hepatology. 2019;4(8):571.

2. World Health Organization. Estimating the burden of foodborne diseases Geneva: World Health Organization; 2015 [cited 2019 25/10/2019]. Available from: https://www.who.int/activities/estimating-the-burden-of-foodborne-diseases.

3. World Health Organization. Food Safety Geneva: World Health Organization; 2016 [cited 2020 17/11/2020]. Available from: https://www.afro.who.int/health-topics/food-safety.

4. Feglo P, Sakyi K. Bacterial contamination of street vending food in Kumasi, Ghana. Journal of Medical Biomedical Sciences. 2012;1(1):1-8.

5. Organization WH. Food Safety: What you should know Geneva: World Health Organization; 2015 [cited 2019 25/10/2019]. Available from: http://www.searo.who.int/entity/world_health_day/2015/whd-what-you-should-know/en/.

6. Hill J, Mchiza Z, Puoane T, Steyn NP. The development of an evidence-based street food vending model within a socioecological framework: a guide for African countries. PloS one. 2019;14(10):e0223535.

7. World Health Organization. Five keys to safer food manual Geneva: World Health Organization; 2006 [cited 2019 25/10/2019]. Available from: https://www.who.int/foodsafety/publications/consumer/manual_keys.pdf.

8. Lamin-Boima P. Knowledge, Attitude And Practice Of Street Food Vendors In Selected Schools Within Bo City Southern Sierra Leone.

9. Lues JFR, Rasephei MR, Venter P, Theron MM. Assessing food safety and associated food handling practices in street food vending. International Journal of Environmental Health Research. 2006;16(5):319-28.

10. Abrahale K, Sousa S, Albuquerque G, Padrao P, Lunet N. Street food research worldwide: a scoping review. Journal of human nutrition and dietetics : the official journal of the British Dietetic Association. 2019;32(2):152-74.

For peer review only - http://bmjopen.bmj.com/site/about/guidelines.xhtml

11. Liu Z, Zhang G, Zhang X. Urban street foods in Shijiazhuang city, China: Current status, safety practices and risk mitigating strategies. Food Control. 2014;41:212-8.

12. Food and Agriculture Organization. Selling street and snack foods Rome: Food and Agriculture Organization; 2011 [cited 2019 25/10/2019]. Available from: http://www.fao.org/3/a-i2474e.pdf.

13. Gelormini M, Damasceno A, Lopes SA, Malo S, Chongole C, Muholove P, et al. Street Food Environment in Maputo (STOOD Map): a Cross-Sectional Study in Mozambique. JMIR research protocols. 2015;4(3):e98.

14. Arksey H, O'Malley L. Scoping studies: towards a methodological framework. International Journal of Social Research Methodology. 2005;8(1):19-32.

15. Levac D, Colquhoun H, O'Brien KK. Scoping studies: advancing the methodology. Implementation Science. 2010;5(1):69.

16. Ncama BP, Kuupiel D, Duma SE, McHunu G, Guga P, Slotow R. Mapping evidence of food safety at transport stations in Africa: a scoping review protocol. BMJ Open. 2020;10(8):e035879-e.

17. Tricco AC, Lillie E, Zarin W, O'Brien KK, Colquhoun H, Levac D, et al. PRISMA extension for scoping reviews (PRISMA-ScR): checklist and explanation. Annals of internal medicine. 2018;169(7):467-73.

18. Moher D, Liberati A, Tetzlaff J, Altman DG, The PRISMA Group. PRISMA 2009 Flow Diagram. 2009;6(2009):1000097.

19. Oguttu JW, McCrindle CME, Makita K, Grace D. Investigation of the food value chain of ready-to-eat chicken and the associated risk for staphylococcal food poisoning in Tshwane Metropole, South Africa. Food Control. 2014;45:87-94.

20. Mafune TS, Takalani TK, Anyasi TA, Ramashia SE. Microbial Safety of Street Vended Foods Sold in Thohoyandou, South Africa. Journal of Human Ecology. 2016;53(3):205-12.

21. Kok R, Balkaran R. Street food vending and hygiene practices and implications for consumers. Journal of Economics Behavioral Studies. 2014;6(3):188-93.

22. Hill J, McHiza Z, Puoane T, Steyn NP. Food sold by street-food vendors in Cape Town and surrounding areas: a focus on food and nutrition knowledge as well as practices related to

For peer review only - http://bmjopen.bmj.com/site/about/guidelines.xhtml

food preparation of street-food vendors. Journal of Hunger & Environmental Nutrition. 2019;14(3):401-15.

23. Mazizi B, Muchenje V, Makepe M, Mutero G. Assessment of Aerobic Plate Counts, Staphylococcus aureus, Escherichia coli and Salmonella in Meat Sold by Street Vendors in the Eastern Cape Province, South Africa. Journal of Food Nutrition Research. 2017;5(6):436-42.

24. Qekwana DN, McCrindle CM, Oguttu JW, Grace D. Assessment of the occupational health and food safety risks associated with the traditional slaughter and consumption of goats in Gauteng, South Africa. International journal of environmental research and public health. 2017;14(4):420.

25. Tshipamba M, Lubanza N, Adetunji M, Mwanza MJJFMSH. Evaluation of the Effect of Hygiene Practices and Attitudes on the Microbial Quality of Street Vended Meats Sold in Johannesburg, South-Africa. 2018;3(137):2476-059.1000137.

26. McArthur-Floyd M, Commey V, Boakye NAB. Evaluation of Food Safety among Fast Food Operators in Madina, Accra. Evaluation. 2016;54.

27. Abakari G, Cobbina SJ, Yeleliere E. Microbial quality of ready-to-eat vegetable salads vended in the central business district of tamale, Ghana. International Journal of Food Contamination. 2018;5(1).

28. Kibret M, Tadesse M. The bacteriological safety and antimicrobial susceptibility of bacteria isolated from street-vended white lupin (Lupinus albus) in Bahir Dar, Ethiopia. Ethiopian Journal of Health Sciences. 2013;23(1):19-26.

29. Eromo T, Tassew H, Daka D, Kibru G. Bacteriological quality of street foods and antimicrobial resistance of isolates in Hawassa, Ethiopia. Ethiopian journal of health sciences. 2016;26(6):533-42.

30. Aluko OO, Ojeremi TT, Olaleke DA, Ajidagba EB. Evaluation of food safety and sanitary practices among food vendors at car parks in Ile Ife, southwestern Nigeria. Food Control. 2014;40:165-71.

31. Odundo A, Okemo P, Chege P. An Assessment of Food Safety Practices among Street Vendors in Mombasa, Kenya. International Journal of Health Sciences Research. 2018;8(5):235-43.

For peer review only

For peer review only - http://bmjopen.bmj.com/site/about/guidelines.xhtml

32. Letuka P, Nkhebenyane J, Thekisoe O. Assessment of Food Safety Knowledge, Attitudes and Practices among Street Food Vendors and Consumers' Perceptions of Street Food Vending in Maseru Lesotho. 2019.

33. Jermini M, Bryan FL, Schmitt R, Mwandwe C, Mwenya J, Zyuulu MH, et al. Hazards and Critical Control Points of Food Vending Operations in a City in Zambia. J Food Prot. 1997;60(3):288-99.

34. Oguttu JW, McCrindle CM, Makita K, Grace D. Investigation of the food value chain of ready-to-eat chicken and the associated risk for staphylococcal food poisoning in Tshwane Metropole, South Africa. Food Control. 2014;45:87-94.

35. Kibret M, Tadesse MJEjohs. The bacteriological safety and antimicrobial susceptibility of bacteria isolated from street-vended white lupin (Lupinus albus) in Bahir Dar, Ethiopia. 2013;23(1):19-26.

36. Gizaw Z. Public health risks related to food safety issues in the food market: a systematic literature review. Environmental Health and Preventive Medicine. 2019;24(1):68.

37. Todd EC. Epidemiology of foodborne diseases: a worldwide review. World health statistics quarterly Rapport trimestriel de statistiques sanitaires mondiales. 1997;50(1-2):30-50.

38. Abrahale K, Sousa S, Albuquerque G, Padrao P, Lunet N. Street food research worldwide: a scoping review. Journal of Human Nutrition and Dietetics. 2019;32(2):152-74.

39. Bellia C, Pilato M, Seraphin H. Street food and food safety: a driver for tourism? Calitatea. 2016;17(S1):20.

40. Mosupye FM, Von Holy A. Microbiological hazard identification and exposure assessment of street food vending in Johannesburg, South Africa. International Journal of Food Microbiology

2000;61(2-3):137-45.

41. Mosupye FM, von Holy A. Microbiological quality and safety of ready-to-eat street-vended foods in Johannesburg, South Africa. International Journal of Food Microbiology. 1999;62(11):1278-84.

For peer review only - http://bmjopen.bmj.com/site/about/guidelines.xhtml

42. Muzaffar AT, Huq I, Mallik BA. Entrepreneurs of the streets: An analytical work on the street food vendors of Dhaka City. International journal of Business Management. 2009;4(2):80-8.

43. World Health Organisation. Universal Health Coverage Geneva2020 [cited 2021 09/04/2021]. Available from: https://www.who.int/health-topics/universal-health-coverage#tab=tab_1.

44. Qekwana DN, McCrindle CM, Oguttu JW, Grace D. Assessment of the occupational health and food safety risks associated with the traditional slaughter and consumption of goats in Gauteng, South Africa. International Journal of Environmental Research. 2017;14(4):420.

45. Melanson K. Nutrition review: lifestyle approaches to promoting healthy eating for children. American journal of lifestyle medicine. 2008;2(1):26-9.

46. Carrier N, Villalon L, Lengyel C, Slaughter SE, Duizer L, Morrison-Koechl J, et al. Diet quality is associated with malnutrition and low calf circumference in Canadian long-term care residents. BMC nutrition. 2019;5:57.

47. Freeland-Graves JH, Nitzke S. Position of the academy of nutrition and dietetics: total diet approach to healthy eating. Journal of the Academy of Nutrition Dietetics. 2013;113(2):307-17.

48. Okojie P, Isah E. Sanitary conditions of food vending sites and food handling practices of street food vendors in Benin City, Nigeria: implication for food hygiene and safety. Journal of environmental public Health Reports. 2014;2014.

49. da Silva SA, Cardoso RdCV, Góes JÂW, Santos JN, Ramos FP, de Jesus RB, et al. Street food on the coast of Salvador, Bahia, Brazil: A study from the socioeconomic and food safety perspectives. Food control. 2014;40:78-84.

50. Acheampong BE. Assessment of food hygiene practices by street food vendors and microbial quality of selected foods sold. A Study at Dunkwa-On-Offin, Upper Denkyira East Municipality of the Central Region 2015.

For peer review only - http://bmjopen.bmj.com/site/about/guidelines.xhtml

Table 1 A: Characteristic of the included sources of evidence

Author, year	Country	City/Town	Study design	Study setting	Study population	Sample size	Sex of vendors	Outcome reported
Oguttu et. al., 2014 [34]	South Africa	Tshwane Metropole, Gauteng Province	Mixed-methods study	Taxi rank	Vendors selling Ready–to-eat chicken	100 samples of Ready–to-eat chicken	Females	Microbial safety of food
Mafune et. al., 2016 [20]	South Africa	Thohoyandou, Limpopo Province	Cross-sectional study	Taxi rank, bus station, shopping mall, and street stalls	Food samples from street vendors	28 samples	Not specified	Microbial safety of food
Kibret et. al., 2013 [28]	Ethiopia	Bahir Dar Town	Cross-sectional study	Main roads sites, bus station, groceries, taxi ranks	Ready–to-eat white lupin sample from vendors	40 samples (200 grams of white lupin)	Not specified	Microbial safety of food
Abakari et. al., 2018 [27]	Ghana	Tamale, Northern Region	Cross-sectional study	Taxi rank, bus stops, transport yard, and timber Market	Ready-to-eat salad samples from food vendors	30 salad samples	Not specified	Microbial safety of food
Aluko et.al., 2014 [30]	Nigeria	Ile Ife, southwestern Nigeria	Cross-sectional study	Car parks	Food vendors	160 (117 stationery and 43 mobile vendors)	Males and females	Hygiene practices of food handlers/vendors
Odundo et. al., 2018 [31]	Kenya	Not specified	Cross-sectional study	Major bus stops, markets, shopping areas, construction sites, and commercial areas	Food vendors	130	Males and females	Hygiene practices of food handlers/vendors
Kok et. al., 2014 [21]	South Africa	Durban, KwaZulu-Natal Province	Cross-sectional study	Transport exchange site	Food vendors	29	Not specified	Hygiene practices of food handlers/vendors
Letuka et. al., 2019 [32]	Lesotho	Maseru	Cross-sectional study	Taxi ranks	Food vendors	141 (48 food handlers and 93 consumers)	Male and female	Knowledge of food safety measures and hygiene practice by food handlers/vendors
Eromo et. al., 2016 [29]	Ethiopia	Hawassa City	Cross-sectional study	Bus station	Food samples from street food vendors	72 samples from six food items	Not specified	Microbial safety of food

For peer review only

For peer review only - http://bmjopen.bmj.com/site/about/guidelines.xhtml

Table 1B: Characteristic of the included sources of evidence

Author, year	Country	City/Town	Study design	Study setting	Study population	Sample size	Sex of vendors	Outcome reported
McArthur-Floyd et. al., 2016 [26]	Ghana	Madina (Accra), Greater Accra Region	Cross-sectional study	Taxi rank, and transport exchange sites	Food vendors	200	Males and females	Knowledge of hygiene practice
Hill et. al., 2019 [6]	South Africa	Cape Town	Cross-sectional study	Train, bus stations, and taxi ranks, community centers, market	Food vendors	831	Males and females	Hygiene practices of food handlers/vendors
Mazizi et. al., 2017 [23]	South Africa	Alice (Nkonkobe) and King William's Town (Buffalo City), Eastern Cape province	Cross-sectional study	Taxi rank and bus stations	Street food vendors	136 food samples- cooked and raw.	Not specified	Microbial safety of food
Qekwana et.al, 2017 [24]	South Africa	Tshwane Metropolitan Municipality, Gauteng Province	Cross-sectional study	Taxi ranks and Informal markets	Traditional goat slaughter	105 people	Males and females	Occupational health and food safety risk
Flego et. al., 2012 [4]	Ghana	Kumasi, Ashanti Region	Cross-sectional study	Bus terminals	Food samples from vendors	60 food samples	Not specified	Microbial safety of food
Tshipamba et. al., 2018 [25]	South Africa	Johannesburg	Cross-sectional study	Taxi ranks and streets	Meat samples from vendors	115 meat samples	Not specified	Hygiene practices of food handlers/vendors, and microbial safety of food
Jermini et. al., 1997 [33]	Zambia	Not specified	Cross-sectional study	bus park/station and large market	Samples of raw, processed, and cooked Foods from street food vendors	Not specified	Not specified	Microbial safety of food

For peer review only - http://bmjopen.bmj.com/site/about/guidelines.xhtml

For peer review only

Table 2 A: Microbial safety of food at transport stations

Study	Type of sample	Analytical approach	Microbes reported	Key results	Key conclusion
Oguttu et. al. [34]	Ready-to-eat (RTE) chicken	3M™ Petrifilm™ plates	*S. aureus*	• High prevalence of *S. aureus* in the sample was (44%; 90% CI: 36.1%-52.2%), with mean *S. aureus* counts of $10^{3.6}$ (90%CI: $10^{3.3}$-$10^{3.9}$).<br>• The likelihood of food poisoning with *S. aureus* from RTE chicken was estimated to be 1.3% (90% CI: 0% -2.7%)	To reduce the levels of concentration of *S. aureus* on the RTE chicken and promote the sale of safer and affordable RTE chicken for the large urban poor population in South Africa, training of RTE chicken vendors on hygiene is still needed.
Mafune et. al. [20]	Unfermented porridge, boiled cabbage and carrots, boiled peanuts, salad, potato chips, traditional mageu, and stewed beef and grilled chicken	Standard microbiological method	*S. aureus*	• *S. aureus* was <2.4771 log10 cfu/g in all samples and places.<br>• Except for fried potato chips, microbial contamination was observed in the remaining food samples using the total plate count method.	Most of the vended foods investigated met the microbiological standard of RTE foods
Mazizi et. al. [23]	Cooked and raw beef, pork, and mutton samples, surface contact plates, and water samples	Biochemical tests according to international standards methods	*S. aureus, E. coli*, and *Salmonella spp.*	• Mean score of raw beef, mutton, and pork were aerobic plate counts (4.8, 3.7 and 2.8 Log (cfu/g)), *S. aureus* (3.3, 3.7 and 2.8 Log cfu/g), and *E. coli* (1.0, 0.6 and 0.3 Log cfu/g) respectively.	The levels of contamination in cooked meat were lower when compared to the standards set by Commission Regulation for determining the microbiological quality of RTE foods.
Tshipamba et. al. [25]	RTE meat	Standard biochemical and Molecular methods	*Bacillus thuringienis, Bacillus spp., Bacillus subtilis, Bacillus cereus, Citrobacter spp., Enterococcus faecium, Enterococcus faecalis, Kurthia spp., Lysinibacillus spp., Macrococcus caseolyticus, Planomicrobium glaciei, Planococcus antarcticus, S. aureus, S. equorum*, and *S. vitulinus*	• Overall mean total bacteria in the samples ranged from 4.3-6.03 cfu/ml × 102 and coliform counts ranged from 1.60-1.95 × 102 cfu/ml<br>• Of the 15 microbes identified, S. aureus occurred in all the meat types and the percentage of occurrence was chicken meat (14%), beef head (43%), beef intestine (50%), and wors (sausage) (20%)	Consumers RTE meat are at risk of food borne diseases due to poor hygiene practices of the vendors.

For peer review only

Table 2 B: Microbial safety of food at transport stations

Study	Type of sample	Analytical approach	Microbes reported	Key results	Key conclusion
Kibret et. al. [28]	White lupin	Standard bacteriological techniques, and Kirby-Bauer disk diffusion method for antimicrobial susceptibility test	*E. coli, Salmonella spp, and Shigella spp.*	• Prevalence of bacteria total coliform counts were 954.2±385 at the surface and 756.2±447.3 at the core of white lupin.<br>• Pathogens isolated were as follows *E. coli* 29 (72.5%), *Salmonella* spp. 23 (57.5%) and *Shigella* spp. 8 (20%).<br>• Overall multiple antimicrobial resistances rate was 75%	Contamination of white lupin and a potential health risk to consumers revealed, and the bacteria isolated showed high rates of multiple drug resistance.
Eromo et. al. [29]	Local bread ('ambasha' and'kita'), raw fish, chilli ('awaze'), avocado, and cooked potato	Standard microbiological techniques	*E. scoli, Salmonella spp.*, and *S. aureus*	• The microbiological quality in nearly 31% of RTE food samples was beyond the acceptable limits.<br>• Total colony counts detected ranged from 1.7x105 to 6.7x106 cfu/g.<br>• *E.coli* (29.6%), *Salmonella* spp. (12.7%, and *S. aureus* (9.9%) were the most frequent isolates.<br>• All isolates were 100% sensitive to ciprofloxacin, but 89% of *Salmonella* spp. was resistant to chloramphenicol, 14.3% of *S.aureus* was resistant to vancomycin	Considerable rate of contamination in the foods confirmed. The identified foodborne bacteria and antibiotic resistance isolates could pose a public health problem in the study location.
Abakari et. al. [27]	Pre-cut vegetable salads	Standard microbiological methods	*E. coli, Bacillus cereus, Salmonella spp, and Shigella spp.*	• *E. coli* levels ranged from 0 to 7.56 log10 cfu/g; *Bacillus cereus* levels ranged from 0 to 7.44 log10 cfu/g; *Salmonella* spp. ranged from 0 to 4.54 log10 cfu/g, and *Shigella* spp. ranged from 5.54 log10 cfu/g were detected in 96.7%, 93.3%, 73.3%, and 76.7% of the salads samples, respectively.	Salads were revealed to be unwholesome for human consumption and could be deleterious to the health of consumers.

For peer review only

Table 2 C: Microbial safety of food at transport stations

Study	Type of sample	Analytical approach	Microbes reported	Key results	Key conclusion
Flego et. al. [4]	RTE foods (ice-kenkey (15), cocoa drink (15), fufu (5), ready-to-eat red pepper for kenkey) (5), salad (10), and macaroni (10))	Standard microbiological methods	*Staphylococci*, *Bacillus* spp., *Klebsiella pneumoniae*, *Aeromonas pneumophila*, *E. cloacae*, *S. aureus*, *E. coli*, and *P. aeruginosa*	• RTE foods were found to be contaminated with enteric bacteria and other potential food poisoning organisms with bacterial counts higher than the acceptable levels (<5.0 log10 CFU/ml).<br>• Coagulate negative staphylococci (23.7%), Bacillus species (21.5%), *K. pneumoniae* (18.0%), *Aeromonas pneumophila* (17.7%), E. cloacae (6.7%), *S. aureus* (3.7%), E. coli (2.2%) and *P. aeruginosa* (2.2%) were the main isolates detected.	Most RTE foods were contaminated with enteric bacteria and other potential food poisoning organisms with bacterial counts higher than the acceptable levels.
Jermini et. al. [33]	Raw foods (ground meat, chicken, and chicken intestine); and processed foods (dried "minnows" and "kapenta")		*Salmonellae Spp.*, *S. aureus*, *Clostridium peifringens*	• Raw foods such as ground meat, chicken, chicken intestine; and processed foods such as dried "minnows" and "kapenta" were contaminated by salmonellae or contained high populations of S. aureus in pasteurized milk.<br>• High populations (> 105) of S. aureus were detected from a sample of leftover chicken, more than 107 were detected in leftover rice, and 10 million *C. peifringens* per gram were detected in leftover beef stew sample	Time-temperature exposures during reheating had variable effects in terms of killing the microorganisms that germinated from surviving spores or that reached the foods after cooking.

For peer review only

540 **Table 3:** Key reported findings on food preparation

Study	Key findings reported
Kok et. al. [21]	• Water being used for washing utensils was left unchanged,<br>• Piles of dirty pots and dishes was left near the serving areas and RTE foods, and garbage left uncovered with many flies at the site,<br>• RTE food was left uncovered,<br>• Most of the food handlers were not wearing gloves, hairnets, or aprons
Hill et. al. [6]	• 85.5% of the vending stalls lacked soap or surface sanitizer,<br>• 71% lacked basin for washing,<br>• 75% did not have drying cloth,<br>• 76.6% of vendors handled food and money concurrently,<br>• About 57% left the food uncovered.<br>• 39% of the vendors were using their hands to pick up food items, with only 6% wearing gloves, and<br>• 29% of vendors had a wet clean sponge/cloth obtainable at the site
Mazizi et. al. [23]	• Major sources of food contamination identified were poor hygiene practices of the food vendor, holding area, and the utensils
Tshipamba et. al. [25]	• Approximately 90% of RTE meat vendors at the taxi rank exposed their meats to dust and flies,<br>• 94% of them handled money whilst serving food, and<br>• Stagnant water found in about 22% of the vending locations at the taxi-rank
McArthur-Floyd et. al. [26]	• 64% of food vendors washed their hands from elbow to finger and the remainder (36%) washed from their wrist to finger (the WHO recommends handwashing from elbow to fingers), and<br>• 62% of the vendors test their meal in the palm whilst 38% of them test it with a spoon (the best way to test a meal)
Aluko et. al. [30]	• Approximately 17% of food vendors washed their hands always after using the toilet,<br>• 63% of them rarely kept their fingernails short, and<br>• Nearly 4% of them always kept their leftover cooked food in a refrigerator, despites having unstable power supply
Odundo et. al. [31]	• Food vendors had poor hygiene practices however, men were observed to have better hygienic practices than women ($P<0.05$),<br>• Hygiene practice of the vendors was found to be significantly associated with training (those trained observe hygiene), and<br>• Wearing of jewellery, long and unclean nails, and lack of protective clothing were observed.
Letuka et. al. [32]	• Observed that the food handlers operated under unhygienic environment

541

542

543 **Figure**

544 **Figure 1:** PRISMA 2009 Flow Diagram

545 **Figure 2:** Trend of published studies relating to food safety at transport station in Africa

546

547 **Supplementary File**

548 **Supplementary file 1:** Electronic databases search

For peer review only - http://bmjopen.bmj.com/site/about/guidelines.xhtml

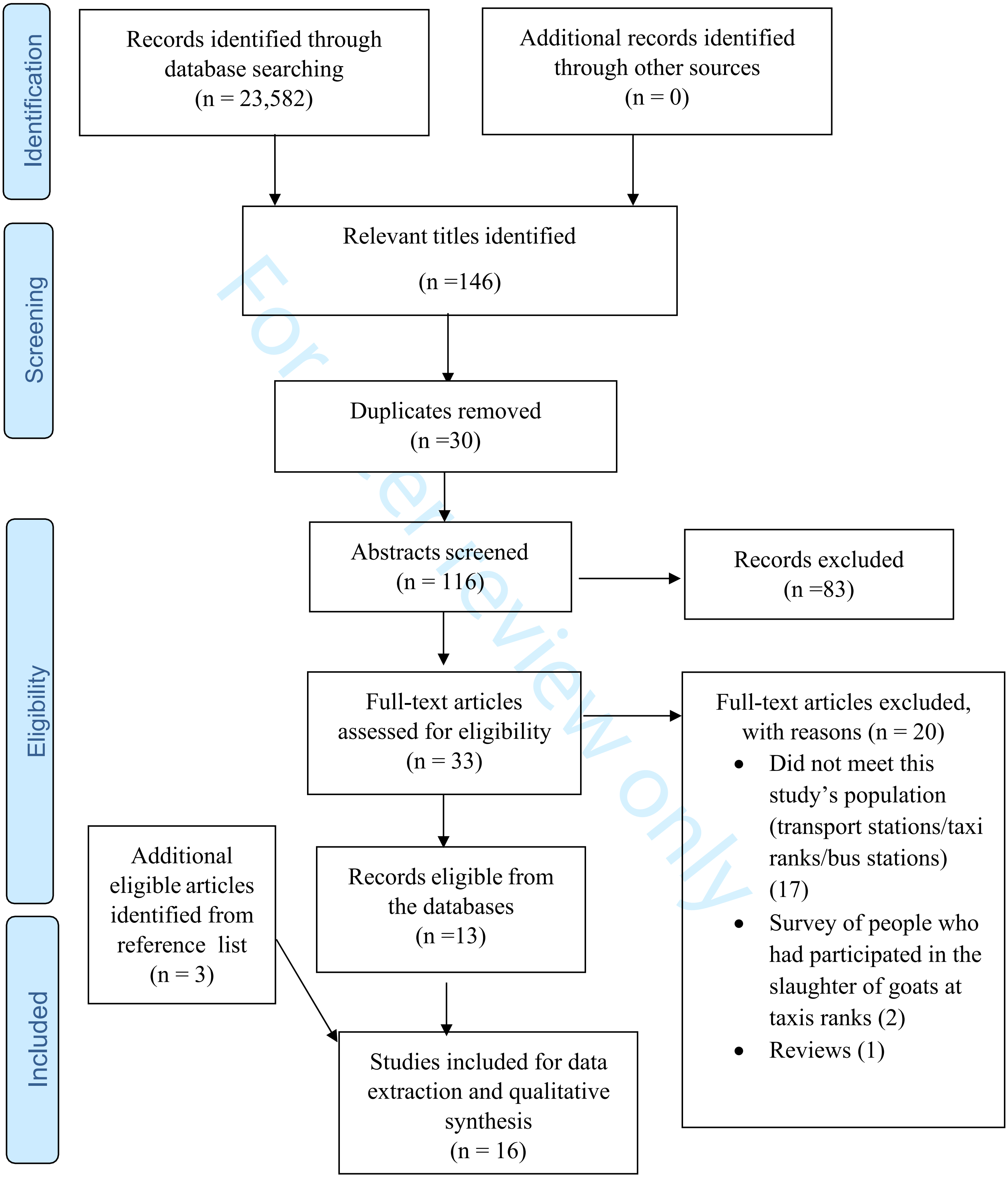

Figure 1: PRISMA 2009 Flow Diagram [18]

For peer review only

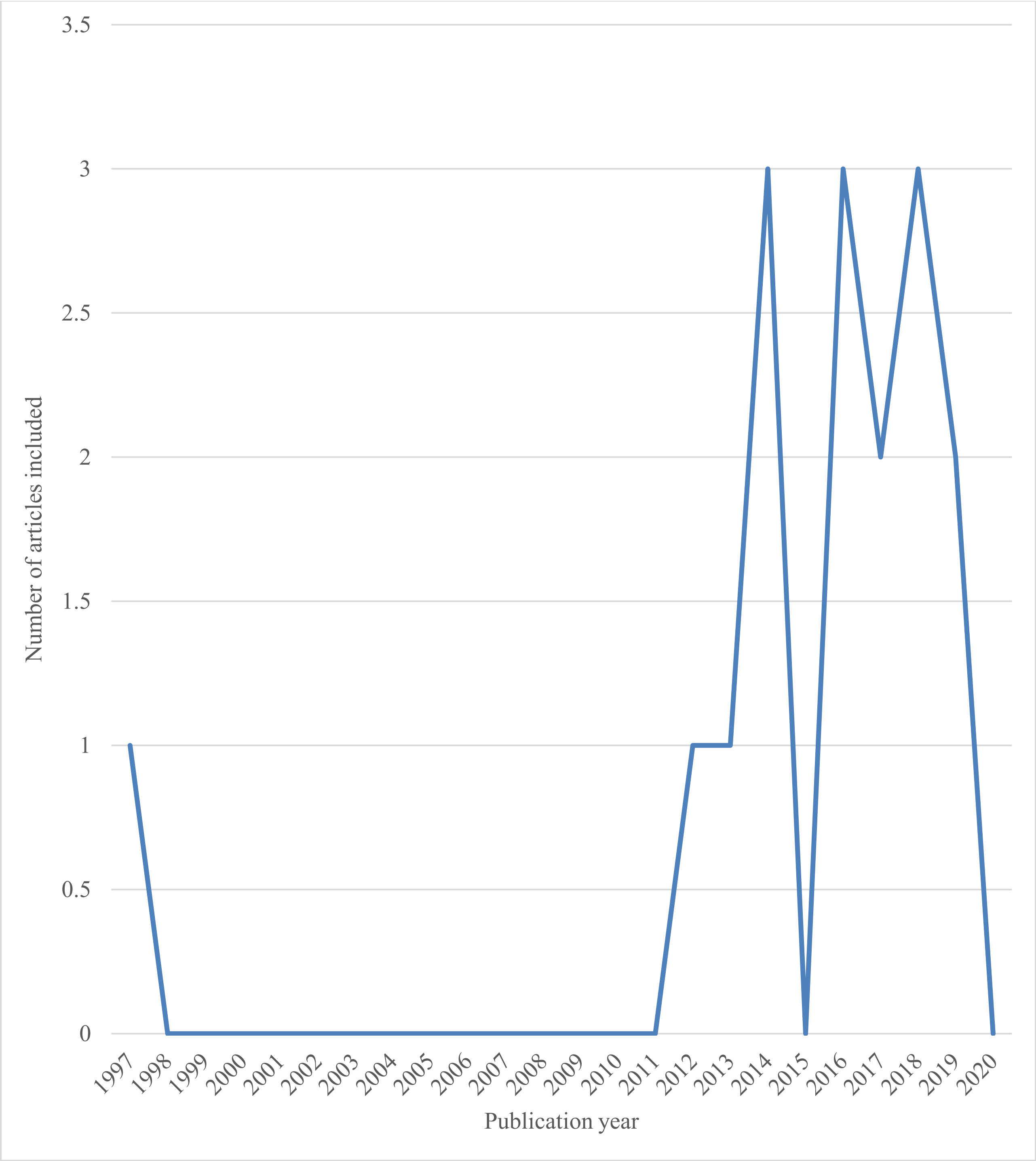

Figure 2: Trend of published studies relating to food safety at transport stations in Africa

Supplementary file 1: Electronic databases search

Search date	Database	Keywords	Search results	Number eligible
05/10/2020	EBSCOhost (Academic search complete, CINAHL with Full-text, and Health Source	SU food safety AND SU (food preparation or meal preparation or cooking) OR SU food handling OR SU food storage OR hygiene practices AND (food trading or food selling or food vending or street food) AND (transport station or taxi rank or bus station or transport exchange sites or car park or lorry park) AND africa	2,549	14
07/10/2020	PubMed	"food safety"[MeSH Terms] OR ("food"[All Fields] AND "safety"[All Fields]) OR "food safety"[All Fields]) OR ("food supply"[MeSH Terms] OR ("food"[All Fields] AND "supply"[All Fields]) OR "food supply"[All Fields] OR ("food"[All Fields] AND "security"[All Fields]) OR "food security"[All Fields]) AND (("food"[MeSH Terms] OR "food"[All Fields]) AND vending[All Fields]) OR (("food"[MeSH Terms] OR "food"[All Fields]) AND trading[All Fields]) AND streets[All Fields] OR (("motor vehicles"[MeSH Terms] OR ("motor"[All Fields] AND "vehicles"[All Fields]) OR "motor vehicles"[All Fields] OR "lorry"[All Fields]) AND parks[All Fields]) OR (("motor vehicles"[MeSH Terms] OR ("motor"[All Fields] AND "vehicles"[All Fields]) OR "motor vehicles"[All Fields] OR "lorry"[All Fields]) AND station[All Fields]) OR (taxi[All Fields] AND ranks[All Fields]) AND ("algeria"[MeSH Terms] OR "algeria"[All Fields]) OR ("angola"[MeSH Terms] OR "angola"[All Fields]) OR ("benin"[MeSH Terms] OR "benin"[All Fields]) OR ("botswana"[MeSH Terms] OR "botswana"[All Fields]) OR ("burkina faso"[MeSH Terms] OR ("burkina"[All Fields] AND "faso"[All Fields]) OR "burkina faso"[All Fields]) OR ("burundi"[MeSH Terms] OR "burundi"[All Fields]) OR ("cameroon"[MeSH Terms] OR "cameroon"[All Fields]) OR ("cabo verde"[MeSH Terms] OR ("cabo"[All Fields] AND "verde"[All Fields]) OR "cabo verde"[All Fields] OR ("cape"[All Fields] AND "verde"[All Fields]) OR "cape verde"[All Fields]) OR ("central african republic"[MeSH Terms]	2,834	33

		OR ("central"[All Fields] AND "african"[All Fields] AND "republic"[All Fields]) OR "central african republic"[All Fields]) OR ("chad"[MeSH Terms] OR "chad"[All Fields]) OR ("democratic republic of the congo"[MeSH Terms] OR ("democratic"[All Fields] AND "republic"[All Fields] AND "congo"[All Fields]) OR "democratic republic of the congo"[All Fields]) OR ("congo"[MeSH Terms] OR "congo"[All Fields] OR ("republic"[All Fields] AND "congo"[All Fields]) OR "republic of the congo"[All Fields]) OR ("djibouti"[MeSH Terms] OR "djibouti"[All Fields]) OR ("egypt"[MeSH Terms] OR "egypt"[All Fields]) OR ("equatorial guinea"[MeSH Terms] OR ("equatorial"[All Fields] AND "guinea"[All Fields]) OR "equatorial guinea"[All Fields]) OR ("eritrea"[MeSH Terms] OR "eritrea"[All Fields]) OR ("ethiopia"[MeSH Terms] OR "ethiopia"[All Fields]) OR ("gabon"[MeSH Terms] OR "gabon"[All Fields]) OR ("gambia"[MeSH Terms] OR "gambia"[All Fields]) OR ("ghana"[MeSH Terms] OR "ghana"[All Fields]) OR ("guinea"[MeSH Terms] OR "guinea"[All Fields]) OR ("guinea-bissau"[MeSH Terms] OR "guinea-bissau"[All Fields] OR ("guinea"[All Fields] AND "bissau"[All Fields]) OR "guinea bissau"[All Fields]) OR ("cote d'ivoire"[MeSH Terms] OR ("cote"[All Fields] AND "d'ivoire"[All Fields]) OR "cote d'ivoire"[All Fields] OR ("ivory"[All Fields] AND "coast"[All Fields]) OR "ivory coast"[All Fields]) OR ("kenya"[MeSH Terms] OR "kenya"[All Fields]) OR ("lesotho"[MeSH Terms] OR "lesotho"[All Fields]) OR ("liberia"[MeSH Terms] OR "liberia"[All Fields]) OR ("libya"[MeSH Terms] OR "libya"[All Fields]) OR ("madagascar"[MeSH Terms] OR "madagascar"[All Fields]) OR ("malawi"[MeSH Terms] OR "malawi"[All Fields]) OR ("mali"[MeSH Terms] OR "mali"[All Fields]) OR ("mauritania"[MeSH Terms] OR "mauritania"[All Fields]) OR ("mauritius"[MeSH Terms] OR "mauritius"[All Fields]) OR ("morocco"[MeSH Terms] OR "morocco"[All Fields]) OR ("mozambique"[MeSH Terms] OR "mozambique"[All Fields]) OR ("namibia"[MeSH Terms] OR "namibia"[All Fields]) OR ("niger"[MeSH Terms] OR "niger"[All Fields]) OR ("nigeria"[MeSH Terms] OR "nigeria"[All Fields]) OR ("rwanda"[MeSH Terms] OR		

For peer review only

		"rwanda"[All Fields]) OR ("sao tome and principe"[MeSH Terms] OR ("sao"[All Fields] AND "tome"[All Fields] AND "principe"[All Fields]) OR "sao tome and principe"[All Fields]) OR ("senegal"[MeSH Terms] OR "senegal"[All Fields]) OR ("seychelles"[MeSH Terms] OR "seychelles"[All Fields]) OR ("sierra leone"[MeSH Terms] OR ("sierra"[All Fields] AND "leone"[All Fields]) OR "sierra leone"[All Fields]) OR ("somalia"[MeSH Terms] OR "somalia"[All Fields]) OR (("south africa"[MeSH Terms] OR ("south"[All Fields] AND "africa"[All Fields]) OR "south africa"[All Fields]) AND South[All Fields]) OR ("sudan"[MeSH Terms] OR "sudan"[All Fields]) OR ("sudan"[MeSH Terms] OR "sudan"[All Fields]) OR ("eswatini"[MeSH Terms] OR "eswatini"[All Fields] OR "swaziland"[All Fields]) OR ("tanzania"[MeSH Terms] OR "tanzania"[All Fields]) OR ("togo"[MeSH Terms] OR "togo"[All Fields]) OR ("tunisia"[MeSH Terms] OR "tunisia"[All Fields]) OR ("uganda"[MeSH Terms] OR "uganda"[All Fields]) OR ("zambia"[MeSH Terms] OR "zambia"[All Fields]) OR ("zimbabwe"[MeSH Terms] OR "zimbabwe"[All Fields]) Filters: Free full text, Comparative Study, Observational Study, Randomized Controlled Trial, Humans, English, MEDLINE		
09/10/2020	Web of Science	(food safety AND food preparation OR meal preparation OR cooking OR food handling OR food storage OR hygiene practices AND food trading OR food selling OR food vending or street food AND transport station OR taxi rank OR bus station OR transport exchange sites OR car park or lorry park AND africa)Refined by: Open Access: (OPEN ACCESS) AND DOCUMENT TYPES: (ARTICLE) AND DOCUMENT TYPES: (ARTICLE) AND WEB OF SCIENCE CATEGORIES: (FOOD SCIENCE TECHNOLOGY OR PUBLIC ENVIRONMENTAL OCCUPATIONAL HEALTH) AND WEB OF SCIENCE CATEGORIES: (FOOD SCIENCE TECHNOLOGY OR PUBLIC ENVIRONMENTAL OCCUPATIONAL HEALTH OR NUTRITION DIETETICS)Timespan: All years. Indexes: SCI-EXPANDED, SSCI, A&HCI, CPCI-S, CPCI-SSH, BKCI-S, BKCI-SSH, ESCI.	8,263	36
18/10/2020	SCOPUS	food safety AND food preparation OR meal preparation OR cooking OR food handling OR food storage OR hygiene practices AND food trading OR	116	7

For peer review only

		food selling OR food vending or street food AND transport station OR taxi rank OR bus station OR transport exchange sites OR car park or lorry park AND africa		
25/10/2020	Google Scholar	food safety AND food preparation OR meal preparation OR cooking OR food handling OR food storage OR hygiene practices AND food trading OR food selling OR food vending or street food AND transport station OR taxi rank OR bus station OR transport exchange sites OR car park or lorry park AND africa	9,820	56
			23,582	146
**Duplicates**				**30**

For peer review only

Supplementary file 1: Preferred Reporting Items for Systematic reviews and Meta-Analyses extension for Scoping Reviews (PRISMA-ScR) Checklist

SECTION	ITEM	PRISMA-ScR CHECKLIST ITEM	REPORTED ON PAGE #
**TITLE**			
Title	1	Identify the report as a scoping review.	
**ABSTRACT**			
Structured summary	2	Provide a structured summary that includes (as applicable): background, objectives, eligibility criteria, sources of evidence, charting methods, results, and conclusions that relate to the review questions and objectives.	
**INTRODUCTION**			
Rationale	3	Describe the rationale for the review in the context of what is already known. Explain why the review questions/objectives lend themselves to a scoping review approach.	
Objectives	4	Provide an explicit statement of the questions and objectives being addressed with reference to their key elements (e.g., population or participants, concepts, and context) or other relevant key elements used to conceptualize the review questions and/or objectives.	
**METHODS**			
Protocol and registration	5	Indicate whether a review protocol exists; state if and where it can be accessed (e.g., a Web address); and if available, provide registration information, including the registration number.	
Eligibility criteria	6	Specify characteristics of the sources of evidence used as eligibility criteria (e.g., years considered, language, and publication status), and provide a rationale.	
Information sources*	7	Describe all information sources in the search (e.g., databases with dates of coverage and contact with authors to identify additional sources), as well as the date the most recent search was executed.	
Search	8	Present the full electronic search strategy for at least 1 database, including any limits used, such that it could be repeated.	
Selection of sources of evidence†	9	State the process for selecting sources of evidence (i.e., screening and eligibility) included in the scoping review.	
Data charting process‡	10	Describe the methods of charting data from the included sources of evidence (e.g., calibrated forms or forms that have been tested by the team before their use, and whether data charting was done independently or in duplicate) and any processes for obtaining and confirming data from investigators.	
Data items	11	List and define all variables for which data were sought and any assumptions and simplifications made.	
Critical appraisal of individual sources of evidence§	12	If done, provide a rationale for conducting a critical appraisal of included sources of evidence; describe the methods used and how this information was used in any data synthesis (if appropriate).	
Synthesis of results	13	Describe the methods of handling and summarizing the data that were charted.	

St.Michael's
Inspired Care.
Inspiring Science.

For peer review only - http://bmjopen.bmj.com/site/about/guidelines.xhtml

SECTION	ITEM	PRISMA-ScR CHECKLIST ITEM	REPORTED ON PAGE #
**RESULTS**			
Selection of sources of evidence	14	Give numbers of sources of evidence screened, assessed for eligibility, and included in the review, with reasons for exclusions at each stage, ideally using a flow diagram.	
Characteristics of sources of evidence	15	For each source of evidence, present characteristics for which data were charted and provide the citations.	
Critical appraisal within sources of evidence	16	If done, present data on critical appraisal of included sources of evidence (see item 12).	
Results of individual sources of evidence	17	For each included source of evidence, present the relevant data that were charted that relate to the review questions and objectives.	
Synthesis of results	18	Summarize and/or present the charting results as they relate to the review questions and objectives.	
**DISCUSSION**			
Summary of evidence	19	Summarize the main results (including an overview of concepts, themes, and types of evidence available), link to the review questions and objectives, and consider the relevance to key groups.	
Limitations	20	Discuss the limitations of the scoping review process.	
Conclusions	21	Provide a general interpretation of the results with respect to the review questions and objectives, as well as potential implications and/or next steps.	
**FUNDING**			
Funding	22	Describe sources of funding for the included sources of evidence, as well as sources of funding for the scoping review. Describe the role of the funders of the scoping review.	

JBI = Joanna Briggs Institute; PRISMA-ScR = Preferred Reporting Items for Systematic reviews and Meta-Analyses extension for Scoping Reviews.

* Where *sources of evidence* (see second footnote) are compiled from, such as bibliographic databases, social media platforms, and Web sites.

† A more inclusive/heterogeneous term used to account for the different types of evidence or data sources (e.g., quantitative and/or qualitative research, expert opinion, and policy documents) that may be eligible in a scoping review as opposed to only studies. This is not to be confused with *information sources* (see first footnote).

‡ The frameworks by Arksey and O'Malley (6) and Levac and colleagues (7) and the JBI guidance (4, 5) refer to the process of data extraction in a scoping review as data charting.

§ The process of systematically examining research evidence to assess its validity, results, and relevance before using it to inform a decision. This term is used for items 12 and 19 instead of "risk of bias" (which is more applicable to systematic reviews of interventions) to include and acknowledge the various sources of evidence that may be used in a scoping review (e.g., quantitative and/or qualitative research, expert opinion, and policy document).

From: Tricco AC, Lillie E, Zarin W, O'Brien KK, Colquhoun H, Levac D, et al. PRISMA Extension for Scoping Reviews (PRISMA-ScR): Checklist and Explanation. Ann Intern Med. ;169:467–473. doi: 10.7326/M18-0850

For peer review only
